# Provenance-Associated Confounding in Cross-Collection Comparisons of Urease Inhibitors: A Quantified Reference Scale and a Domain-Aware Alternative

**DOI:** 10.3390/ijms27209159

**Published:** 2026-10-15

**Authors:** Javier Farías-Abarca, Gabriela Urra, Constanza Henríquez, Osvaldo Yáñez, Manuel I. Osorio, Jonathan Palma, Luis Morales-Quintana, Reynier Suardíaz, Daniel Bustos

**Affiliations:** 1 Laboratorio de Bioinformática y Química Computacional, Departamento de Medicina Traslacional, Facultad de Medicina, Universidad Católica del Maule, Talca 3480094, Chile; javier.farias@alu.ucm.cl (J.F.-A.); gabriela.urra@utalca.cl (G.U.); henriquezvera.95@gmail.com (C.H.); 2 Doctoral Program in Sciences with a Specialization in Modeling of Chemical and Biological Systems, Faculty of Engineering, Universidad de Talca, Talca 3460000, Chile; 3 Centro de Modelación Ambiental y Dinámica de Sistemas (CEMADIS), Facultad de Ingeniería y Negocios, Universidad de Las Américas, Santiago 7500975, Chile; oyanez@udla.cl; 4 Facultad de Odontología, Universidad Andrés Bello, Santiago Chile, Echaurren 237, Santiago 8370133, Chile; manuel.osorio@unab.cl; 5 Centro de Investigación Biomédica, Facultad de Medicina, Universidad Diego Portales, Ejército 141, Santiago 8370007, Chile; 6 Facultad de Ingeniería, Universidad de Talca, Maule, Curicó 3349001, Chile; jonathan.palma@utalca.cl; 7 Multidisciplinary Agroindustry Research Laboratory, Instituto de Ciencias Biomédicas, Facultad de Ciencias de La Salud, Universidad Autónoma de Chile, Cinco Poniente #1670, Región del Maule, Talca 7500912, Chile; luis.morales@uautonoma.cl; 8 Departamento de Química Física, Facultad de Ciencias Químicas, Universidad Complutense de Madrid, 28040 Madrid, Spain; 9 Laboratorio de Genómica, Bioinformática y Biosensores Aplicados, Facultad de Medicina, Universidad Católica del Maule, Talca 3480094, Chile

**Keywords:** chemical space, dataset bias, applicability domain, conformal prediction, urease inhibitors, benchmark design, model validation, provenance-associated confounding

## Abstract

Reference-library comparisons are widely used in target-focused cheminformatics to characterize the chemical space occupied by bioactive compounds. The separation obtained from such comparisons may, however, reflect differences in library provenance rather than target-related chemistry. The present study quantifies this effect using UI-Ref, a curated reference set of 1218 compounds reported in urease assays in ChEMBL, BindingDB and PubChem, for which assay- and document-level provenance was retained. Within this single target-focused collection, a random forest trained on 25 physicochemical descriptors predicted the depositing database with a receiver operating characteristic area under the curve (ROC AUC) of 0.954 (95% confidence interval, CI, 0.939–0.968) and the source publication with a median one-versus-rest AUC of 0.997, providing an empirical reference scale for provenance-associated separability. Under a conventional pairwise design, comparisons between UI-Ref and external reference libraries yielded AUCs of 0.956–0.982. After property matching and publication-grouped cross-validation, the corresponding AUCs decreased to 0.762–0.841. Because the two tasks differ in terms of class priors, sample size and validation structure, this reference scale is reported as a diagnostic covariate rather than as a decision threshold, and the residual separation was compared against null models constructed within the same design. A null using only features that describe a molecule’s relative position within its source library, rather than its absolute physicochemical descriptor values, reached AUCs of 0.995–0.996 in that same task and exceeded the observed models by 0.154–0.234 (paired bootstrap; DeLong Z = −20.1 to −24.6). Models predicting an administrative provenance label within an external library achieved AUCs of 0.740–0.749, close to but below the 0.762–0.841 range obtained for collection membership. This similarity indicates that descriptor-based predictability is not specific to target-related chemistry. The residual separation exceeded the within-pair label-permutation null by 0.263–0.341, which indicates a detectable difference; however, the design cannot distinguish target-related chemistry from residual provenance structure. Following conditioning on provenance predictability, approximately 1% of compound pairs remained comparable. Matching on four physicochemical properties, moreover, left 25 to 27 of the 39 covariates visible to the models imbalanced, including thiourea and chelating motifs. Nearest-neighbor rankings changed after sample-size balancing, and the apparent overlap with approved drugs corresponded to a median nearest-neighbor Tanimoto similarity of only 0.355. Clusters in the joint chemical spaces were associated more strongly with source publication than with collection membership (adjusted mutual information 0.814 versus 0.187, respectively). No association between a structural motif and potency survived hierarchical estimation using a document-level random intercept and false discovery rate control. A revised classification cascade was therefore developed, incorporating explicit label definitions, probability calibration, a prespecified applicability domain and conformal prediction. Between 92.8% and 97.0% of the compounds in each external library fell outside the ECFP4-based applicability domain, and only 10.3% of an external screening cohort completed the cascade with an informative prediction. The measured-active-against-measured-inactive stage is reported as a narrow-scope diagnostic rather than as a general predictive stage, and every model-based stage reports empirical coverage without a conformal guarantee, because exchangeability across source publications is rejected. Provenance-associated confounding can therefore dominate conventional cross-collection comparisons and should be quantified before chemical or biological interpretations are drawn. The curated data, analysis code, trained models and domain-aware application are publicly available.

## 1. Introduction

Urease (urea amidohydrolase, EC 3.5.1.5) is a nickel-dependent metalloenzyme that hydrolyzes urea to ammonia and carbamate. It is a virulence factor in ureolytic pathogens and a target of agronomic interest [1,2]. In *Helicobacter pylori* (*H. pylori*), the resulting local pH buffering permits gastric colonization, and in *Proteus mirabilis*, urease-driven alkalinization promotes struvite stone formation [1,3]. Clinical precedent for inhibition is provided by acetohydroxamic acid, whose use is limited by its tolerability, and several decades of medicinal chemistry have yielded hydroxamates, thioureas, polyphenols and organophosphorus scaffolds without delivering a second approved agent [4,5].

This accumulated literature has motivated cheminformatic surveys of the inhibitor landscape. Aniceto et al. assembled and characterized 3200 compounds tested against urease, describing scaffolds, property distributions and activity cliffs; comparing the set against DrugBank; and extracting interpretable activity patterns with a decision tree [6]. The same group subsequently combined machine learning with molecular docking to prioritize virtual screening hits [7]. More recent work has added metadynamics and dimensionality reduction to triazole-focused design [8]. A recurrent design in this literature, and in target-focused cheminformatics well beyond urease, is cross-collection comparison, in which the inhibitors of a target are placed alongside a pharmacological library, a natural-product collection, and a toxicological inventory, and the resulting separations and overlaps are interpreted as statements about the chemistry of the target. The conclusion typically drawn is that the inhibitors occupy a drug-like region of chemical space and remain separated from toxicological chemical space.

The present study examines whether that design can support such a conclusion and quantifies the magnitude of the confounding involved. Reference-library membership does not define a chemical class. ChEMBL [9,10], BindingDB [11] and PubChem [12] aggregate medicinal-chemistry publications; CLUE curates compounds with a clinical history [13]; COCONUT aggregates natural products [14]; and DSSTox indexes substances of environmental and toxicological interest [15,16]. Chemistry is central to how each of these resources is built: compounds are identified, standardized, indexed and organized by structure, and several resources compute and distribute molecular descriptors of their own (Table 1). Each library, nevertheless, also reflects the sampling process that produced it, including which laboratories deposited records, under which publication conventions the records were published, and over which period the records were deposited. A classifier trained to separate the inhibitors of a target from a reference library can therefore solve the easier task of recognizing deposition provenance, whereas the conventional design cannot determine whether classification is based on provenance rather than target-related chemistry.

The existence of such shortcuts is not itself a new observation. The virtual-screening community has long recognized that decoy construction determines apparent enrichment, which has motivated property-matched decoys and unbiased benchmark construction [17,18,19,20]. Molecular machine learning has documented that random splits overstate generalization relative to scaffold-based or temporal splits [21,22,23]. Applicability-domain estimation characterizes the region of descriptor and structural space that is represented in the training data and therefore indicates whether a given prediction constitutes interpolation or extrapolation; conformal prediction provides prediction sets with a specified coverage level under an exchangeability assumption [24,25,26,27,28]. Neither procedure establishes that a prediction is mechanistically relevant, which also depends on factors such as the chemical mechanism of inhibition and the assay system used. Cross-library chemical-space comparisons, however, still lack a measured reference scale: an estimate of the separation generated by provenance-associated structure alone within the dataset under analysis, relative to which a reported AUC can be interpreted. At present, the only available comparator is 0.5.

Previous work has provided a valuable descriptive characterization of the urease-inhibitor chemical space [6]. Building on that foundation, the present study addresses the methodological influence of library provenance. Specifically, this study (i) quantifies the extent to which provenance-associated structure alone supports discrimination within a single target-focused collection, (ii) determines how cross-library discriminative performance changes when property-matched negatives and publication-grouped validation are introduced and compares the residual performance against task-matched null models, (iii) evaluates how sample size and projection method affect chemical-space interpretations and how publication clustering influences the association between structural motifs and potency, and (iv) develops a calibrated, domain-aware classifier cascade with explicit label definitions, implemented as reproducible software that withholds predictions for compounds outside its applicability domain. The distinction carries practical consequences beyond urease. Cross-library separations of this kind are routinely cited as evidence that the inhibitors of a target occupy a privileged, drug-like region of chemical space, a conclusion that can influence compound prioritization, claims of novelty and comparisons against toxicologically flagged substances. Where such separations instead reflect deposition provenance, these downstream decisions may propagate an error that the reported AUC alone cannot reveal. A measured provenance reference scale and an explicit applicability domain therefore serve two purposes: they constrain the interpretation of specific claims made about the urease-inhibitor chemical space, and they provide a general, computationally inexpensive diagnostic that other target-focused cheminformatics studies can apply before chemical or biological conclusions are drawn from library comparisons.

## 2. Results

### 2.1. Composition of the Curated Urease Reference Set

**Figure 1:**
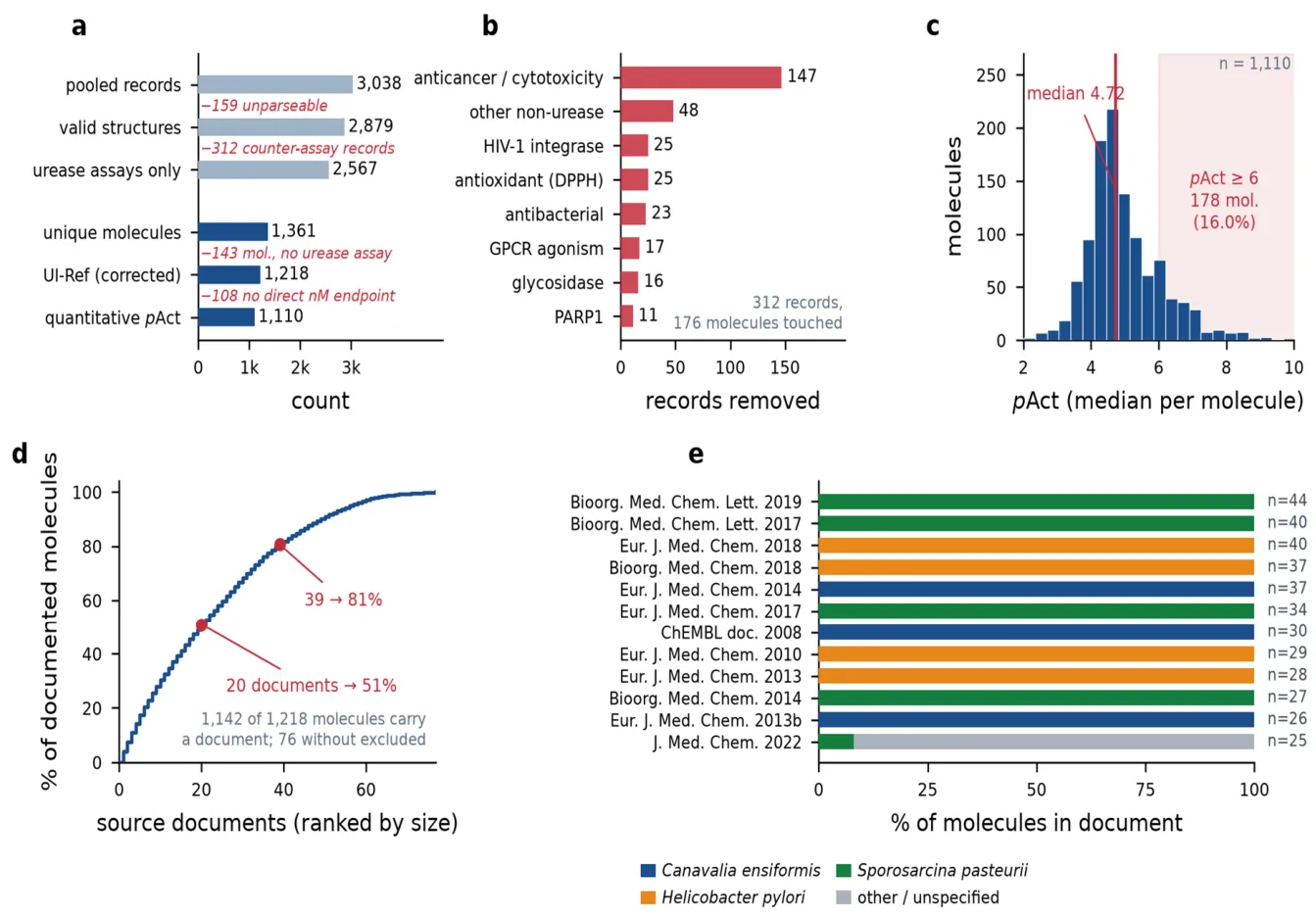
Composition and provenance structure of UI-Ref, a reported-activity set of 1218 urease-assayed molecules. (**a**) Curation cascade from the pooled PubChem, BindingDB and ChEMBL exports to the molecule-level reference set; grey bars denote assay records, and dark bars denote unique molecules. Two filters absent from the uncorrected pipeline account for most of the change: restriction to records from a urease assay removes 312 counter-assay records and 143 molecules, yielding the 1218 molecules of UI-Ref, and the further requirement of a direct nanomolar endpoint removes 108 of those, leaving the 1110 that carry a quantitative endpoint. On an inclusion path that discards every record reported as inactive or inconclusive, the urease-assay restriction alone removes 93 molecules. (**b**) Endpoints measured by the removed records: the 312 non-urease records are dominated by anticancer and cytotoxicity assays, antioxidant (DPPH) assays, and enzyme counter-screens; they involve 176 molecules in total, 143 of which had no urease record. (**c**) Distribution of median pAct over the 1110 molecules with a quantitative endpoint (per-molecule medians of replicate values, anchored in nanomolar units). Median pAct is 4.72 and 178 molecules (16.0%) reach pAct >=6. (**d**) Cumulative share of the 1142 documented molecules against source documents ranked by size; molecules without a source document are excluded from both axes (77 named documents). (**e**) Organism composition of the twelve largest documents.

The reference set was reconstructed directly from the original database exports, with assay-level provenance preserved throughout, and a uniform standardization workflow was applied to all collections, comprising largest-fragment selection, functional-group normalization, canonical tautomer generation and deduplication by International Chemical Identifier key (InChIKey) [29]. This record-level reconstruction materially altered the composition of the final dataset (Figure 1a; Supplementary File, Tables S1–S3).

The curation flow is summarized in Figure 1a and in Supplementary File, Table S1. The three primary database exports contained 3038 assay records. After structure standardization, 2879 records were retained, representing 1361 unique molecules. Subsequent filters were applied at both the record and the molecule levels. Restriction to confirmed urease assays removed 312 counter-assay records and 143 unique molecules, reducing the dataset from 1361 to 1218 molecules. Of these, 1110 had at least one direct quantitative endpoint reported in nanomolar units, comprising the half-maximal inhibitory concentration (IC_50_), the inhibition constant (K_i_), the dissociation constant (K_d_) or the half-maximal effective concentration (EC_50_), whereas 108 lacked a qualifying quantitative measurement. The molecule-level progression was therefore 1361 after structure standardization, 1218 after restriction to confirmed urease assays and 1110 after the requirement of a direct quantitative endpoint.

Three curation issues account for the difference between the corrected and the uncorrected dataset. First, 93 molecules had entered the original dataset solely through assays unrelated to urease, including human cell cytotoxicity assays, human immunodeficiency virus type 1 (HIV-1) integrase inhibition assays and 2,2-diphenyl-1-picrylhydrazyl (DPPH) radical-scavenging counter-assays reported alongside urease experiments in the same publications (Figure 1b; Supplementary File, Tables S2 and S3). Manual review of 159 ambiguous PubChem assay identifiers established that 61 did not correspond to urease assays (Supplementary File, Table S3). Second, the initial filter based on reported activity had excluded 122 records explicitly classified as inactive and 114 classified as inconclusive, thereby discarding the only experimental evidence of inactivity available in the public datasets. Reinstating these records identified 98 molecules with at least one experimentally measured inactive result, providing a more complete representation of the available evidence concerning urease-inhibitor activity (Figure S1b; Supplementary File, Tables S2 and S3). Third, nine phosphinic acid inhibitors had been deposited with malformed simplified molecular-input line-entry system (SMILES) strings containing [P+](=O)O. Standardization converted this group into the invalid [PH+](=O)=O representation, silently producing missing descriptor values. Correction of the structures to the neutral pentavalent phosphinic acid increased descriptor completeness from 0.9926 to 1.000 (Supplementary File, Tables S2 and S3).

The corrected dataset contains 1218 molecules, of which 1110 have at least one direct quantitative concentration-response endpoint. Activity was expressed as pAct, defined as the negative base-10 logarithm of the reported molar IC_50_, K_i_, K_d_ or EC_50_ value, such that higher pAct values indicate greater apparent potency. Within this subset, the median molecule-level pAct was 4.72 (interquartile range 4.10–5.39; Figure 1c) and 178 molecules reached pAct >=6, corresponding to 16.0% of the molecules with a quantitative endpoint and 14.6% of the full dataset. UI-Ref should therefore be understood as a collection of compounds reported in urease assays rather than as a set of potent inhibitors. Inclusion indicates that a molecule was tested and reported, not that it exhibited sufficient potency to be of practical interest. This distinction is material because it determines whether dataset membership represents a biologically meaningful positive label or a record of what has been published.

Provenance within the set is highly structured. Of the 1218 molecules, 1142 carry a source-document identifier, distributed over 77 named documents. The eight largest named documents contribute 23.9% of the full set, and 20 publications account for half the documented molecules (Figure 1d; Supplementary File, Table S31). Source publication almost completely determines assay organism: the normalized conditional entropy H(organism|document)/H(organism) is 0.016, such that document accounts for 98.4% of the variation in organism, and only three of the 77 named documents report assays against more than one organism (Figure 1e; Supplementary File, Tables S3, S31 and S36). The corresponding values for scaffold and endpoint are 0.182 and 0.141, respectively, so document accounts for 81.8% of the scaffold variation and 85.9% of the endpoint variation. Supplementary File, Table S36, gives these statistics together with the counts on which they rest. Any validation scheme that partitions molecules at random will therefore train and test on the same congeneric series from the same publication.

### 2.2. An Empirical Reference Scale for Provenance-Associated Separability

**Figure 2:**
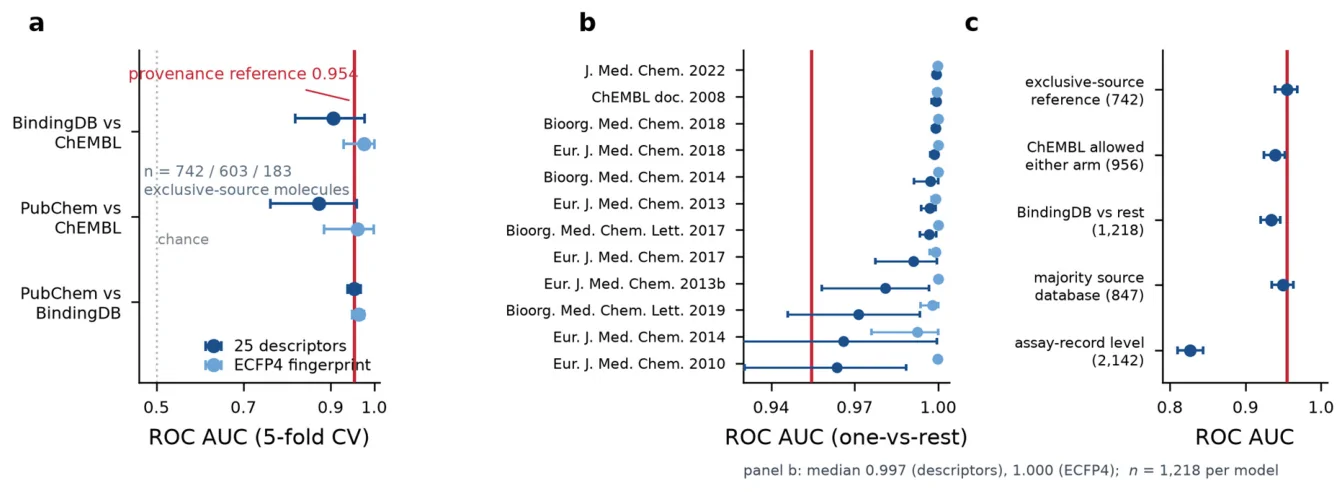
Prediction of molecular origin within UI-Ref, where every molecule is a compound reported in a urease assay. (**a**) Random forests separating pairs of source databases within UI-Ref under 5-fold stratified cross-validation with 2000-resample bootstrap intervals: PubChem versus BindingDB reaches AUC 0.954 [0.939–0.968] on 25 descriptors and 0.964 [0.950–0.976] on ECFP4 (n = 742 source-exclusive molecules); PubChem versus ChEMBL reaches 0.872 and 0.961 (n = 603), respectively, and BindingDB versus ChEMBL reaches 0.906 and 0.977 (n = 183), respectively. (**b**) One-versus-rest prediction of source document under 3-fold stratified cross-validation across the twelve largest documents in UI-Ref: median AUC 0.997 on descriptors and 1.000 on ECFP4. (**c**) Dependence of the estimate on the definition of the source label; AUC ranges from 0.827 at the record level to 0.954 among source-exclusive molecules. The dashed horizontal line marks the 0.954 reference value.

To quantify the provenance signal directly, molecular origin was predicted within UI-Ref, thereby holding reference-set membership constant (Supplementary File, Table S5). A random forest trained on 25 physicochemical descriptors distinguished molecules found exclusively in PubChem from those found exclusively in BindingDB with an AUC of 0.954 (95% CI 0.939–0.968; n = 742 source-exclusive molecules). Extended-connectivity fingerprints of diameter 4 (ECFP4s) increased the AUC to 0.964 (95% CI 0.950–0.976) (Figure 2a; Supplementary File, Table S5) [30,31,32]. A provenance signal persisted across definitions of source membership, with AUCs ranging from 0.827 at the individual-record level to 0.954 among source-exclusive molecules (Figure 2c; Supplementary File, Table S5). 

Source publication was even more predictable. In one-versus-rest models for the 12 largest contributing documents, the median AUC was 0.997 (range 0.962–1.000) using physicochemical descriptors alone and 1.000 using ECFP4 fingerprints (Figure 2b; Supplementary File, Table S5). Twenty-five basic molecular properties were therefore sufficient to discriminate source publications with near-perfect one-versus-rest AUCs. This result is consistent with the tendency of individual publications to report congeneric series with similar characteristics, such as molecular size, polarity and ring composition. Re-running the frozen scripts in the package versions given in Section 4.10 returns 0.9544 for the database-provenance value and 0.9934 for the median document-provenance value. The differences of +0.0004 and −0.0036 follow from the RDKit version and are listed, with every other such difference, in the repository. 

The database- and publication-provenance AUCs of 0.954 and 0.997, respectively, provide an empirical reference scale for interpreting the cross-library classifiers reported below, but they are not transferable thresholds. Source-label classification within UI-Ref and collection-membership classification between UI-Ref and an external library differ in terms of class priors, sample size, and validation structure, and the AUC does not transfer between tasks defined on different populations. This scale is therefore used as a diagnostic reference, and each cross-library classifier is also compared with null models constructed within the same task (Section 2.8).

### 2.3. Effect of Property Matching and Publication-Grouped Validation on Cross-Collection Separation

**Figure 3:**
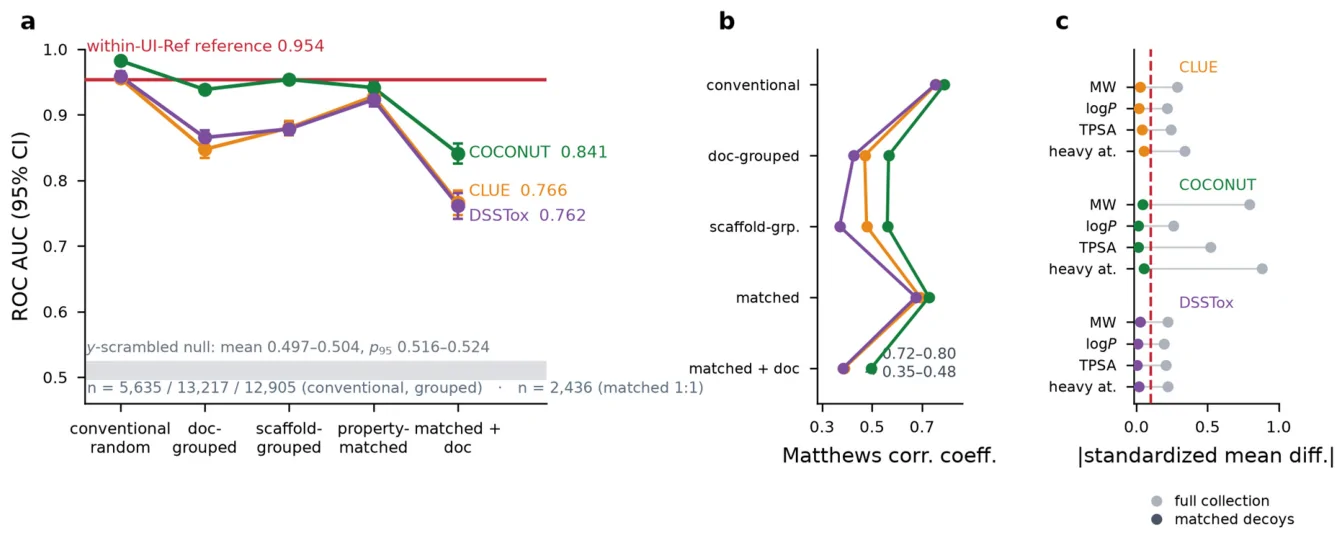
Cross-collection classification performance under five validation designs. (**a**) ROC AUC for the three comparisons, from left to right: conventional random cross-validation, document-grouped, scaffold-grouped, property-matched with random splits, and property-matched with document-grouped splits. UI-Ref versus CLUE decreases from 0.956 to 0.766 [0.747–0.785], versus COCONUT from 0.982 to 0.841 [0.826–0.856], and versus DSSTox from 0.959 to 0.762 [0.742–0.781]. MCC decreases in parallel, from 0.756 to 0.389, from 0.789 to 0.497 and from 0.753 to 0.385, respectively. For the unmatched designs, n = 5635, 13,217 and 12,905, and for the matched designs, n = 2436 (1218 UI-Ref molecules against 1218 property-matched comparison molecules in each case). The grey band denotes the y-scrambling null envelope (mean 0.497–0.504, 95th percentile 0.516–0.524 over the fifteen pairwise designs); the dashed line marks the 0.954 within-set provenance value from Figure 2, shown for orientation rather than as a decision threshold. (**b**) Matthews correlation coefficient for the same three comparisons across the same five designs, which decreases in parallel with the AUC. (**c**) Absolute standardized mean differences across the twelve matching covariates before and after nearest-neighbor matching on molecular weight, cLogP, TPSA and heavy-atom count. Balance across the full 25-descriptor block and the substructure indicators is reported in Figure S8 and Supplementary File, Table S35.

The conventional comparison was first reproduced, after which the corrections were introduced sequentially (Figure 3a–c; Supplementary File, Tables S6 and S7). The conventional design used a random forest with 300 trees, class-balanced weights, a stratified 70:30 split and the 25 physicochemical descriptors. Under this design, UI-Ref was readily separated from each external collection, with AUCs of 0.956 against CLUE, 0.982 against COCONUT and 0.959 against DSSTox (Figure 3a; Supplementary File, Table S6).

Grouping cross-validation folds by source document reduced the corresponding AUCs to 0.847, 0.939 and 0.865, respectively. Grouping by Bemis–Murcko scaffold produced AUCs of 0.880, 0.954 and 0.878, respectively (Figure 3a; Supplementary File, Table S6) [33]. The external collections were then replaced with property-matched negatives, obtained by 1:1 greedy nearest-neighbor matching without replacement on molecular weight, calculated logarithmic partition coefficient (cLogP), topological polar surface area (TPSA) and heavy-atom count, in accordance with established decoy-construction practice [17,19]. This design produced AUCs of 0.928, 0.941 and 0.923. Matching substantially improved covariate balance on the matching variables: the largest absolute standardized mean difference (SMD) decreased from 0.883 to 0.053, and all 12 values were below 0.1 after matching (Figure 3c; Supplementary File, Table S7). The balance on the full descriptor block and on substructure indicators is reported in Section 2.8.

Combining property matching with document-grouped validation produced the corrected estimates. The AUC was 0.766 (95% CI 0.747–0.785) against CLUE, 0.841 (0.826–0.856) against COCONUT and 0.762 (0.742–0.781) against DSSTox. Matthews correlation coefficients (MCCs) decreased correspondingly, from 0.756 to 0.389, from 0.789 to 0.497 and from 0.753 to 0.385, respectively (Figure 3b; Supplementary File, Table S6). Relative to the recomputed conventional design, the AUC decreased by 0.141–0.197, which shows that the estimated separation was sensitive to library composition and provenance and should not be interpreted solely as target-related chemistry.

All three corrected AUCs were below the within-set database-provenance value of 0.954 and well below the publication-provenance value of 0.997. None of the cross-library comparisons therefore separated UI-Ref from the external collections as accurately as molecular representations predicted the source database or publication within UI-Ref. The two tasks are defined on different populations, so this ordering is a descriptive observation and not a test; Section 2.8 gives the corresponding task-matched comparisons. Interpretation changed the most for DSSTox, which ranked among the strongest separations under the conventional design and produced the lowest fully corrected AUC, 0.762. The data give little support for a strong chemical distinction between reported urease inhibitors and DSSTox compounds, but they do not establish that such a distinction is absent.

A reproducible separation signal nevertheless remained. Across 100 label permutations for each of 17 designs, the mean null AUC ranged from 0.497 to 0.504 (standard deviation 0.010 to 0.031). For the 15 pairwise designs, the 95th percentile of the null ranged from 0.516 to 0.524, increasing to 0.560–0.603 for the two provenance designs, which contained small minority classes (Figure S3b; Supplementary File, Table S8). Every observed AUC exceeded the corresponding 95th percentile of its null, with a permutation *p*-value of 0.0099 in every design [34]. These results indicate a residual difference between UI-Ref and the external collections beyond that expected under label permutation. Its magnitude is considerably smaller than that suggested by the conventional analysis, and, as shown in Section 2.8, the design cannot distinguish target-related chemistry from residual provenance structure. 

### 2.4. Descriptor Attributions Partly Reflect Provenance

Analysis by Shapley additive explanations (SHAP) of the conventional models reproduced the descriptor ranking commonly reported in cross-collection comparisons (Figure S4a; Supplementary File, Table S10). The fraction of sp3-hybridized carbon atoms (FractionCSP3) ranked first or second across the six collection models and accounted for 12.9–21.8% of the total mean absolute SHAP attribution, followed by descriptors related to aromatic-ring and heteroatom content [35,36]. Rotatable-bond count, although frequently emphasized in interpretations of such models, was not a leading descriptor: it ranked between fourth and sixteenth and accounted for 2.7–6.6% of the total attribution.

Whether these attribution profiles reflect urease-related chemistry or differences associated with molecular provenance was examined next. Descriptor rankings from each collection model were compared with those of the provenance classifiers. The DSSTox model showed a significant rank correlation with the database-provenance classifier (Spearman rho = 0.422, *p* = 0.036), which increased when compared with the document-membership classifier (rho = 0.533, *p* = 0.006). The CLUE and COCONUT models showed weaker, non-significant correlations, with rho between 0.05 and 0.33 (Figure S4b; Supplementary File, Table S11). Correcting the design had comparatively little effect on descriptor ordering: Spearman correlations between the conventional and the matched, document-grouped rankings were 0.81 for CLUE, 0.64 for COCONUT and 0.83 for DSSTox (Figure S4c; Supplementary File, Tables S10 and S11).

The provenance classifier itself relied primarily on cLogP and molar refractivity. FractionCSP3 fell to rank six and accounted for only 5.0–6.5% of its total attribution. The SHAP profiles of the collection models therefore cannot be explained entirely by provenance, but neither can they be interpreted as direct evidence of pharmacologically relevant features. At least part of the attributed importance reflects structural differences associated with where molecules were deposited or published, so pharmacological interpretation requires an explicit provenance control (Figure S4a–c; Supplementary File, Tables S10 and S11).

### 2.5. Chemical-Space Overlap Depends on Sample Size and Projection

**Figure 4:**
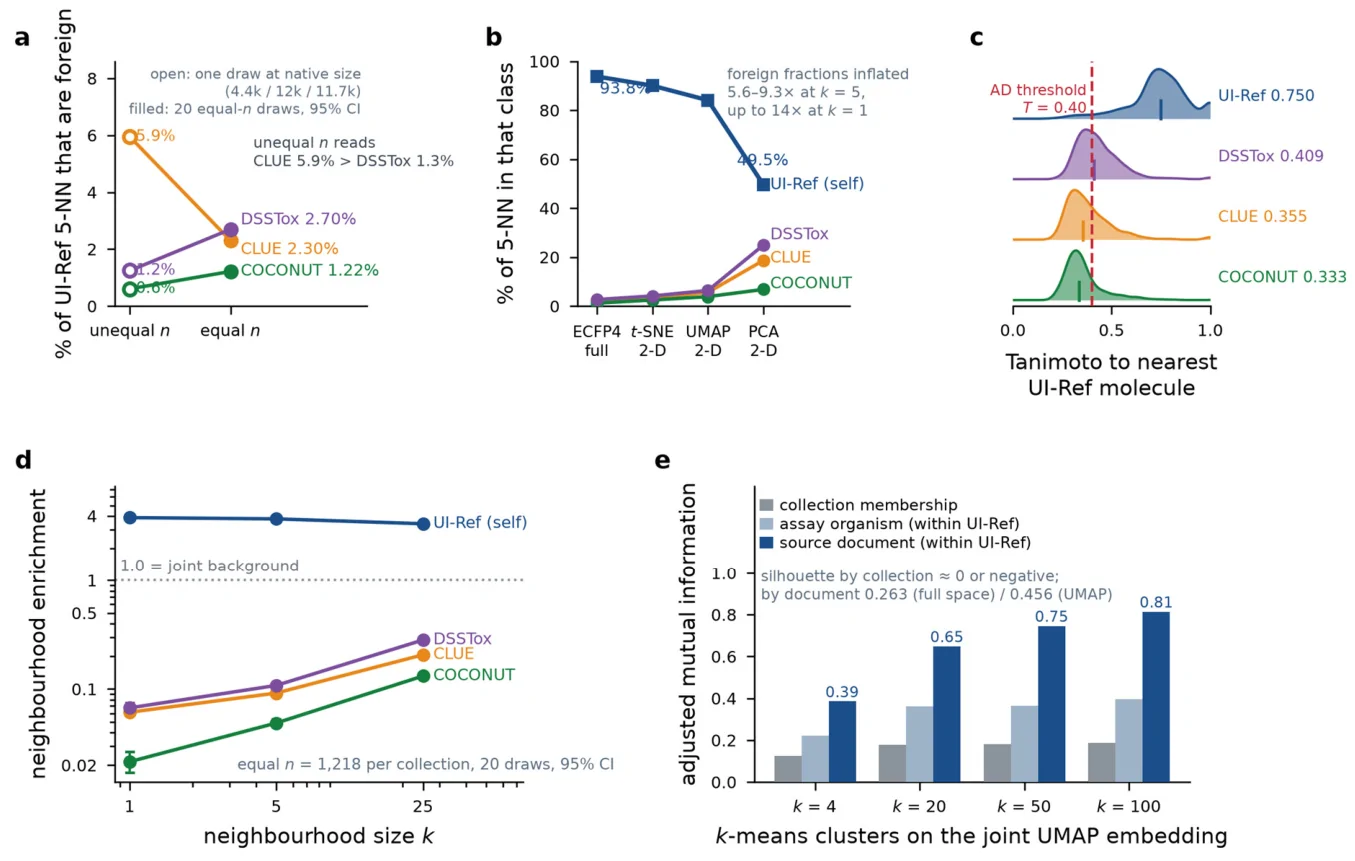
Dependence of chemical-space overlap on sample size and projection. (**a**) Fraction of the five nearest ECFP4 neighbors (5-NN) of a UI-Ref molecule drawn from each external collection. When whole collections of unequal size are compared, CLUE appears closest (5.9%) and DSSTox most distant (1.2%); resampling every collection to n = 1218 over 20 independent draws reverses the order (DSSTox 2.70%, CLUE 2.30%, COCONUT 1.22%). (**b**) The same statistic computed in the full 2048-bit ECFP4 space and in a two-dimensional PCA of the ECFP4 fingerprints: 93.8% of the 5-NN of a UI-Ref molecule are UI-Ref molecules in full space against 49.5% in the PCA map, and foreign fractions increase by factors of 5.6–9.3 at k = 5 and up to 14 at k = 1 (DSSTox). (**c**) Distribution of the nearest-neighbor ECFP4 Tanimoto similarity from each UI-Ref molecule to the collection indicated. Median values are 0.750 for UI-Ref (leave-one-out), 0.409 for DSSTox, 0.355 for CLUE and 0.333 for COCONUT; 2.4% of UI-Ref molecules have a CLUE neighbor at Tanimoto >= 0.7. (**d**) Neighborhood enrichment relative to a random-composition expectation: all foreign collections are depleted (0.022–0.282), whereas UI-Ref is self-enriched 3.38- to 3.85-fold. (**e**) AMI between Butina clusters and three candidate labels. AMI is 0.125–0.187 with collection, 0.385–0.814 with UI-Ref source document and 0.220–0.396 with assay organism; the silhouette coefficient by collection is approximately zero or negative, whereas the silhouette by document within UI-Ref is 0.263 in full space and 0.456 in the UMAP embedding. Throughout, n = 1218 UI-Ref molecules.

The conventional interpretation that urease inhibitors overlap preferentially with the drug collection and remain separate from the toxicological collection rests largely on low-dimensional projections and nearest-neighbor composition. Both quantities depend strongly on how the analysis is constructed (Figure 4; Supplementary File, Tables S12–S15).

Under the original unbalanced sampling scheme, UI-Ref and CLUE were retained in full, whereas COCONUT and DSSTox were reduced by diversity-based subsampling. In that setting, 5.9% of the five nearest neighbors of a UI-Ref molecule belonged to CLUE, compared with 1.2% from DSSTox, a difference that could be read as preferential overlap with drug-like molecules. After equalization of all four collections to 1218 molecules across 20 independent draws, however, the ordering changed: DSSTox accounted for 2.70% of neighbors (standard deviation 0.14%), CLUE for 2.30% (0.12%) and COCONUT for 1.22% (0.09%) (Figure 4a; Supplementary File, Table S12). Relative to the proportions expected from collection size, all three external collections were depleted, with enrichment values between 0.022 and 0.282, whereas UI-Ref was enriched 3.38- to 3.85-fold in its own neighborhoods (Figure 4d; Supplementary File, Table S12).

Dimensionality reduction introduced a further distortion. In the complete 2048-bit fingerprint space, 93.8% of the five nearest neighbors of a UI-Ref molecule were other UI-Ref molecules. After projection by principal component analysis (PCA), this fraction fell to 49.5%, while the apparent contributions of the external collections increased by factors of 5.6 to 9.3, reaching 14-fold for DSSTox at k = 1. t-distributed stochastic neighbor embedding (t-SNE) and uniform manifold approximation and projection (UMAP) preserved the original neighborhood structure more closely, retaining UI-Ref fractions of 90.0% and 84.1%, respectively (Figure 4b; Supplementary File, Table S12). The first two principal components accounted for only 3.5% and 2.9% of the variance in the joint fingerprint space. Neighborhood composition measured on this projection therefore describes the geometry of the two-dimensional map rather than the geometry of the original molecular representation.

The absolute similarities likewise provide little evidence of close overlap between collections. The median Tanimoto similarity from a UI-Ref molecule to its nearest neighbor was 0.750 within UI-Ref, compared with 0.355 for CLUE, 0.409 for DSSTox and 0.333 for COCONUT. Only 2.4% of UI-Ref molecules had a CLUE nearest neighbor with a Tanimoto similarity of at least 0.7 (Figure 4c; Supplementary File, Table S13). A median similarity of 0.355 indicates weak fingerprint similarity and does not support the interpretation that the collections contain close structural analogues. Describing them as occupying the same chemical space therefore overstates the degree of molecular overlap observed.

The dominant grouping signal within UI-Ref was instead associated with source publication. The adjusted mutual information (AMI) between k-means clusters and collection membership ranged from 0.125 to 0.187, compared with 0.385–0.814 for source document and 0.220–0.396 for assay organism. Silhouette coefficients based on collection labels were close to zero or negative, whereas document-based grouping within UI-Ref reached a silhouette coefficient of 0.263 in the complete fingerprint space and 0.456 in the UMAP embedding (Figure 4e; Supplementary File, Table S15) [37]. Much of the apparent organization of this chemical space is therefore associated with the bibliographic origin of the molecules rather than with collection identity alone.

### 2.6. Structural Alerts and Chemotype–Potency Associations Are Document-Bound

**Figure 5:**
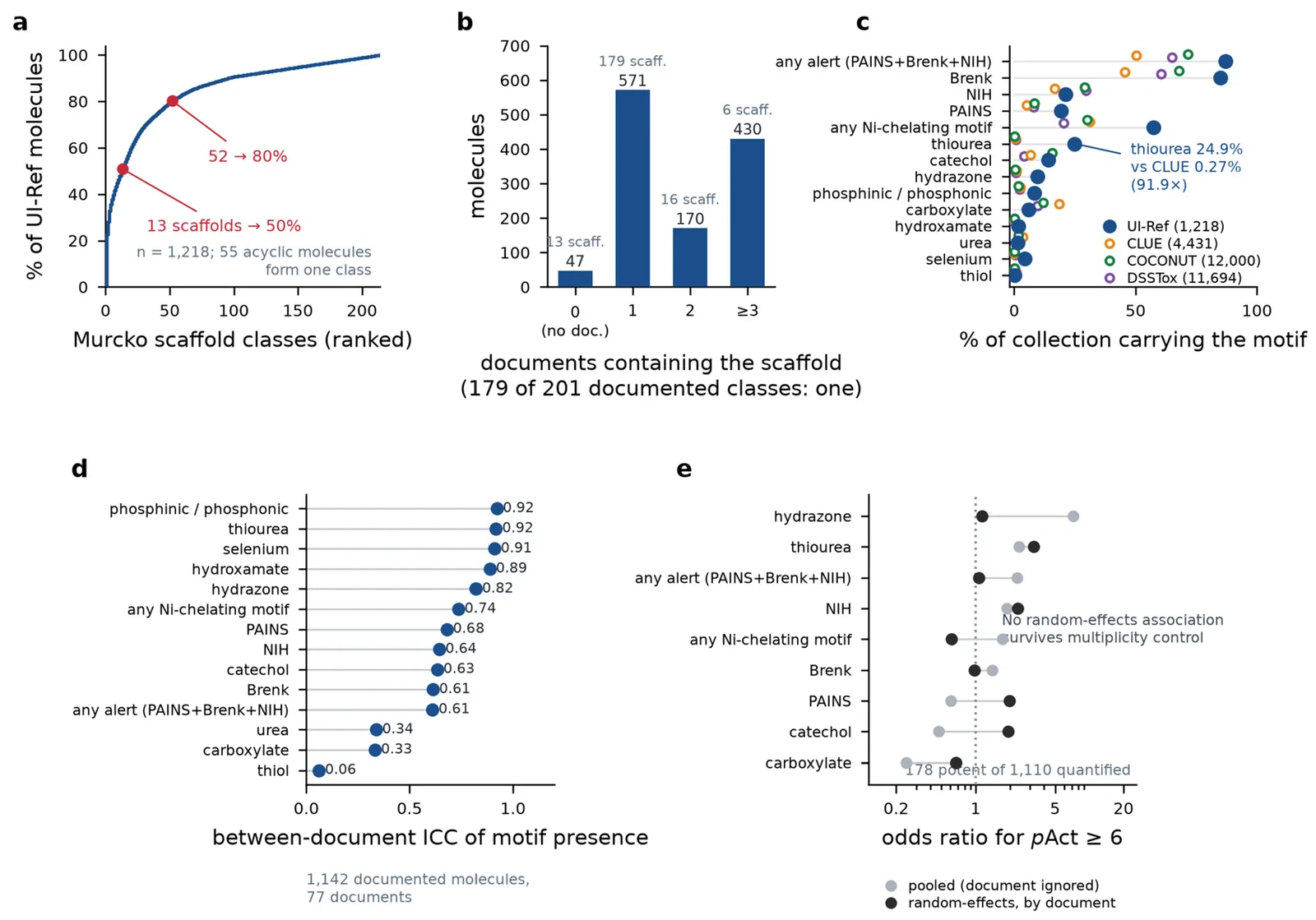
Scaffold concentration, structural-alert burden and document dependence of motif–potency associations in UI-Ref. (**a**) Cumulative molecule coverage over the 214 Bemis–Murcko scaffold classes: 13 classes cover 50%, and 52 cover 80%. (**b**) Scaffold classes by the number of distinct source documents in which they occur. Of the 201 classes represented among documented molecules, 179 occur in exactly one document; 13 additional classes have no source-document assignment. (**c**) Structural-alert and metal-chelator prevalence in UI-Ref and the three external collections. (**d**) Between-document intraclass correlation for each motif. (**e**) Pooled odds ratios compared with document-level random-effects estimates for motifs supported by at least three informative document strata. Alert prevalences and intraclass correlations use the 1142 documented molecules over 77 documents; the odds ratio analysis uses the 1110 molecules with a quantitative pAct, of which 178 are potent (pAct >= 6). All 11 SMARTS controls pass (Supplementary File, Tables S17 and S33).

Scaffold diversity in UI-Ref is limited and strongly associated with source publication. The 1218 molecules comprise 214 Bemis–Murcko scaffold classes, including a single acyclic class containing 55 molecules. Thirteen scaffold classes are sufficient to cover 50% of the dataset, and 52 cover 80% (Figure 5a; Supplementary File, Tables S16 and S22). When undocumented molecules are assigned singleton provenance groups, 185 of the 214 classes occur in exactly one group; among the 201 classes represented by documented molecules, 179 occur in exactly one source document. Most scaffolds therefore represent individual congeneric series reported by a single research group rather than independently sampled regions of chemical space (Figure 5b; Supplementary File, Tables S16 and S36).

UI-Ref also contains a high prevalence of structural alerts and metal-chelating motifs. Brenk alerts occur in 84.8% of molecules, alerts from the National Institutes of Health (NIH) filter set in 21.2%, pan-assay interference compounds (PAINS) in 19.2%, and at least one nickel-chelating motif in 57.3% [38,39]. Thiourea, an established urease-inhibitor chemotype, occurs in 24.88% of UI-Ref but in only 0.27% of CLUE, a 91.9-fold difference (Figure 5c; Supplementary File, Table S18). All 11 positive and negative controls for the SMARTS (SMILES arbitrary target specification) patterns produced the specified result (Supplementary File, Tables S17 and S22). The patterns are frozen in the released analysis package and are revalidated against these 11 controls on every run; the prevalences they give differ from the counts of Table S21 by −11 to +1 molecules across the nine motifs and by −20 for the any-chelator indicator (Supplementary File, Table S33).

These motifs are not distributed independently across the dataset. Among the 48 source documents contributing at least 10 molecules, 84% of the 672 document-by-motif combinations had a prevalence below 5% or above 95%; that is, a motif was usually either nearly absent from a publication or present in almost every molecule that publication reported (Figure S6a; Supplementary File, Table S19). These 48 documents contributed 1020 of the 1142 molecules with an assigned source document and varied substantially in size (Figure S6b; Supplementary File, Tables S19 and S31). Consistent with this concentration, the between-document intraclass correlation coefficient (ICC) reached 0.92 for the phosphinic/phosphonic and thiourea motifs and 0.91 for selenium (Figure 5d; Supplementary File, Table S20).

The apparent relationships between chemotype and potency changed substantially once document structure was taken into account. Pooled analyses suggested strong associations for selenium, hydrazone and thiourea, whereas document-aware estimates were supported by few informative strata. In the hierarchical reanalysis, selenium had only one informative document stratum and was therefore not interpreted directionally. None of the 12 estimable random-effects associations survived Benjamini–Hochberg correction at a false discovery rate of 0.05 or 0.10 (Figure 5e; Supplementary File, Table S37). The pooled associations therefore reflect, to a substantial extent, which laboratories studied which chemotypes and should not be interpreted as document-independent relationships between structural motifs and urease-inhibitor potency.

### 2.7. Practical Limits of Prediction from the Current Urease Record

Five candidate prediction tasks were evaluated using UI-Ref and the external collections. Table 2 defines the question posed by each task, the experimental meaning of its positive and negative classes, the validation strategy applied and the conclusions that its output can support. Tasks based on source documents use document-grouped cross-validation, whereas tasks involving collections without publication identifiers use Bemis–Murcko scaffold grouping.

To establish what can be predicted reliably from the available urease data, five questions that commonly arise in compound prioritization were evaluated (Table 2; Supplementary File, Table S23). Only two have labels grounded directly in experimental measurements. The first distinguishes molecules reported as active from those explicitly measured as inactive. The second distinguishes potent from weak compounds at a threshold of pAct >= 6. A third task separates UI-Ref molecules from property-matched, untested compounds drawn from external collections; because these external molecules have not necessarily been assayed against urease, this task can support compound ranking or enrichment but cannot estimate the probability of urease inhibition.

The remaining two questions are not valid biochemical prediction tasks. Structural-alert status is calculated deterministically from SMARTS patterns, so fitting a model to reproduce it adds no information. Similarly, distinguishing launched from non-launched compounds within CLUE measures similarity to categories in a clinical development library and does not estimate the probability of approval or of clinical success. Both analyses were retained as diagnostic comparisons because they demonstrate how apparently strong model performance can arise from labels that do not answer the intended scientific question (Table 2; Supplementary File, Table S23).

**Figure 6:**
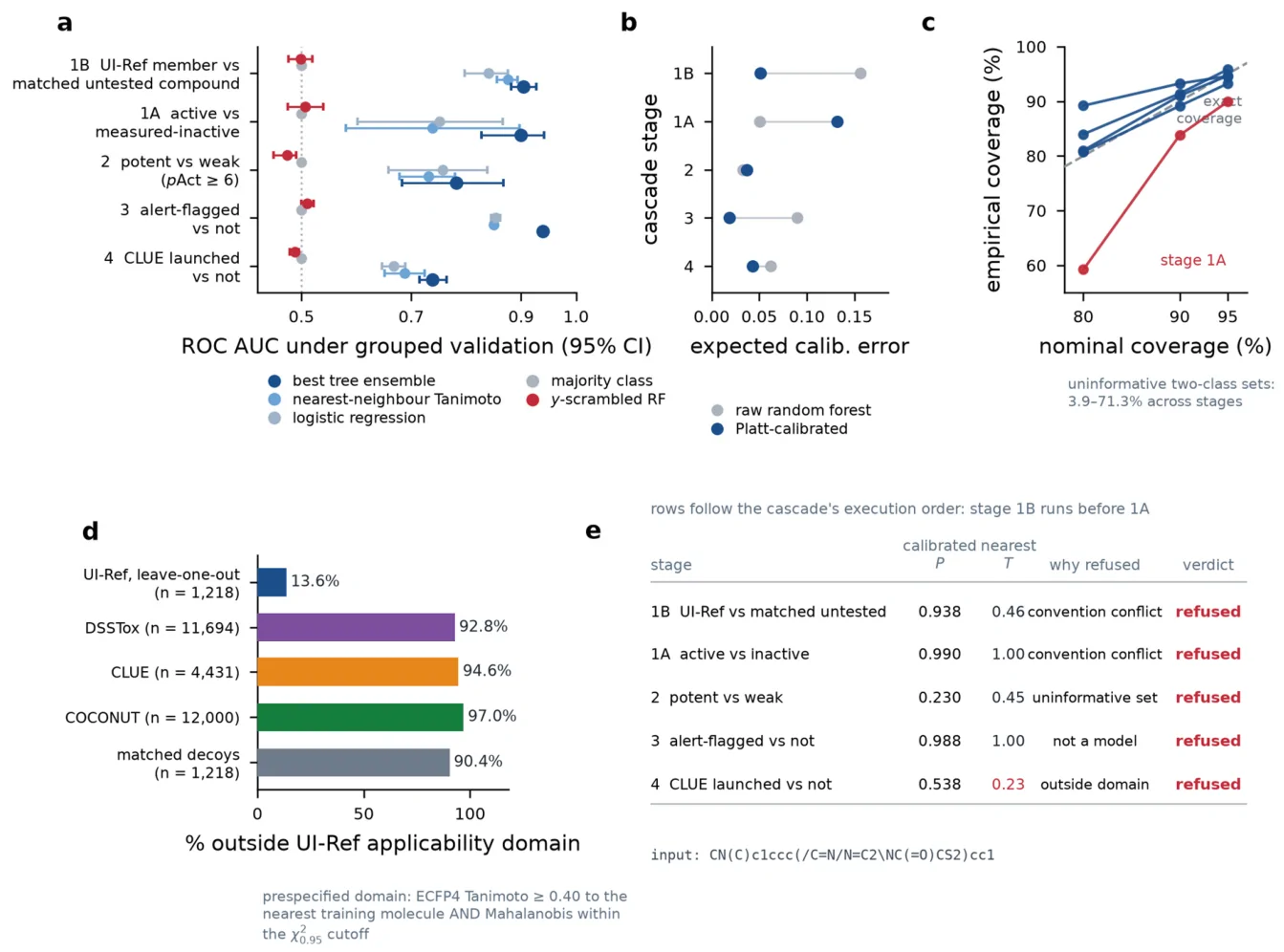
Performance, uncertainty and applicability of prediction tasks based on UI-Ref. (**a**) ROC AUC of the best-performing tree ensemble compared with nearest-neighbor, logistic-regression, majority-class and label-permutation baselines. Validation was grouped by source document for Stages 1A, 1B and 2 and by Bemis–Murcko scaffold for Stages 3 and 4. Stage 1A is shown for comparison only: it is reported as a narrow-scope diagnostic and is excluded from the general predictive cascade (Section 2.7 and Section 2.11). (**b**) Reliability before and after probability calibration. (**c**) Empirical coverage of Mondrian conformal prediction at alpha = 0.05, 0.10 and 0.20. (**d**) Fraction of each collection outside the applicability domain, defined jointly by an ECFP4 nearest-neighbor Tanimoto similarity of at least 0.40 and a Mahalanobis-distance criterion. The out-of-domain fractions were 94.6% for CLUE, 97.0% for COCONUT, 92.8% for DSSTox and 90.4% for matched decoys, compared with 13.6% for UI-Ref under leave-one-out evaluation. (**e**) Worked example showing how tautomer standardization affects molecular identity and applicability-domain assessment. Under the declared standardization workflow, the compound maps to UIREF-1160, a molecule with a measured urease-active record and an IC_50_ of 7.3 µM. The alternative tautomer representation produces a different identity and domain assessment. Predictive stages abstain when standardization, conformal or applicability-domain criteria do not support a unique verdict; structural alerts are returned directly from SMARTS matching.

Performance under grouped validation depended strongly on label definition and empirical basis (Figure 6a; Supplementary File, Table S24). The potency task used quantitative measurements for both classes; its best model reached an AUC of 0.782 (95% CI 0.683–0.867) and an MCC of 0.504 under document-grouped cross-validation, compared with an AUC of 0.903 under random splitting. The measured-active-versus-measured-inactive task reached an AUC of 0.879 (95% CI 0.823–0.922) on fingerprints, with an MCC of 0.318 (0.191–0.445). The primary limitation of this stage is the composition of its negative class: 59 unambiguously inactive molecules from six publications, six organism labels, and 14 scaffold classes. Within the six documents containing both classes, the descriptor model attained an AUC of 0.393 (0.270–0.520), with a point estimate below 0.5 but a confidence interval that included 0.5, and an MCC of −0.267. Aggregate performance at this stage is therefore more consistent with recognition of publication origin than with discrimination of measured activity, and the stage is reported as a narrow-scope diagnostic rather than as a general predictive stage of the cascade. This finding also places our previous urease-classification study in clearer context. In that work, several bioactivity cutoffs were compared, and two-cutoff strategies that retained an explicit indeterminate zone for compounds with intermediate IC_50_ values were found to be preferable to forcing every molecule into a binary class [40]. The present results support that approach while also delimiting it: an indeterminate zone reduces ambiguity in the assignment of potency labels, but it cannot compensate for the scarcity of experimentally measured inactive compounds. The bioactivity cutoffs used to define the classes should furthermore not be conflated with the probability threshold used to convert model scores into predictions. Urease classifiers should therefore be evaluated across plausible bioactivity cutoffs, permit an indeterminate outcome for borderline compounds, and report threshold-dependent metrics under the observed class distribution.

Detailed comparisons of algorithms and molecular representations are provided for ranking against matched untested compounds (Figure S2a), measured activity (Figure S2b), potency (Figure S2c), structural-alert status (Figure S2d) and CLUE development status (Figure S2e; Supplementary File, Table S24). The 25 physicochemical descriptors used in these comparisons are defined in Supplementary File, Table S30. The results for structural alerts and CLUE status are included for diagnostic purposes and should not be interpreted as evidence of toxicity or of clinical predictability.

Near-duplicate leakage accounts for part of the difference between random and grouped validation. Under random splitting, approximately 64% of UI-Ref test molecules had a training-set neighbor with an ECFP4 Tanimoto similarity of at least 0.7. This fraction decreased to approximately 18% after grouping by source document and to 15% after grouping by scaffold (Figure S5a; Supplementary File, Table S9). Grouped designs also showed a lower median maximum similarity and fewer nearly identical training–test pairs at Tanimoto >= 0.9 (Figure S5b; Supplementary File, Table S9). Random validation therefore measures, in part, the ability to recognize close analogues from the same published series.

Calibration improved the numerical reliability of some outputs but did not alter what their labels represented. Platt scaling reduced the expected calibration error (ECE) from 0.156 to 0.051 for the ranking task based on matched untested compounds. No calibration method improved on the raw model for the much smaller measured-inactive task (Figure 6b; Supplementary File, Table S25) [41]. Calibration also improved the numerical fit of the structural-alert classifier, but this does not render the task scientifically predictive, because the correct alert status is obtainable directly from the SMARTS rules.

Conformal prediction provided a more explicit representation of uncertainty. At 90% nominal coverage, empirical coverage ranged from 0.838 to 0.914 across the five candidate tasks. Coverage was close to nominal for four tasks but fell short for the measured-active-against-measured-inactive comparison, for which only 59 inactive molecules were available for grouped calibration and testing (Figure 6c; Figure S7a; Supplementary File, Table S27). At the same nominal coverage, between 19.7% and 57.1% of prediction sets contained both classes, which indicates that the data did not support a unique classification for those molecules. For the potency task, this fraction reached 66.2% at 95% nominal coverage (Figure S7b; Supplementary File, Table S27) [26]. Permitting more singleton predictions by increasing the miscoverage level generally reduced their accuracy (Figure S7c; Supplementary File, Table S27). The conformal analysis therefore makes the trade-off between coverage, decisiveness and accuracy explicit. Coverage validity requires exchangeability between the calibration and test sets, an assumption that is examined per stage in Section 2.11.

The applicability domain imposed the strongest practical restriction. A molecule was considered to be inside the prespecified domain only if it had an ECFP4 Tanimoto similarity of at least 0.40 to the nearest training molecule and a Mahalanobis distance within the prespecified chi-square cutoff. Under these criteria, 94.6% of CLUE, 97.0% of COCONUT, 92.8% of DSSTox and 90.4% of the property-matched compounds lay outside the UI-Ref domain, compared with 13.6% of UI-Ref under leave-one-out evaluation (Figure 6d; Supplementary File, Table S26). Models trained on the current public urease record therefore have limited support for extrapolation to broad screening libraries. The dependence of these fractions on the chosen thresholds and molecular representation is quantified in Section 2.11.

Applying the domain and uncertainty criteria sequentially further reduced the number of supported predictions. In a cohort of 244 molecules, 25 (10.3%) retained an in-domain, single-class prediction across the labeled tasks included in the joint evaluation. The estimated joint accuracy among these 25 molecules was 0.84 (95% CI 0.680–0.960; Supplementary File, Table S28). The low retention rate reflects the restrictive nature of the uncertainty analysis; it indicates that the available urease data support confident predictions only within a restricted region of chemical space.

A worked example illustrates why molecular standardization must be included in this assessment. The query compound was a 4-(dimethylamino)benzaldehyde-derived thiazolidinone hydrazone, represented as CN(C)c1ccc(/C=N/N=C2\NC(=O)CS2)cc1. Under the standardization procedure used to construct UI-Ref, it canonicalizes to the hydroxythiazole tautomer CN(C)c1ccc(/C=N/Nc2nc(O)cs2)cc1, with InChIKey YXXKRHZFVGZYRJ-NTUHNPAUSA-N. This standardized structure is already present in UI-Ref as UIREF-1160, a measured inhibitor with an IC_50_ of 7.3 microM against Sporosarcina pasteurii (BindingDB [42]; Supplementary File, Table S32). Retaining the input tautomer instead produces a different InChIKey, AOFVYOCIKCHIBD-NTUHNPAUSA-N, and causes the compound to be treated as previously unseen. For the measured-activity task, the nearest-neighbor Tanimoto similarity changes from 1.00 under the UI-Ref standardization convention to 0.34 under the alternative convention. The same input can therefore be classified either as an existing training compound or as an out-of-domain query, depending only on how tautomer identity is handled (Figure 6e; Supplementary File, Tables S4 and S29).

The decision framework does not return a single inhibitor verdict for this molecule. The measured-activity and matched-compound tasks are declined because the two standardization conventions produce conflicting applicability-domain results. The potency prediction is uninformative because its conformal set contains both classes. Structural-alert status is returned directly from the SMARTS evaluation, which identifies three alerts, rather than from a fitted classifier. The clinical-library comparison lies outside the applicability domain under both standardization conventions, with nearest-neighbor Tanimoto similarities of 0.228 and 0.222 (Figure 6e; Supplementary File, Table S29).

The calibrated probabilities span 0.230–0.990 across these questions, but they do not refer to a common endpoint. A molecule may be experimentally active but weak, resemble compounds previously tested against urease, and remain unrelated to launched drugs. These are distinct statements. For models built from the current urease record to be useful, every output must therefore remain linked to its experimental label, validation unit, standardization convention, conformal prediction set and applicability domain.

### 2.8. Task-Matched Null Models and Covariate Balance

**Figure 7:**
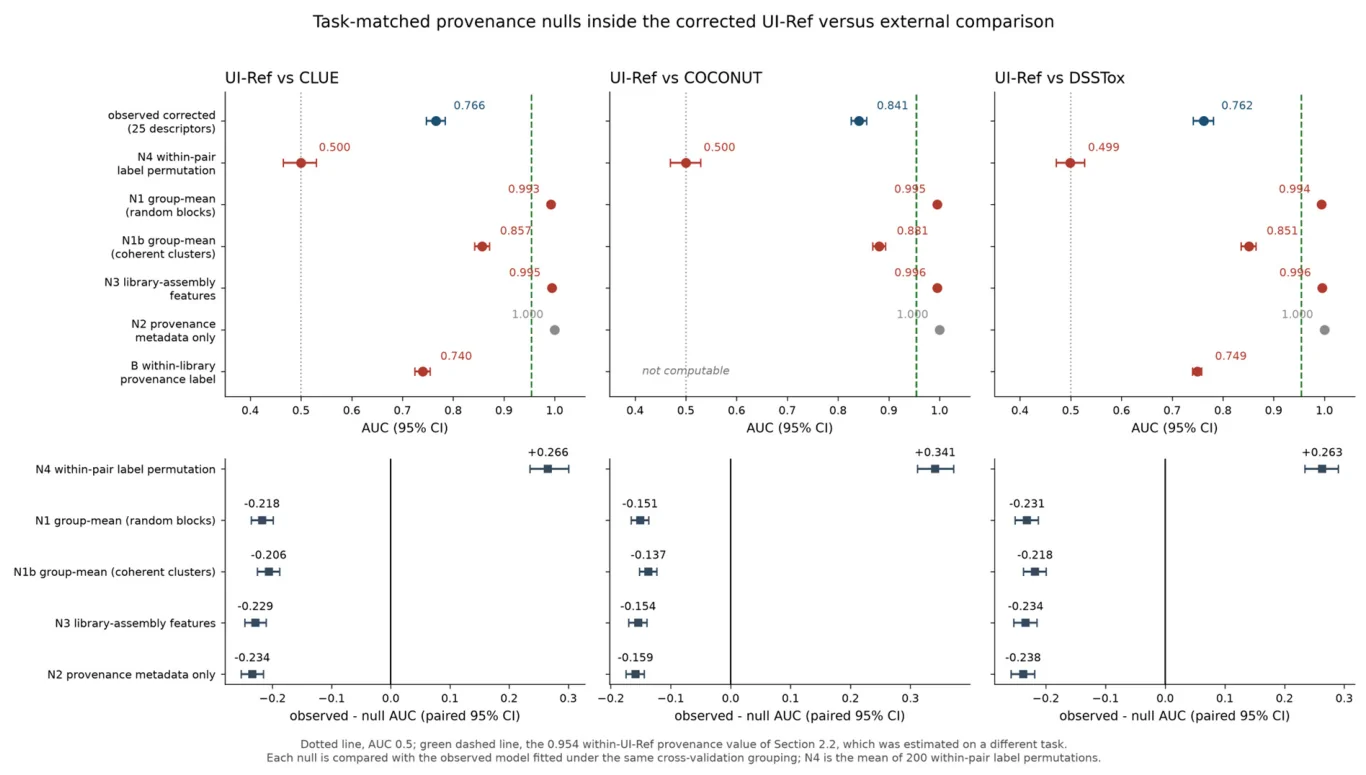
Task-matched null models inside the corrected UI-Ref-against-external comparison. Upper row: AUC with 95% confidence intervals for the observed model and for each null, per collection. Each null is fitted with the same estimator, document-grouped folds and seed as the observed model. N4 is the mean of 200 within-matched-pair label permutations; N1 and N1b replace each molecule’s descriptors with the mean descriptor vector of its provenance block, using random and chemically coherent external blocks respectively; N3 uses five library-assembly features (own-library scaffold frequency and fingerprint-neighborhood density) that encode relative library position rather than absolute physicochemical descriptor values; N2 uses provenance metadata only and is degenerate by construction, because metadata availability is itself a perfect provenance marker (AUC 1.000, shown in grey and carrying no inferential weight); and B predicts a provenance label inside the external library itself and is not computable for COCONUT, which releases no provenance field. The dotted line marks AUC 0.5 and the dashed line the 0.954 within-UI-Ref value of Section 2.2, which was estimated on a different task and is shown for orientation only. Lower row: paired differences between the observed model and each null, resampling the same test molecules for both models over 2000 bootstrap replicates. In every paired comparison, n = 2436 test molecules; the within-library baseline B is fitted inside the external library itself (n = 4336 for CLUE and 11,694 for DSSTox).

The values of 0.954 and 0.997 reported in Section 2.2 were estimated on source-label classification within UI-Ref, whereas the cross-library estimates of Section 2.3 concern collection membership between UI-Ref and an external library. The two tasks differ in class prior, sample size, class composition and validation grouping, and the AUC is not transferable between tasks defined on different populations. Four null models were therefore constructed inside the corrected UI-Ref-against-collection design itself, using the same estimator, the same document-grouped folds and the same seed, such that the residual separation is compared against a quantity estimated on the same molecules (Figure 7; Supplementary File, Table S34).

The most informative of these is a library-assembly null, which replaces every molecular descriptor with five features that describe the molecule’s relative position within its source library, namely, the frequency of its scaffold in that library and the density of its fingerprint neighborhood there; the null therefore does not encode absolute physicochemical descriptor values. Under the corrected design, this null reached an AUC of 0.9947 (95% CI 0.9924–0.9966) against CLUE, 0.9956 (0.9934–0.9972) against COCONUT and 0.9957 (0.9936–0.9974) against DSSTox, on n = 2436 molecules in each comparison. The corresponding observed models, fitted on all 25 descriptors, reached 0.766, 0.841 and 0.762, respectively. Paired bootstrap resampling of the same test molecules for both models gave differences of −0.229 (95% CI −0.247 to −0.210), −0.154 (−0.170 to −0.139) and −0.234 (−0.254 to −0.215), respectively, with bootstrap *p* < 0.001 in each case, and the DeLong test for correlated receiver operating characteristic curves gave Z = −24.25, −20.13 and −24.58, respectively (*p* < 1 × 10^−16^). A group-mean-descriptor null, in which each molecule is represented by the mean descriptor vector of its provenance block rather than by its own descriptors, behaved similarly (AUC 0.993, 0.995 and 0.995 with random blocks; 0.857, 0.881 and 0.851 when the external blocks were made chemically coherent). Information about library assembly alone therefore yielded higher AUCs than the full 25-descriptor representation.

Two additional null models characterize a label-permutation baseline and within-library provenance predictability. Permuting collection labels within matched pairs while preserving the matched-pair structure gave mean AUCs of 0.5002 (SD 0.0166) against CLUE, 0.5001 (0.0157) against COCONUT, and 0.4989 (0.0145) against DSSTox over 200 replicates. The observed models exceeded this null by 0.266 (95% CI 0.235–0.301), 0.341 (0.312–0.372), and 0.263 (0.234–0.290), respectively, with a permutation *p*-value of 0.005, the smallest value attainable with 200 replicates. The corrected design therefore captures a detectable difference between collections. Predicting a provenance label inside each external library using the same 25 descriptors and the same estimator gave an AUC of 0.7397 (0.7249–0.7544) for CLUE development status (n = 4336) and 0.7494 (0.7403–0.7579) for the DSSTox registration block, a purely administrative property of the identifier (n = 11,694). Both values are close to, but below, the 0.762–0.841 range obtained for cross-collection membership. This similarity indicates that the descriptor-based predictability of an administrative label can approach that of collection membership; it does not establish equivalence between the tasks. COCONUT releases no provenance field, so the equivalent estimate could not be computed for that library.

Treating provenance predictability as a covariate rather than as a threshold led to the same interpretation. Stratifying the observed comparison by decile of the library-assembly score left 1.26%, 1.21%, and 0.95% of positive–negative pairs comparable for CLUE, COCONUT, and DSSTox, respectively, with conditional AUCs of 0.632, 0.742, and 0.666 on those pairs, respectively. Thus, 98.7–99.1% of the corrected discrimination occurred between molecules already separated by provenance-derived information. The residual separation is detectable, but the design cannot distinguish target-related chemistry from residual provenance structure.

Covariate balance was then examined across everything the models can use rather than across the matching variables alone. On the four matching covariates, the published matching performs as reported, with a maximum absolute SMD of 0.059 across the three collections. Of the 39 covariates visible to the models, however, 27 for CLUE, 25 for COCONUT and 26 for DSSTox remain above an absolute SMD of 0.1 after matching, with maxima of 0.811 and 1.104 for FractionCSP3 and 0.753 for thiourea; considering the 25 descriptors alone, 14, 15 and 13 remain above 0.1, respectively (Supplementary File, Table S35; Figure S8). Imbalance persists specifically in urease-relevant chemistry: the SMD for thiourea is 0.800, 0.796 and 0.753 after matching, against 0.799, 0.801 and 0.773 before, and for the presence of any chelating motif, it is 0.539, 0.500 and 0.745 against 0.554, 0.573 and 0.845. Matching on four physicochemical properties therefore balances those four properties and leaves the substructure composition of the two classes substantially different. Describing the matched comparison as balanced is therefore not warranted.

Four alternative balancing schemes were applied to the full 25-descriptor block under the same document-grouped validation: entropy balancing, coarsened exact matching, propensity-score weighting for the average treatment effect on the treated, and 1:1 matching on the full 25-descriptor Mahalanobis distance. Entropy balancing satisfied its moment conditions to within 1 × 10^−16^; standard deviations and reduced the maximum absolute SMD over the 25 descriptors to 0.000, at the cost of an effective control sample of 335 of 4431 for CLUE, 63 of 12,000 for COCONUT and 1102 of 11,694 for DSSTox; the COCONUT estimate is consequently imprecise. Across the five schemes, the corrected AUC ranged from 0.670 to 0.781 for CLUE, from 0.690 to 0.879 for COCONUT and from 0.700 to 0.803 for DSSTox, with MCCs between 0.277 and 0.401, 0.228 and 0.495, and 0.289 and 0.383, respectively (Supplementary File, Table S35). The direction of the change depended on the balancing scheme: more aggressive balancing lowered the estimate for CLUE and COCONUT but raised it for DSSTox. A single corrected AUC per collection would therefore overstate the available precision, so the range is reported alongside the point estimate throughout. Finally, the molecules discarded by matching were compared with those retained to assess whether matching preferentially excluded target-relevant chemistry. Across the 14 substructure indicators with Benjamini–Hochberg correction, the retained external sets were enriched rather than depleted in hydroxamate (DSSTox 0.49% versus 0.09%, odds ratio 5.91, 95% CI 2.17–16.05, adjusted *p* = 0.0012) and in chelating motifs generally (DSSTox odds ratio 1.28, 1.11–1.47, adjusted *p* = 0.0027; COCONUT 1.18, 1.04–1.34, adjusted *p* = 0.0249), while thiourea prevalence did not differ in any collection (adjusted *p* = 0.91, 0.55, and 0.12). These results do not indicate preferential exclusion of the chemistry of interest by matching.

### 2.9. Multilevel Validation and the Limits of Factor Separation

Because source publication, laboratory, assay organism, assay protocol and chemotype vary together in this corpus, document-grouped cross-validation changes all of them at once. The extent of this coupling was quantified, and validation was repeated under every grouping that the data permit (Supplementary File, Table S36). Document accounts for 98.4% of the variation in assay organism, 81.8% of the variation in scaffold class and 85.9% of the variation in endpoint type. Only three of the 77 named documents report more than one organism; these contain 49 molecules and yield 111 within-document pairs that differ in organism, of which 22 are discordant on the potency label. Within-document comparisons are better supported for the other factors, with 55 documents, 913 molecules and 558 outcome-discordant pairs across scaffold classes and 10 documents, 217 molecules and 93 outcome-discordant pairs across endpoint types. Assay protocol cannot be examined at all: the assay-type field of these exports records the depositing resource rather than the experimental procedure, and every available protocol proxy is nested within the source document.

Publication-preserving validation gave similar document-grouped and leave-one-document-out estimates. For UI-Ref against CLUE, the AUC was 0.915 (95% CI 0.903–0.926) without grouping, 0.766 (0.747–0.785) with document grouping and 0.755 (0.735–0.775) under leave-one-document-out over 77 folds. The corresponding document-grouped and leave-one-document-out values were 0.841 and 0.836 for COCONUT, respectively, and 0.762 and 0.761 for DSSTox, respectively. Composite document–organism and document–organism–scaffold keys were audited but not interpreted as stricter validation: they split a source publication into multiple groups, placing the same document in training and test data. This affected 24 of 1218 positive test assignments (2.0%) for the document–organism key and 845 of 1218 (69.4%) for the document–organism–scaffold key; the previously computed AUCs for those keys are therefore withdrawn (Supplementary File, Table S36). For the potency task, the AUC fell from 0.866 (0.835–0.893) without grouping to 0.691 (0.646–0.735) with document grouping and 0.705 (0.660–0.749) under leave-one-document-out, whereas grouping by organism alone left performance at chance (0.532, 0.487–0.576; seven groups).

Mixed-effects models locate the variance explicitly. A linear mixed model of median pAct with a document-level random intercept, fitted on the 1082 documented molecules carrying a quantitative value over 76 documents, gave a document variance of 0.876 (SE 0.244) against a residual variance of 0.455, an intraclass correlation of 0.658. Adding descriptor fixed effects capturing 95% of the descriptor variance left this essentially unchanged (document variance 0.905, SE 0.257; ICC 0.669), so the document-level component of reported potency is not explained by the molecular descriptors. The organism component, by contrast, is not identified: crossed with document, it was estimated at 0.793 with a standard error of 1.192, and nested within document, it was estimated at 0.369 with a standard error of 0.333. A Bayesian binomial mixed model of the potency label gave the same picture, with a document random-intercept standard deviation of 3.11 (latent ICC 0.746) against an organism standard deviation of 0.235 crossed (ICC 0.004) and 0.175 nested (ICC 0.002). An organism-only model returned a variance of 2.712 (SE 1.732) that exceeds the marginal variance of the outcome and is driven by two single-publication levels, which illustrates a confounding rather than an organism effect.

In these data, the confounding is composite and cannot be decomposed into separate components. Source publication is identifiable and dominant, whereas assay organism, assay protocol, and chemotype are not separately identifiable. The title and conclusions therefore refer to provenance-associated confounding rather than to an isolated effect of provenance independent of chemistry. We do not claim to have separated publication identity from the laboratory practices, assay conditions, and chemical series that covary with it.

### 2.10. Hierarchical Re-Analysis of Motif–Potency Associations

**Figure 8:**
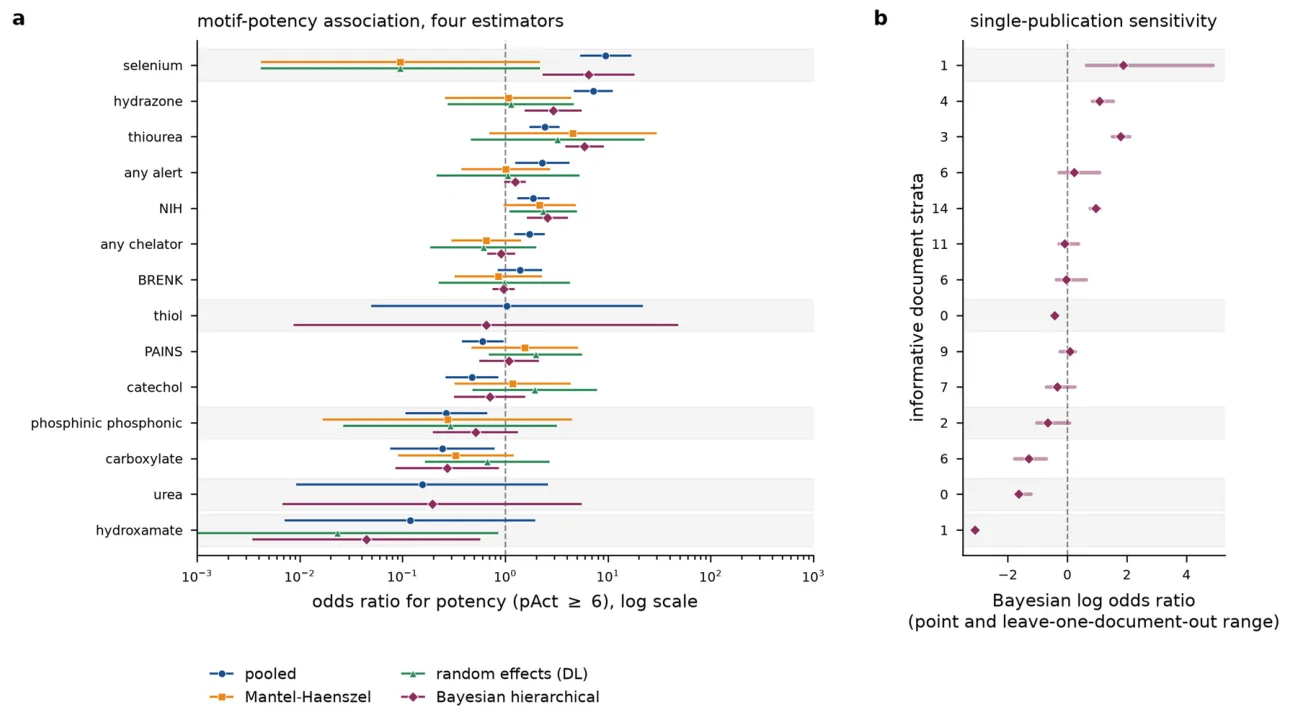
Motif–potency associations under four estimators, with single-publication sensitivity. (**a**) Odds ratio for potency (pAct >= 6) on a logarithmic scale for each of the 14 motif and structural-alert indicators, estimated by pooled logistic regression, Mantel–Haenszel stratification by source document, DerSimonian–Laird random-effects meta-analysis and Bayesian hierarchical logistic regression with a document random intercept. A fifth estimator, conditional logistic regression, is reported in Supplementary Table S37 but is not plotted. Bars denote 95% confidence or credible intervals; motifs with too few informative strata for a given estimator are omitted for that estimator. Rows shaded grey in both panels are the five motifs supported by fewer than three informative document strata (selenium 1, thiol 0, phosphinic/phosphonic 2, urea 0, hydroxamate 1), whose estimates are not identified by these data. (**b**) Leave-one-document-out range of the Bayesian log odds ratio for each motif, with the number of informative document strata on the vertical axis. Estimates whose range crosses zero are not identified by these data. There are n = 1110 molecules with a quantitative pAct, of which 178 are potent.

The stratified odds ratios of Section 2.6 rest on very few informative strata, and the Mantel–Haenszel estimator is unstable in that regime. The associations were therefore re-estimated with five estimators on the same data: pooled logistic regression, Mantel–Haenszel stratification, an exact within-document conditional estimator, DerSimonian–Laird random-effects meta-analysis over per-document log odds ratios, and Bayesian hierarchical logistic regression with a document-level random intercept. Leave-one-document-out refitting and Benjamini–Hochberg control of the false discovery rate across the 14 motifs were applied throughout (Figure 8; Supplementary File, Table S37). Only nine of the 14 motifs have at least three informative document strata; urea and thiol have none, whereas hydroxamate and selenium have one each.

The selenium results illustrate why a stratified point estimate is not interpretable with such sparse data. It occurs in 53 of the 1110 labeled molecules and has a single informative document stratum. Its pooled odds ratio is 9.51 (95% CI 5.34–16.94; Fisher *p* = 4.8 × 10^−14^, Benjamini–Hochberg-adjusted *p* = 3.3 × 10^−13^). The within-document estimates are 0.095 (0.004–2.175) by Mantel–Haenszel stratification and 0.114 (0.006–2.161) by the exact conditional method, both including 1. The random-effects estimate is 0.095 with *p* = 0.141 and an adjusted *p* of 0.563, whereas the Bayesian hierarchical estimate shrinks toward the pooled value and is positive (odds ratio 6.49, 95% credible interval 2.33–18.08). The Bayesian point estimate moves by 3.02 log odds units when a single publication is removed. These data do not identify the direction of the selenium–potency association. The apparent reversal between the pooled and the stratified estimate is a property of a single stratum, not a finding about selenium, and the same reasoning applies to any contrast drawn between selenium and hydrazone.

Across all motifs, the pooled and the clustering-aware analyses disagree systematically. Eleven of the 14 motifs survive false discovery rate control at 0.05 on the pooled Fisher tests, whereas none of the 12 estimable random-effects tests survives at either 0.05 or 0.10, the smallest adjusted *p*-value being 0.245. Between-document heterogeneity is substantial for the broadest indicators, with an I-squared of 45.6% for any chelating motif, 44.3% for any structural alert and 40.0% for Brenk alerts. Leave-one-document-out refitting reverses the sign of the hierarchical estimate for six of the 14 motifs, and a single publication is the most influential observation for three of them. A motif was treated as consistent only where its association held across several documents, organisms and scaffold classes with little between-document heterogeneity. One indicator meets that criterion: the NIH alert set appears in 14 informative strata across 31 documents, six organisms and 37 scaffold classes with an I-squared of 0% and a random-effects odds ratio of 2.34 (1.10–5.00, prediction interval 1.01–5.44); even this association does not survive multiplicity control (adjusted *p* = 0.245) and is therefore described as suggestive only. No motif–potency association in this corpus is established once source publication and multiple testing are both taken into account.

The Bayesian models were fitted using mean-field variational approximation rather than Markov chain Monte Carlo sampling. Variational credible intervals understate posterior variance, so the posterior probabilities of direction reported in Supplementary File, Table S37, are anti-conservative. Every conservative statement above rests instead on the random-effects prediction intervals and on the leave-one-document-out ranges, which do not depend on that approximation.

### 2.11. Sensitivity of the Applicability Domain, Conformal Coverage and Stage 1A

**Figure 9:**
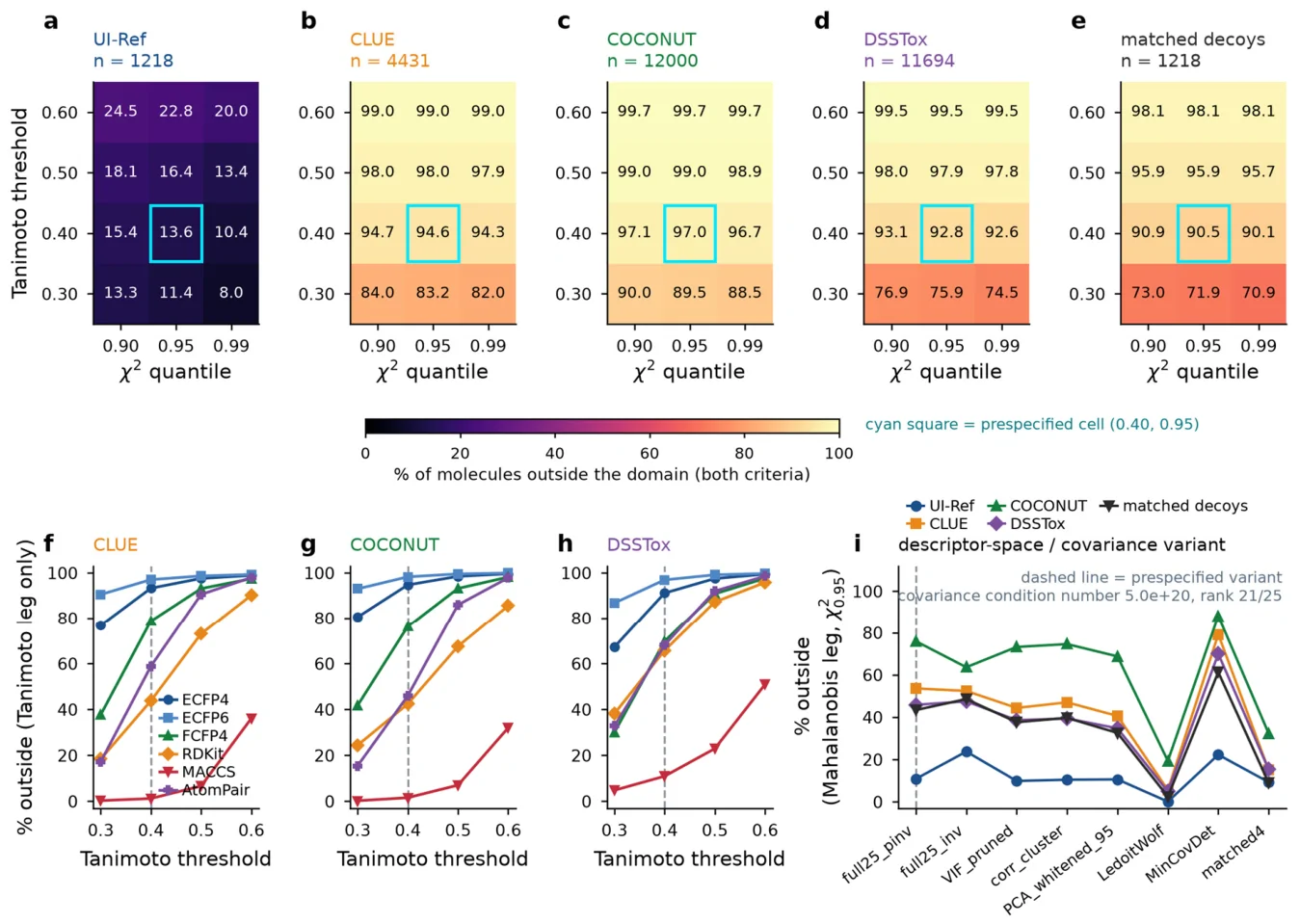
Sensitivity of the applicability domain to its thresholds and to the molecular representation. (**a**–**e**) Percentage of molecules outside the domain under both criteria, across the cross of ECFP4 Tanimoto threshold and Mahalanobis chi-square quantile, for UI-Ref (leave-one-out), CLUE, COCONUT, DSSTox and the property-matched decoys. The cyan square marks the prespecified cell (0.40, 0.95). (**f**–**h**) Percentage outside the Tanimoto criterion alone as a function of threshold, under six fingerprints, for the three external libraries; the dashed line marks the prespecified threshold. (**i**) Percentage outside the Mahalanobis criterion alone at the chi-square 0.95 quantile under eight covariance and descriptor-space variants; the dashed line marks the prespecified variant. The covariance matrix of the 25 descriptors has a condition number of 4.5 × 10^20^ and a numerical rank of 21 of 25, so the distance is not uniquely defined. Sample sizes are given in each panel.

The applicability domain was prespecified but not optimized, and its two criteria were therefore examined across thresholds and across molecular representations (Figure 9; Supplementary File, Table S39). At the prespecified cell, the proportions are: 94.58% of CLUE (95% Wilson interval 93.88–95.21, n = 4431), 96.99% of COCONUT (96.67–97.28, n = 12,000), 92.84% of DSSTox (92.36–93.30, n = 11,694), 90.48% of the matched decoys (88.70–92.00, n = 1218) and 13.63% of UI-Ref under leave-one-out evaluation. For UI-Ref, the appropriate interval is the document-clustered bootstrap interval of 8.97–19.24% (SD 2.69 percentage points), which is 2.7 times wider than the binomial interval, because molecules from the same publication are not independent.

The conclusion that external libraries lie overwhelmingly outside the domain is robust to the threshold. Across the 12 combinations of Tanimoto threshold (0.30 to 0.60) and chi-square quantile (0.90 to 0.99), the out-of-domain fraction spans 82.0–99.0% for CLUE, 88.5–99.7% for COCONUT, 74.5–99.5% for DSSTox and 70.9–98.1% for the matched decoys, so at least 70.9% of every external set lies outside the domain in every cell. It is not, however, robust to the molecular representation. At the prespecified threshold of 0.40, the six fingerprints examined give out-of-domain fractions from 1.20% with MACCS keys to 97.04% with ECFP6 for CLUE, a span of 95.9 percentage points, with comparable spans of 96.7, 86.0 and 94.3 percentage points for COCONUT, DSSTox and the decoys, respectively. The range attributable to fingerprint choice was therefore three to four times wider than that observed across the entire threshold grid, and the out-of-domain proportion is reported here as a property of the ECFP4-based domain rather than as a representation-independent property of the molecules.

The descriptor criterion is less numerically stable than its chi-square formulation implies. The covariance matrix of the 25 descriptors on UI-Ref has a condition number of 4.5e20 and a numerical rank of 21, with 13 descriptors showing a variance inflation factor above 10, 18 descriptor pairs correlated above 0.95 in absolute value, 17 of the 25 descriptors integer-valued, and univariate normality rejected for all 25. The Mahalanobis distance is therefore not uniquely defined: across eight covariance and descriptor-space variants, the out-of-domain fraction spans 73.6 percentage points for CLUE and 48.0 for UI-Ref itself, and replacing the pseudo-inverse with an ordinary inverse of the same singular matrix moves the UI-Ref fraction from 10.84% to 48.11%. The cutoff is also uncalibrated on its own reference set, excluding 12.73%, 10.84% and 7.31% of UI-Ref at the nominal 10%, 5% and 1% levels, respectively. In practice, this criterion contributes only 1.3 to 2.3 percentage points beyond the fingerprint criterion. It is accordingly treated as an uncalibrated conservative flag and not as a 95% probability statement, and Supplementary File, Table S39, reports a variance-inflation-pruned 16-descriptor subset as the numerically more stable alternative.

Within the prespecified applicability domain, cross-validated AUCs were higher. At the prespecified cell, averaged over five rotating document-grouped splits, in-domain retention was 40.9 +/− 13.6% for Stage 1A, 41.3 +/− 2.9% for Stage 1B, and 49.4 +/− 15.1% for Stage 2, while then AUC increased from 0.768 to 0.824, from 0.877 to 0.902, and from 0.658 to 0.780, respectively. Tightening the Tanimoto threshold to 0.60 raised the Stage 1B AUC to 0.948 +/− 0.042 while retaining 14.8% of molecules. The mean conformal prediction-set size decreased monotonically with the threshold, from 1.257 to 1.094 for Stage 1B and from 1.664 to 1.384 for Stage 2 at alpha = 0.10, with the singleton fraction rising from 74.3% to 90.6% and from 33.6% to 61.6%, respectively. The threshold therefore trades sample retention against discrimination and prediction-set decisiveness; because neither AUC nor MCC exhibited an interior optimum, no value was empirically optimal, and the prespecified threshold was retained (Supplementary File, Table S40; Figure S10).

The exchangeability assumption and empirical coverage were then evaluated rather than assumed. Nonconformity-score distributions differed between calibration and test document groups for all three model-based stages (two-sample Kolmogorov–Smirnov D = 0.166, 0.088, and 0.222; median *p* = 0.017, 0.038, and 7.9 × 10^−4^ for Stages 1A, 1B, and 2, respectively). Per-document coverage at alpha = 0.10 is six to eight times more dispersed than binomial sampling permits, with dispersion ratios of 8.32, 5.97 and 6.11 and homogeneity chi-square values of 441.0, 358.1 and 360.3 on 53, 60 and 59 degrees of freedom, respectively; per-document coverage ranges from 0.000 to 1.000 for Stage 1A and from 0.125 to 1.000 for Stage 2. For Stage 1A, the calibration split additionally contains only 11.8 minority-class molecules per fold against the 19 required for the label-conditional quantile at alpha = 0.05 to be attainable at all. These results provide evidence against exchangeability across source publications; accordingly, Table 3 classifies all three model-based stages as reporting empirical coverage without a guarantee. The caveat applies to every stage, not to Stage 1A alone. Coverage reported here is calibrated in aggregate over this corpus and should not be read as a guarantee for a molecule from a new publication. Stage 1A illustrates why: on a single document-grouped split, its coverage is 0.899 against a nominal 0.950, whereas over five rotating splits, it is 0.940 (0.922–0.954) at alpha = 0.05 and 0.895 (0.873–0.913) at alpha = 0.10. Its coverage is not stable across publications, and no single split estimates it reliably.

Finally, the dependence of Stage 1A on its label definition was quantified directly (Supplementary File, Table S38; Figure S9). Across the four negative-class definitions, the AUC ranges from 0.586 to 0.852 on descriptors and from 0.772 to 0.899 on fingerprints, and the MCC from 0.157 to 0.479 and from 0.271 to 0.425, on n = 895 to 934 molecules, respectively. The negative class is drawn from six to 11 source documents and 14 to 24 scaffold classes depending on the definition. Restricting the task to the six documents containing both classes—the only comparison without between-publication contrast—left 83 molecules and gave a descriptor model AUC of 0.393 (95% CI 0.270–0.520) and an MCC of −0.267. The AUC point estimate was below 0.5, but its confidence interval included 0.5; across all four label definitions, the within-document AUC ranged from 0.357 to 0.738, and the MCC interval included zero in three of eight settings. Varying the potency threshold from pAct >= 5 to pAct >= 7 changes the Stage 2 AUC from 0.644 to 0.698 on descriptors and from 0.690 to 0.661 on fingerprints, with a maximum at pAct >= 6.5; restricting the endpoint to IC^50^ alone gives 0.646 and 0.712, respectively, on 1003 molecules. A positive-unlabeled formulation of Stage 1A was also examined but was not considered valid for these data: whether a molecule carried a measured-active record was strongly associated with source publication, which contradicts the selected-completely-at-random assumption (labeled-positive fraction 0.043 to 1.000 across the 61 documents with at least five molecules; homogeneity chi-square 729.2 on 60 degrees of freedom, *p* = 1.1 × 10^−115^), and the resulting class prior is not identified. A Stage 1A model estimates the probability that a measured-active record exists for a molecule, not the probability that the molecule inhibits urease. Stage 1A is therefore reported as a narrow-scope diagnostic case study and is excluded from the general predictive cascade.

## 3. Discussion

Although urease provides the case study, the failure mode described here is not target-specific. It can arise whenever three conditions coincide: the positive set is assembled mainly from published congeneric series, the comparison collection is generated through a different selection or deposition process, and validation partitions individual molecules rather than their source publications. Under these conditions, closely related compounds from the same publication can occur in both the training and the test sets, while collection-specific structural patterns provide a more accessible classification signal than target activity. A cross-library model can therefore attain a high AUC by recognizing how and where molecules entered the dataset. Unequal collection sizes and dimensionality reduction can similarly create apparent chemical-space relationships that change when sampling is controlled. These results do not establish that target-related chemical differences are absent. What they establish is narrower: the conventional design cannot apportion the observed separation between target-related chemistry and provenance. That distinction, between a difference that cannot be attributed and a difference that is absent, is maintained throughout this study.

The necessary controls are practical and computationally inexpensive. First, provenance should be measured within the positive set by predicting depositing database and source publication, which provides an empirical reference scale for interpreting cross-library performance. Second, comparison compounds should be matched on the physicochemical properties that most readily distinguish collections, with SMDs reported before and after matching, and balance should be reported for every covariate the model can use rather than for the matching variables alone. Third, validation should be grouped by source publication as well as by scaffold; in UI-Ref, publication grouping was consistently the more stringent criterion, because individual publications generally represented narrower chemical series than broad scaffold classes. Fourth, each analysis should include a trivial baseline, a label-permutation null and a null model constructed within the same task so that the observed performance is compared against a quantity estimated on the same population. Finally, the applicability domain, its sensitivity to the thresholds chosen, and the fraction of the comparison collection lying outside it should all be reported. Together, these controls change the central question from whether two collections can be separated to whether the separation exceeds the provenance-associated signal and remains valid for the molecules being evaluated. The released repository implements all five steps.

These controls address a source of uncertainty distinct from model selection or scoring optimization. Our previous classification study showed that urease model performance was sensitive to the definition of bioactivity classes and that retaining an explicit indeterminate zone improved the treatment of compounds with intermediate IC_50_ values [40]. A subsequent benchmark of structure-based virtual screening showed that ligand rankings also depended on receptor structure, docking protocol, pose aggregation and data-fusion strategy [43]. The present results identify a further source of uncertainty upstream of both analyses: the organization of the underlying experimental record. Model optimization, careful label definition and provenance-aware validation are therefore complementary requirements rather than interchangeable ones.

Three findings are particularly relevant for urease research. First, the public record contains less independent biological evidence than its nominal size suggests. UI-Ref contains 1218 molecules, but only 178 reach pAct >= 6, and much of the dataset consists of a limited number of chemotypes contributed by a small number of laboratories. Only three publications tested compounds against more than one organism, which is why the contribution of assay organism cannot be separated from that of publication (Section 2.9). Dataset size alone therefore does not capture the chemical and biological coverage of the evidence. Second, simple drug-likeness summaries provide an incomplete description of this collection. Brenk alerts flag 84.8% of UI-Ref, and 57.3% of the molecules contain a nickel-chelating motif. Chelation may be mechanistically relevant to urease inhibition, but it can also affect selectivity, assay behavior and downstream development. Compliance with broad property rules [44,45] should therefore not be presented as evidence of overall drug-likeness when structural-alert burden and properties associated with compound attrition are omitted [46]. Third, no chemotype–potency association in this corpus survives adjustment for source publication together with control of the false discovery rate. The aggregated data cannot separate the effect of a motif from the effect of the laboratory, assay protocol or chemical series in which that motif was studied, and for most motifs, the available strata are too few for the direction of a stratified estimate to be interpreted at all.

These circumstances do not render computational screening uninformative. Previous urease studies have combined chemotype-focused machine learning, pharmacophore filtering, molecular docking, molecular dynamics and drug repurposing to prioritize experimentally tractable candidates [47,48]. The present analysis constrains the interpretation of those outputs. Chemical-space distance from known inhibitors, favorable docking scores and compliance with physicochemical filters are useful prioritization criteria, but none independently establishes urease inhibition, safety or clinical relevance. Establishing robust chemotype effects will require comparisons within well-characterized series or prospective measurements of structurally diverse compounds under a shared experimental protocol. Such data would also supply the measured-inactive examples and the cross-organism coverage required for more reliable predictive models.

### 3.1. Relation to Chemical Curation and to Established Validation Guidelines

Each resource used in this study is organized by chemical structure and applies substantial chemical curation. ChEMBL applies a published, open-source structure-curation pipeline comprising sanitization, functional-group normalization, charge neutralization, salt stripping and parent-structure generation and normalizes reported endpoints to a common combination of type, relation, value and unit, from which a potency value is derived. PubChem standardizes deposited substances into unique compound records and serves 49 distinct computed properties through its programmatic interface, spanning constitutional, physicochemical, stereochemical, three-dimensional-conformer and fingerprint descriptors. DSSTox enforces consistency between substance and structure identifiers, assigns quality-control levels, and distributes derived MS-Ready and QSAR-Ready structure forms alongside the registered structure. The Drug Repurposing Hub confirms the identity and purity of every physical sample by ultra-performance liquid chromatography–mass spectrometry before the compound enters the annotated library. COCONUT 2.0 re-curated its content and distributes precomputed molecular properties with each entry. These operations are chemical, and the descriptors analyzed here are themselves chemical quantities. Table 1 and Supplementary File, Table S41, provide resource-specific documentation.

The narrower claim is that, in a cross-collection comparison, collection membership is strongly associated with the curatorial and sampling processes that generated each resource, including its inclusion criteria, the publications from which records were abstracted, and the sampling behavior of the medicinal-chemistry literature. This association can be quantified: within UI-Ref, both depositing database and source publication were predictable from the 25 descriptors at the levels reported in Section 2.2, and a null model restricted to features describing a molecule’s relative position within its source library reached an AUC of 0.995 in the cross-collection task itself (Section 2.8). The title and conclusions therefore refer to provenance-associated confounding in cross-collection comparisons, not to the absence of chemistry from the databases.

The cascade can also be assessed against the OECD principles for the validation of quantitative structure–activity relationship models: a defined endpoint; an unambiguous algorithm; a defined domain of applicability; appropriate measures of goodness of fit, robustness and predictivity; and, where possible, a mechanistic interpretation [49]. Table 4 records how each principle is addressed and the limits of that assessment. The endpoints and label semantics are explicit (Table 2), the implementation and frozen configuration are reproducible, and the applicability domain and performance diagnostics are reported with sensitivity analyses. The fourth principle is only partly met for the intended non-regulatory ranking use because no publication-independent external test set exists, and all model-based conformal results are empirical rather than guaranteed.

A mechanistic interpretation is not available from these models. The cascade uses physicochemical descriptors and circular fingerprints and contains no representation of the dinuclear nickel active site, metal coordination or binding mode; descriptor attributions are model attributions, not mechanistic evidence. Because the OECD principle is qualified by ’where possible’, this absence does not by itself invalidate a reproducible statistical ranking tool used within its explicitly stated scope. It does preclude mechanistic or regulatory claims and prevents the cascade from being presented as a mechanistically interpretable regulatory (Q)SAR model.

### 3.2. Limitations

The numerical estimates reported here are specific to urease and to the database snapshots analyzed. Although the failure mode may apply to other target-focused collections, the provenance AUCs and out-of-domain fractions should not be transferred directly to other systems. The estimate of provenance-associated separability, moreover, captures only the factors that could be measured reliably, namely, depositing database and source publication. Additional sources of structure, including assay protocol, reporting period, laboratory-specific research interests and the synthetic accessibility of particular chemical series, may contribute further. Assay protocol in particular is not recorded independently of source document in these exports and therefore cannot be separated from it (Section 2.9). The measured reference scale should consequently be regarded as a lower bound on provenance-associated separability rather than as a complete account of the confounding structure. Because it is estimated on a different task from the cross-library classifiers, it is reported as a diagnostic reference rather than as a decision threshold.

The comparison collections and activity labels introduce further limitations. Samples of 12,000 COCONUT molecules and 11,694 molecules from a 100,000-substance DSSTox extract were analyzed, whereas CLUE was included in full. Absolute overlap estimates therefore depend on the sampled collections. This dependence was assessed through repeated size-controlled analyses and quantified with confidence intervals, but it was not eliminated. Negative evidence was also limited. Although 98 molecules had at least one measured-inactive record, only 59 met the stricter definition of an unambiguous inactive used in the measured-active-against-measured-inactive task, and these originated from six publications and 24 scaffold classes. The resulting class imbalance produced an MCC confidence interval that included zero and empirical conformal coverage below the nominal level, and that stage is therefore reported as a narrow-scope diagnostic. The available data support molecular ranking more strongly than classification at a fixed decision threshold.

Activity measurements were heterogeneous. IC_50_, K_i_, K_d_ and EC_50_ values were placed on a common logarithmic scale, but these endpoints are not experimentally equivalent and were obtained under different assay conditions. The source reports did not contain sufficient information to normalize measurements against a shared internal standard across laboratories. Consistent with this heterogeneity, 82 molecules differed by more than one logarithmic unit among their reported activity values. The applicability domain also depends on methodological choices. Its ECFP4 Tanimoto threshold of 0.40 and accompanying Mahalanobis-distance criterion were fixed before model fitting, which avoided tuning these thresholds to the evaluation data but did not establish their optimality. Section 2.11 quantifies how much the reported fractions depend on both choices. Finally, approximately two-thirds of UI-Ref consists of measurements against jack bean urease, a widely used experimental enzyme that does not represent the therapeutic targets of most anti-infective applications. 

This study is computational and does not provide prospective experimental validation of predicted activities, structural associations, or screening candidates. The corrected classifier should therefore not be regarded as a substitute for biochemical testing. Its contribution is methodological: it provides a framework for prioritizing molecules that explicitly accounts for publication provenance, label ambiguity, molecular standardization, calibration, and applicability-domain limitations.

## 4. Materials and Methods

### 4.1. Data Sources and Assembly

Urease-inhibitor records were compiled from ChEMBL [9], BindingDB [11] and PubChem BioAssay [12] exports downloaded on 9 August 2025. The scope, curation model and computed descriptors of each resource, together with the data version used, are summarized in Table 1. Pooling the primary exports yielded 3038 records. Assay-level metadata were retained for each record: source database, source identifier, assay identifier, assay organism, activity type, value, units, activity outcome and source document (digital object identifier, PubMed identifier or ChEMBL document identifier).

Ambiguous PubChem assay identifiers were resolved against the deposited assay descriptions through the PubChem Power User Gateway representational state transfer (PUG-REST) interface, which recovered the target protein accession, target name, source publication digital object identifier and journal for each. Assays were classified as urease-relevant when the target accession corresponded to a urease (P07374, Canavalia ensiformis; P69996 and Q0PXQ5, *H. pylori*), when the resolved organism was ureolytic, or when the assay name referred to urease; 61 of 159 resolved assays were not urease assays, and their records were excluded. This step removed 312 records and 93 molecules that would otherwise have entered the set only through cytotoxicity, antiviral or antioxidant counter-assays.

The comparison collections were the Drug Repurposing Hub accessed through CLUE [13] (4431 molecules after standardization, annotated as launched, in a clinical phase, or withdrawn), COCONUT [14,15] (a random sample of 12,000 from 641,651 natural products) and DSSTox accessed through the CompTox Chemicals Dashboard [15] (a random sample of 11,694 from a 100,000-substance extract). Sampling seeds were fixed at 42 and are recorded in the released configuration. The data version used for each resource is given in Table 1. 

### 4.2. Structure Standardization

All collections were processed through a single pipeline implemented with RDKit [50], comprising sanitization, largest-fragment selection, functional-group normalization, canonical tautomer selection and InChIKey generation, with the standardized SMILES re-parsed as a validity check [29]. Reionization was excluded after it was found to convert malformed depositor SMILES of phosphinic acids into an invalid pentavalent phosphorus form; nine such molecules were repaired to the neutral pentavalent acid ([P+](=O)O to [PH](=O)O) before standardization. Two mutually inconsistent standardization variants present in the earlier analysis, one applying uncharging without tautomer canonicalization and one applying metal disconnection with canonicalization, assign different identities to 27.5% of structures; a single frozen pipeline was therefore applied throughout. The released application evaluates both conventions for every query and withholds a prediction when their disagreement changes the applicability-domain assessment, because molecular identity and domain status are then convention-dependent. Molecules were deduplicated by InChIKey, with provenance aggregated rather than discarded.

Twenty-five physicochemical descriptors were computed with RDKit, with definitions and units given in Supplementary File, Table S30, together with Morgan/ECFP4 fingerprints at radius 2 and 2048 bits [30]. Bemis–Murcko scaffolds were assigned per molecule [33]. Descriptor completeness on the final set is 1.000. 

### 4.3. Activity Aggregation

Only IC_50_, K_i_, K_d_ and EC_50_ values reported in nanomolar units were converted to pAct = 9 - log10(value in nM). Records reporting minimum inhibitory concentration, IC_90_, GI_50_, LC_50_, percent inhibition or inconsistent units were retained with flags but excluded from potency modeling. For each molecule, the median, minimum, maximum and spread of pAct across records were recorded, together with the set of activity outcomes and whether any record was explicitly active or inactive.

### 4.4. Provenance Reference Scale and Pairwise Classifiers

The provenance reference scale was measured within UI-Ref only. Molecules exclusive to one depositing database were classified by source with a random forest (300 trees, class-balanced weights, seed 42) under 5-fold stratified cross-validation, on descriptors and on ECFP4; alternative source definitions (majority source, non-exclusive and record-level) are reported in Supplementary File, Table S5. Document membership was predicted one versus rest for each of the 12 largest source documents.

Pairwise UI-Ref-against-collection classifiers were evaluated under five designs: (a) the conventional stratified 70:30 split with random 5-fold cross-validation; (b) GroupKFold by source document; (c) GroupKFold by Bemis–Murcko scaffold; (d) property-matched negatives; and (e) matched negatives with document grouping. Matching was 1:1 greedy nearest-neighbor matching without replacement on standardized molecular weight, cLogP, TPSA and heavy-atom count, with balance reported as SMDs before and after matching (Supplementary File, Table S7). All point estimates were accompanied by percentile confidence intervals based on 2000 bootstrap samples; label-permutation analyses used 100 replicates per design, and dispersion was reported for every estimate in the main text and Supplementary Tables. Near-duplicate leakage across split boundaries was quantified at Tanimoto thresholds of 0.9, 0.7 and 0.4 (Supplementary File, Table S9). Model attributions were derived with TreeSHAP, summarized as the mean absolute SHAP value per descriptor, and compared across designs by Spearman rank correlation [35,36]. 

### 4.5. Chemical Space

Two-dimensional projections of ECFP4 fingerprints were computed using PCA, t-SNE (perplexity 30), and UMAP (15 neighbors, minimum distance 0.1). PCA was applied to the 2048-bit fingerprint matrix, whereas t-SNE and UMAP used Tanimoto distance. Analyses were performed under both the unbalanced sampling scheme of the conventional analysis and equal sample sizes of n = 1218 per collection over 20 independent draws [51]. Nearest-neighbor provenance was computed in the full fingerprint space at k = 1, 5 and 25 and, for comparison, in each two-dimensional embedding. Enrichment is defined as the observed neighbor fraction divided by the expectation from collection proportions, with bootstrap intervals. Clustering used k-means on the UMAP embedding across a range of k and Butina clustering at a Tanimoto threshold of 0.4 [37]. Cluster–label agreement is reported as AMI against collection, source document and assay organism, with permuted-label nulls.

### 4.6. Structural Alerts and Motif Analysis

PAINS, Brenk and NIH alerts were assigned with the RDKit filter catalogue [38,39,50]. Nickel-chelating and related motifs (hydroxamate, thiourea, urea, catechol, hydrazone, thiol, carboxylate, phosphinic/phosphonic and selenium) were assigned by SMARTS patterns validated against positive and negative controls comprising acetohydroxamic acid, thiourea, urea, ebselen, catechol, benzoic acid, cysteine, phenylhydrazone, phosphonoformic acid, aspirin and caffeine; all eleven controls behave as specified (Supplementary File, Table S17). The patterns are frozen in the analysis package and revalidated against the same controls at every run (Supplementary File, Table S33). Motif variance structure is reported as the between-document ICC. The association between motif and potency (pAct >= 6) is reported as pooled Fisher odds ratios and as Mantel–Haenszel estimates stratified by source document, with a homogeneity test and the count of informative strata; motifs with non-finite stratified estimates or fewer than two informative strata are reported but not counted as testable. Because the number of informative strata is small for most motifs, the same associations were additionally estimated by random-effects meta-analysis and by Bayesian hierarchical logistic regression with a document-level random intercept, with leave-one-document-out sensitivity analysis and Benjamini–Hochberg control of the false discovery rate across motifs (Section 4.9).

### 4.7. Cascade Construction, Calibration, Applicability Domain and Conformal Prediction

Each cascade stage is specified with its positive class, its negative class and the experimental meaning of that negative class (Table 2). Stage 1A compares measured-active against measured-inactive molecules and is reported as a narrow-scope diagnostic rather than as a general predictive stage, for the reasons given in Section 2.7 and Section 2.11. Stage 1B compares UI-Ref membership against property-matched presumed-negative decoys drawn from COCONUT and DSSTox and is reported as a ranking tool only. Stage 2 classifies potent (pAct >= 6) against weak among molecules with quantitative values. Stages 3 and 4 are identified as circular and as library-membership tasks, respectively, and are excluded from predictive claims.

The models compared at each stage were a majority-class baseline, logistic regression, a random forest, XGBoost, LightGBM and a k-nearest-neighbor Tanimoto baseline, evaluated on ECFP4; on descriptors and on their union; and under random, scaffold-grouped and document-grouped 5-fold cross-validation, and scored by AUC, MCC, balanced accuracy and Brier score with bootstrap intervals [31,38]. Probability calibration used Platt scaling and isotonic regression fitted on a held-out calibration split, assessed by Brier score, reliability curves and ECE [41]. The applicability domain combines a prespecified ECFP4 Tanimoto threshold of 0.40 to the nearest training molecule with a Mahalanobis-distance criterion in descriptor space at the 0.95 chi-square quantile; performance is reported inside and outside the domain, and the fraction of each comparison collection lying outside it is tabulated (Supplementary File, Table S26). Inductive conformal prediction was applied at each stage with alpha = 0.05, 0.10, and 0.20; empirical coverage was compared with nominal coverage, and the fractions of singleton, both-class, and empty prediction sets, together with singleton accuracy, were reported [25,26]. Cascade propagation reports per stage, the number of molecules entering, the number inside the domain, the number with an informative conformal set and the number surviving to the next stage.

### 4.8. Task-Matched Null Models and Balancing Sensitivity

Four null models were constructed inside the corrected UI-Ref-against-collection design, each using the same random forest, the same document-grouped folds and the same seed as the observed model. The first replaces each molecule’s descriptor vector with the mean descriptor vector of its provenance block, computed with random external blocks and, separately, with chemically coherent external clusters. The second uses provenance metadata only and is reported as degenerate, because the external libraries carry none of the record, assay, document, organism or endpoint fields that UI-Ref carries, so metadata availability separates the classes perfectly. The third uses five library-assembly features that describe only a molecule’s position within its own library, namely, the frequency of its Bemis–Murcko scaffold in that library and the density of its ECFP4 neighborhood there. The fourth permutes the collection labels within matched pairs over 200 replicates. Differences in AUC were estimated by paired bootstrap over 2000 replicates, with the same test molecules resampled for both models in each replicate, and by the DeLong test for correlated receiver operating characteristic curves where both models score the same molecules; the DeLong implementation was checked against a structural-component computation of the same covariance and against a bootstrap standard error. Provenance labels within the external libraries were the CLUE development status and the numeric width of the DSSTox substance identifier; COCONUT releases no provenance field. Provenance predictability was additionally treated as a covariate by computing the AUC conditional on decile of the library-assembly score.

SMDs were computed before and after matching for all 25 descriptors and for the 14 substructure indicators, with covariates exceeding an absolute value of 0.1 flagged. Four alternative balancing schemes were applied to the full descriptor block: entropy balancing by dual Newton solution of the Hainmueller moment conditions, with the achieved moment gaps and effective sample sizes reported; coarsened exact matching, which, on all 25 coarsened descriptors, leaves too few common strata to support the comparison and was therefore applied to the leading principal components; propensity-score weighting for the average treatment effect on the treated, with common-support and weight diagnostics; and 1:1 matching on the full 25-descriptor Mahalanobis distance. The external molecules retained by matching were compared with those discarded across the 14 substructure indicators by proportion tests with Benjamini–Hochberg correction.

### 4.9. Multilevel Validation and Hierarchical Motif Models

Normalized conditional entropies H(Y|document)/H(Y) were computed for assay organism, scaffold class and endpoint type. Cross-validation was repeated with folds grouped separately by document, organism, scaffold and endpoint, and under leave-one-document-out, with the number of effective groups reported for each. Proposed composite document–organism and document–organism–scaffold keys were subjected to an explicit fold-integrity audit. Because these keys split publications across folds, their AUCs were withdrawn rather than interpreted as publication-level generalization. The number of within-document comparisons available for each factor was counted directly as the number of documents containing more than one level, the number of molecules they contain, the number of within-document pairs differing in that factor and the subset of those pairs discordant on the outcome. Variance components were estimated with a linear mixed model for median pAct and a Bayesian binomial mixed model for the potency label, with document and organism random intercepts fitted separately, crossed and nested; descriptor fixed effects were entered as the leading principal components retaining 95% of descriptor variance. Standard errors are reported for every variance component so that identified and unidentified components can be distinguished.

Motif–potency associations were estimated by pooled logistic regression; Mantel–Haenszel stratification by source document; an exact within-document conditional estimator; DerSimonian–Laird random-effects meta-analysis with tau-squared, Cochran’s Q, I-squared and a prediction interval; and Bayesian hierarchical logistic regression with a document-level random intercept and a weakly informative prior on the motif coefficient. The Bayesian models were fitted using mean-field variational approximation, so their credible intervals are anti-conservative; conservative statements therefore rest on the random-effects prediction intervals and the leave-one-document-out ranges. Every motif was refitted under leave-one-document-out to identify associations driven by a single publication, and Benjamini–Hochberg correction was applied across the 14 motifs. Motifs with fewer than three informative document strata are reported but not interpreted directionally. The number of distinct documents, organisms and scaffold classes in which each motif occurs is reported so that consistency across those factors can be assessed.

Applicability-domain sensitivity was evaluated over the full cross of ECFP4 Tanimoto thresholds of 0.30, 0.40, 0.50 and 0.60 with Mahalanobis chi-square quantiles of 0.90, 0.95 and 0.99 and with each criterion applied alone. The fingerprint leg was repeated under ECFP4, ECFP6, FCFP4, RDKit topological, MACCS and atom-pair fingerprints, and the descriptor leg under the full 25 descriptors with pseudo-inverse and ordinary inverse, a variance-inflation-pruned subset, a correlation-clustered subset, PCA-whitened descriptors retaining 95% of variance, Ledoit–Wolf shrinkage and minimum covariance determinant estimation. Covariance conditioning, variance inflation factors, univariate normality and multivariate normality were quantified to establish the stability of the criterion. Stage performance; conformal empirical coverage at alphas of 0.05, 0.10 and 0.20; mean prediction-set size; and the singleton, two-class and empty-set fractions were recorded for every domain definition over five rotating document-grouped splits, which supplies fold-to-fold dispersion. Evidence against exchangeability between calibration and test document groups was assessed using two-sample Kolmogorov–Smirnov and Mann–Whitney tests on the nonconformity scores, together with a per-document coverage analysis, a homogeneity chi-square test, a dispersion ratio relative to binomial sampling, and an assessment of whether the label-conditional quantile was attainable given the calibration sample size. Each stage was then classified as reporting empirical coverage only or as meeting the conditions required for a coverage guarantee. Stage 1A was additionally re-evaluated under four negative-class definitions, five potency thresholds, three endpoint-merging strategies, two molecular representations, a within-document restriction and a positive-unlabeled framing whose selected-completely-at-random assumption was tested for homogeneity across documents.

### 4.10. Software and Availability

Analyses used Python 3.12 with RDKit (>=2023.9), scikit-learn (>=1.3), XGBoost (>=2.0), LightGBM (>=4.0), SHAP (>=0.44), UMAP-learn (>=0.5) and statsmodels (>=0.14) [32,50,51]. The random seed was 42 throughout, and all thresholds were fixed in a configuration module. All analyses can be reproduced under the specified package versions using the numbered scripts in the same repository. The curated dataset, numbered reproduction scripts, frozen model bundle, local application, and data dictionary are available at: https://github.com/DanielBustosG/Urease-provenance-audit (accessed on 6 October 2026).

## 5. Conclusions

Cross-collection comparisons can reflect library provenance more strongly than target-related chemistry. Within UI-Ref, molecular descriptors predicted the depositing database with an AUC of 0.954 and the source publication with a median AUC of 0.997, although every molecule belonged to the same reference set. After matching the external collections on physicochemical properties and grouping validation by source publication, the cross-library AUCs decreased to 0.762–0.841. The residual separation exceeded the within-pair label-permutation null, which indicates a detectable difference between UI-Ref and the external collections; however, it remained below the library-assembly nulls. The design therefore cannot distinguish target-related chemistry from residual provenance structure, and the observed separation is substantially smaller than conventional analyses suggest. These results do not support strong claims that reported urease inhibitors constitute a broadly distinct chemical space, including the proposed separation from toxicologically flagged compounds. They also do not establish that no such distinction exists: the analysis identifies a difference that cannot be attributed, not the absence of a difference.

The resulting prediction framework therefore defines its scope and labels explicitly. The curated dataset distinguishes reported activity from potency, the potency model is evaluated under publication-grouped validation, probabilities are calibrated, conformal prediction identifies uninformative cases, and the applicability domain excludes approximately 93–97% of the external-library molecules examined here. The accompanying application implements these rules by withholding predictions when a molecule lies outside the relevant domain or when its identity depends on the standardization convention. When exchangeability across source publications is not supported, the application reports empirical coverage as a diagnostic and does not claim a conformal coverage guarantee.

The worked compound illustrates why these checks are necessary. Under the standardization pipeline used to construct UI-Ref, the molecule corresponds to UIREF-1160, a measured urease inhibitor already present in the reference set. Under the alternative convention used by the earlier interface, it receives a different tautomeric identity, which changes its nearest neighbors and its applicability-domain assessment. The earlier interface nevertheless labeled it a non-inhibitor with a probability of 0.538. The corrected application does not convert this unstable result into a categorical prediction: it reports the identity conflict, withholds the affected stage-level verdicts, and returns the structural-alert result separately because that stage is rule-based rather than predictive. The complete application output, including both standardized identities, stage-level diagnostics, reasons for abstention, nearest neighbors, validation summaries and limitations, is provided in the Supplementary Application Report.

Model performance alone is therefore insufficient to establish the reliability of a target-focused chemical-space analysis. Provenance-aware validation, experimentally grounded negative labels, consistent molecular standardization, and explicit abstention rules each define the scope of the reported performance. In urease-inhibitor screening, abstention prevents the assignment of probabilities to molecules for which the available data do not support a prediction.

## Figures and Tables

**Table 1:** Scope, curation model and computed-descriptor provision of the six resources used in this study. Full details, including the complete enumeration of the PubChem computed-property set, the identifiers, and the source of every entry with its retrieval date, are given in Supplementary File, Table S41.

Resource	Scope and How Compounds Enter	Chemical Curation Performed by the Resource	Computed Descriptors Provided by the Resource	Contribution to This Study
ChEMBL	Manually abstracted bioactivity data from the medicinal-chemistry literature; 2,496,335 compounds, 21,123,501 activities, 1,740,546 assays, of which 1,673,227 are attributed to literature abstraction	Published open-source structure-curation pipeline: sanitization, functional-group normalization, charge neutralization, salt stripping and parent-structure generation	Standardized parent structures; activity type, relation, value and unit normalized to a common quadruple from which pChEMBL is derived	Source of urease assay records with document identifiers
BindingDB	Measured binding affinities between proteins and drug-like molecules, curated from the literature and imported from other resources; 3,241,782 binding data for 11,509 proteins and 1,440,011 molecules, of which approximately 1.6 million data are curated in-house	In-house curation of affinity values with target and ligand identity resolution	Affinity values (IC_50_, K_i_, K_d_, EC_50_) with units; structures as SMILES and InChI	Source of urease affinity records with document identifiers
PubChem BioAssay	Depositor-submitted bioassay results and substances, aggregated into compound records	Substance-to-compound standardization producing unique compound records	49 distinct computed properties are served by the programmatic interface, spanning structural identifiers, constitutional and physicochemical descriptors, six stereochemistry counts, a thirteen-term three-dimensional conformer block, the PubChem 2D substructure fingerprint, and bibliometric and annotation counts	Source of urease assay records; assay descriptions used to resolve 159 ambiguous assay identifiers
CLUE Drug Repurposing Hub	Curated collection of compounds with a clinical history, annotated by development phase; 4431 molecules after standardization in this study	Identity and purity of each physical sample confirmed by ultra-performance liquid chromatography–mass spectrometry before entry into the annotated library	Clinical-phase annotation, mechanism of action and target annotation	Comparison collection; source of the launched against non-launched diagnostic task
COCONUT	Aggregated open natural-product structures collected from open sources; a random sample of 12,000 from the 641,651 entries of the release used	Wholesale reassembly and re-curation of content in version 2.0	Precomputed molecular properties distributed with every entry	Natural-product comparison collection
DSSTox (CompTox Chemicals Dashboard)	Regulatory and environmental substance inventory; a random sample of 11,694 from a 100,000-substance extract	Quality control enforcing consistency between substance and structure identifiers, with assigned quality-control levels	Derived MS-Ready and QSAR-Ready structure forms distributed alongside the registered structure	Toxicological comparison collection

**Table 2:** Cascade stages and their label semantics.

Stage	Task	Positive Class	Negative Class
Stage 1A	Reported active versus measured inactive	At least one measured-active record and no measured-inactive record (836)	At least one measured-inactive record and no measured-active record (59)
Stage 1B	UI-Ref membership versus property-matched untested compound	UI-Ref member (1218; reported in a urease assay)	Property-matched COCONUT or DSSTox molecule (1218; urease activity unmeasured, not assumed inactive)
Stage 2	Potency: potent vs. weak	pAct >= 6 (178)	pAct < 6 (932)
Stage 3	Toxicophore-flagged vs. not	>=1 structural alert (3931)	No alert (2069)
Stage 4	Clinical status: launched vs. not	CLUE launched (2387)	CLUE non-launched (2044)

**Table 3:** Classification of each cascade stage as supporting a conformal coverage guarantee or as reporting empirical coverage only, against four explicit conditions.

Stage	Exchangeability Across Documents	Per-Document Coverage Dispersion	Calibration Size Sufficient	Classification
1A: Measured active vs. measured inactive	Rejected (KS D = 0.166, median *p* = 0.017)	8.32× binomial; coverage range 0.000–1.000	No: 11.8 minority molecules per fold against 19 required at alpha = 0.05	Empirical coverage only, no guarantee
1B: UI-Ref membership vs. matched decoy	Rejected (KS D = 0.088, median *p* = 0.038)	5.97× binomial; coverage range 0.300–1.000	Yes: 243.6 minority molecules per fold	Empirical coverage only, no guarantee
2: Potent vs. weak (pAct >= 6)	Rejected (KS D = 0.222, median *p* = 7.9 × 10^−4^)	6.11× binomial; coverage range 0.125–1.000	Yes: 35.6 minority molecules per fold	Empirical coverage only, no guarantee
3: Structural-alert status	Not applicable	Not applicable	Not applicable	Deterministic SMARTS rule; conformal validity not a meaningful question
4: CLUE launched vs. non-launched	Not evaluated	Not evaluated	Not evaluated	Not evaluated; excluded from predictive claims

**Table 4:** Assessment of the prediction cascade reported here against the five OECD principles for the validation of quantitative structure–activity relationship models [49].

OECD Principle	Requirement	Status in the Present Study	Evidence
1. A defined endpoint	The modeled effect must be unambiguously specified	Met	Each cascade stage has an explicitly defined endpoint and stated label semantics (Table 2). Stage 1A is measured active against measured inactive and is reported as a narrow-scope diagnostic; Stage 1B is UI-Ref membership against property-matched presumed negatives and is declared a ranking tool only; Stage 2 is potent (pAct >= 6) against weak among molecules with quantitative values.
2. An unambiguous algorithm	The model must be fully and reproducibly specified	Met	All models, hyperparameters, representations, splits and thresholds are fixed in a single prespecified configuration block; the fitted bundle is distributed with a SHA-256 checksum and verified on every run; numbered scripts regenerate every figure and table.
3. A defined domain of applicability	The chemical space in which predictions are valid must be defined	Met, prespecified, and now reported with its sensitivity	Nearest-neighbor ECFP4 Tanimoto >= 0.40 combined with a Mahalanobis-distance criterion at the chi-square 0.95 cutoff, both fixed before fitting. Out-of-domain fractions: CLUE 94.6%, COCONUT 97.0%, DSSTox 92.8%, matched decoys 90.4%, UI-Ref 13.6% under leave-one-out evaluation. Threshold and representation sensitivity are reported in Section 2.11 and Supplementary File, Table S39; the descriptor criterion is described there as an uncalibrated conservative flag rather than as a probability statement.
4. Appropriate measures of goodness of fit, robustness and predictivity	Internal and external performance must be quantified	Partly met for the stated non-regulatory ranking use	Performance is reported under document-grouped cross-validation with bootstrap confidence intervals and under leave-one-document-out evaluation (Supplementary File, Tables S24 and S36). Composite keys that split publications across folds were detected by an integrity audit, and their AUCs were withdrawn. Label permutation returns to chance. Every model-based conformal stage reports empirical coverage without a guarantee because exchangeability across source publications is rejected (Table 3). No held-out external test set independent of the source documents exists.
5. A mechanistic interpretation, where possible	Where feasible, the model should admit a mechanistic reading	Not available; limits mechanistic and regulatory interpretation	The cascade contains no representation of the dinuclear nickel active site, metal coordination or binding mode. Descriptor attributions are not mechanistic evidence. This limitation does not invalidate the explicitly bounded statistical ranking use, but it precludes mechanistic and regulatory claims.

## Data Availability

The derived data generated in this study, all analysis code, the frozen model bundle and the domain-aware application are openly available in the release repository at https://github.com/DanielBustosG/Urease-provenance-audit (accessed on 6 October 2026), together with numbered scripts that regenerate every figure and table in this manuscript from those inputs. These comprise the curated reference set UI-Ref (1218 molecules), the curated assay records (2879 records), the three standardized comparison collections (CLUE, n = 4431; COCONUT, n = 12,000; DSSTox, n = 11,694), the fitted cascade bundle with its SHA-256 checksum, and Supplementary Tables S1–S41; an archival digital object identifier will be minted on acceptance. Restrictions apply to the primary database exports from which the curated data were derived. These are third-party data obtained from ChEMBL 35, BindingDB (monthly release 2025-08), PubChem BioAssay (accessed on 9 August 2025), the CLUE Drug Repurposing Hub (annotation export, 9 August 2025), COCONUT 2.0 (dated release, 9 August 2025) and DSSTox via the CompTox Chemicals Dashboard version 2.5.3, and they are subject to the licenses of those resources, namely, CC BY-SA 3.0 for ChEMBL and for ChEMBL-derived BindingDB records, CC BY 3.0 for BindingDB-curated records, public domain for PubChem and DSSTox, CC0 for COCONUT, and non-commercial research use only for the CLUE Drug Repurposing Hub. They are therefore not redistributed here.

## Supplementary Materials

The following supporting information can be downloaded at: https://www.mdpi.com/article/10.3390/ijms27209159/s1.
