# Supplementary material for: Provenance-Associated Confounding in Cross-Collection Comparisons of Urease Inhibitors: A Quantified Reference Scale and a Domain-Aware Alternative
Source: Int J Mol Sci. 2026 Oct 15;27(20):9159. doi: 10.3390/ijms27209159 (PMC13705496; doi:10.3390/ijms27209159)
Supplement: Supplementary file 1 [file ijms-27-09159-s001.zip › Supplementary_File_Application_Report.pdf]

Supplementary Application Report

Complete worked output from the applicability-domain-aware urease prediction framework

Purpose of this file
This report preserves the complete output of the released application for the worked compound discussed in the manuscript. It records molecular identity, standardization, exact structural alerts, stage-level refusals, applicability-domain checks, conformal sets, nearest training molecules, validation summaries, and limitations. It is generated from the same prediction function used by the interactive application.

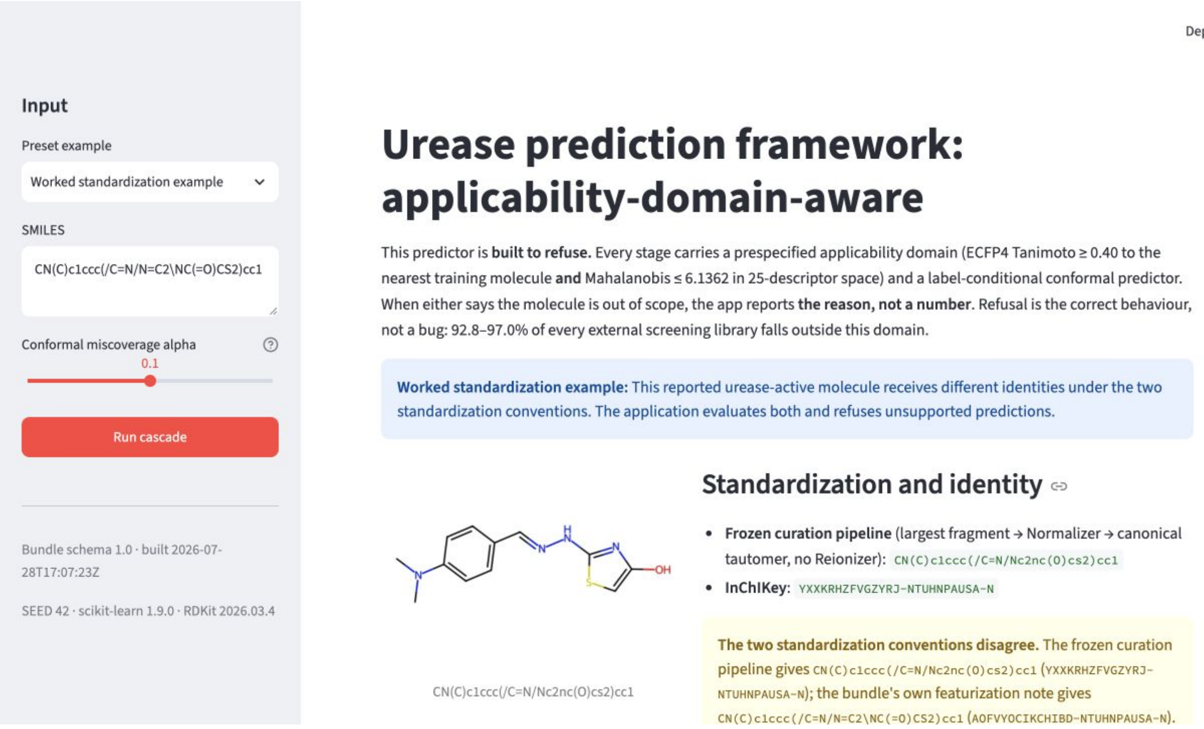

Application interface at the start of the worked example. Complete results follow on subsequent pages.

Worked example and molecular identity

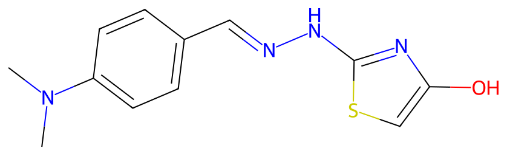

**Input SMILES**	`CN(C)c1ccc(/C=N/N=C2\NC(=O)CS2)cc1`
**Conformal alpha**	0.10
**Bundle schema**	1.0
**Bundle creation date**	2026-07-28T17:07:23.321816+00:00
**Random seed**	42

Standardization conventions

**Frozen curation SMILES**	`CN(C)c1ccc(/C=N/Nc2nc(O)cs2)cc1`
**Frozen curation InChIKey**	`YXXKRHZFVGZYRJ-NTUHNPAUSA-N`
**Bundle-note SMILES**	`CN(C)c1ccc(/C=N/N=C2\NC(=O)CS2)cc1`
**Bundle-note InChIKey**	`AOFVYOCIKCHIBD-NTUHNPAUSA-N`
**Conventions agree**	No
**Phosphinic repair applied**	No

Identity warning

The two conventions assign different tautomers and different InChIKeys to the same drawn compound. Because this changes the applicability-domain and conformal outcomes in several stages, the application does not silently select the more favorable convention.

UI-Ref record recovered under the frozen convention

**UI-Ref identifier**	UIREF-1160
**Measured activity**	pAct median 5.1367; IC50; 1 direct measurement
**Activity status**	Active: Yes; inactive: No
**Organism**	Sporosarcina pasteurii
**Source database**	BindingDB
**Source document**	10.1016/j.bioorg.2015.10.005

Exact structural-alert result

**Alert count**	3
**Matched alerts**	hzone_anil_di_alk(35), imine_1, Oxygen-nitrogen_single_bond
**Rule**	PAINS + BRENK + NIH SMARTS via rdkit.Chem.FilterCatalog
**Interpretive limit**	Zero alerts is NOT 'non-toxic'. No toxicity assay of any kind enters this label.

Overall application result

Five stages evaluated; five stages declined

Answered stages: 0. Refused stages: 5. At least one stage was outside its applicability domain: Yes. The reasons differ by stage and are documented in full below.

Stage 1B: resemblance to a previously assayed molecule

Application verdict: REFUSE

No class verdict is reported. Numeric outputs remain available below only as suppressed diagnostics, together with the reasons for refusal.

Label meaning

**Positive class**	UI-Ref member (reported urease activity of any magnitude)
**Negative class**	PRESUMED negative: property-matched COCONUT/DSSTox molecule, never assayed
**What the negative means**	NEVER ASSAYED AGAINST UREASE. Absence of evidence, not evidence of absence. Some are certainly actives.
**Honest interpretation**	USABLE ONLY AS A RANKING/ENRICHMENT TOOL. Its probability is P(looks like a molecule someone published a urease assay for), NOT P(inhibits urease). Must never be reported as 1A.
**Design**	1:1 greedy nearest-neighbour matching without replacement in z-scored MolWt/LogP/TPSA/NumHeavyAtoms space; median match distance 0.121 z-units; all four KS tests non-significant (D<=0.031, p>=0.59). UI-Ref inchikeys removed from the pool.
**Grouping**	primary_document of the positive; each decoy inherits its matched positive's document so the pair never splits across folds
**Class counts**	1218 positive; 1218 negative

Why the application declined to answer

1. OUTSIDE the applicability domain under the bundle_featurization_note convention: nearest training molecule is only Tanimoto 0.3400, below the preregistered 0.40 floor.

2. The two standardization conventions disagree about this molecule (frozen_curation -> CN(C)c1ccc(/C=N/Nc2nc(O)cs2)cc1; bundle_featurization_note -> CN(C)c1ccc(/C=N/N=C2\NC(=O)CS2)cc1), and the disagreement changes in_applicability_domain, conformal_set_size. A verdict that flips with tautomer convention is not a verdict.

Diagnostics under both standardization conventions

Convention	P(positive)	Nearest-neighbour Tanimoto	Mahalanobis	In domain	Conformal set	Training hit	Usable
frozen_curation	0.9376	0.4565 (floor 0.40)	3.6578 (cutoff 6.1362)	Yes	{positive}	No	Yes
bundle_featurization_note	0.6253	0.3400 (floor 0.40)	3.7525 (cutoff 6.1362)	No	{both classes} - UNINFORMATIVE	No	No

The reported probability is not the verdict when a stage is refused. It is shown only to document the complete application output.

Nearest training molecules under the frozen convention

Rank	Tanimoto	Training label	Training record	InChIKey and SMILES
1	0.4565	1	UIREF-0622; pAct median 6.8962; IC50|Ki; Canavalia ensiformis; CHEMBL1909545	MUDHKEQEBJQRRG-KPKJPENVSA-N CN(C)c1ccc(/C=N/NC(N)=S)cc1
2	0.4565	1	UIREF-0623; pAct median 6.9049; IC50|Ki; Canavalia ensiformis; 10.1016/j.ejmech.2011.09.009	MUDHKEQEBJQRRG-UHFFFAOYSA-N CN(C)c1ccc(C=NNC(N)=S)cc1
3	0.4151	1	UIREF-0861; pAct median 4.6912; IC50; Canavalia ensiformis; nan	SHNJCBATXVDZCQ-XDHOZWIPSA-N CN(C)c1ccc(/C=N/c2nc(-c3ccccc3)cs2)cc1

Validated performance stored in the released bundle

Cross-validation

Validation split	AUC (95% CI)	MCC (95% CI)	Balanced accuracy
document	0.904 (0.882-0.928)	0.586 (0.552-0.632)	0.768
random	0.980 (0.978-0.982)	0.868 (0.847-0.890)	0.934
scaffold	0.934 (0.910-0.959)	0.624 (0.424-0.759)	0.792

Held-out test and calibration

n	AUC	Brier raw	Brier Platt	Brier isotonic	ECE raw	ECE Platt
488	0.875	0.177	0.140	0.141	0.156	0.051

Conformal validation

alpha	Empirical coverage	Both classes	Empty set	Singleton accuracy
0.05	0.932	0.328	0.000	0.899
0.10	0.891	0.197	0.000	0.865
0.20	0.807	0.039	0.000	0.800

Stage 1A: measured active versus measured inactive

Application verdict: REFUSE

No class verdict is reported. Numeric outputs remain available below only as suppressed diagnostics, together with the reasons for refusal.

Label meaning

**Positive class**	measured Active (>=1 Active record, no Inactive record)
**Negative class**	measured INACTIVE (>=1 Inactive record, no Active record)
**What the negative means**	GENUINELY TESTED AND FOUND INACTIVE. The only real negatives that exist for this target.
**Honest interpretation**	USABLE BUT NARROW. Report with document-grouped CV and wide CIs. Cannot generalise beyond the 6 source papers.
**Design**	Severely imbalanced (93.4% positive) and structurally confined: all 59 negatives come from 6 publications + 14 document-less records, spanning 24 Murcko scaffolds. Document-grouped CV leaves as few as 0-20 negatives per fold.
**Grouping**	primary_document (StratifiedGroupKFold); document-less molecules pooled into one NODOC group
**Class counts**	836 positive; 59 negative

Why the application declined to answer

1. OUTSIDE the applicability domain under the bundle_featurization_note convention: nearest training molecule is only Tanimoto 0.3400, below the preregistered 0.40 floor.

2. The two standardization conventions disagree about this molecule (frozen_curation -> CN(C)c1ccc(/C=N/Nc2nc(O)cs2)cc1; bundle_featurization_note -> CN(C)c1ccc(/C=N/N=C2\NC(=O)CS2)cc1), and the disagreement changes in_applicability_domain, conformal_set_size. A verdict that flips with tautomer convention is not a verdict.

Diagnostics under both standardization conventions

Convention	P(positive)	Nearest-neighbour Tanimoto	Mahalanobis	In domain	Conformal set	Training hit	Usable
frozen_curation	0.9900	1.0000 (floor 0.40)	3.4274 (cutoff 6.1362)	Yes	{positive}	Yes	Yes
bundle_featurization_note	0.9300	0.3400 (floor 0.40)	3.3773 (cutoff 6.1362)	No	{both classes} - UNINFORMATIVE	No	No

The reported probability is not the verdict when a stage is refused. It is shown only to document the complete application output.

Nearest training molecules under the frozen convention

Rank	Tanimoto	Training label	Training record	InChIKey and SMILES
1	1.0000	1	UIREF-1160; pAct median 5.1367; IC50; Sporosarcina pasteurii; 10.1016/j.bioorg.2015.10.005	YXXKRHZFVGZYRJ-NTUHNPAUSA-N CN(C)c1ccc(/C=N/Nc2nc(O)cs2)cc1
2	0.6170	1	UIREF-1186; pAct median 4.5701; IC50; Sporosarcina pasteurii; 10.1016/j.bioorg.2015.10.005	ZMEAZVJWHIWUFB-VZUCSPMQSA-N O=[N+]([O-])c1ccc(/C=N/Nc2nc(O)cs2)cc1
3	0.5094	1	UIREF-1210; pAct median 5.0742; IC50; Sporosarcina pasteurii; 10.1016/j.bioorg.2015.10.005	ZXQFVBGSFZGXFE-VZUCSPMQSA-N O=[N+]([O-])c1cccc(/C=N/Nc2nc(O)cs2)c1

Validated performance stored in the released bundle

Cross-validation

Validation split	AUC (95% CI)	MCC (95% CI)	Balanced accuracy
document	0.840 (0.777-0.898)	0.071 (-0.027-0.202)	0.536
random	0.959 (0.905-0.992)	0.712 (0.612-0.806)	0.883
scaffold	0.916 (0.872-0.957)	0.394 (0.288-0.526)	0.683

Held-out test and calibration

n	AUC	Brier raw	Brier Platt	Brier isotonic	ECE raw	ECE Platt
179	0.846	0.039	0.062	0.057	0.051	0.132

Conformal validation

alpha	Empirical coverage	Both classes	Empty set	Singleton accuracy
0.05	0.899	0.603	0.000	0.746
0.10	0.838	0.497	0.000	0.678
0.20	0.592	0.095	0.000	0.549

Stage 2: potent versus weak measured activity

Application verdict: REFUSE

No class verdict is reported. Numeric outputs remain available below only as suppressed diagnostics, together with the reasons for refusal.

Label meaning

**Positive class**	potent: pAct_median >= 6
**Negative class**	weak: pAct_median < 6 (MEASURED, real negative)
**What the negative means**	MEASURED AND QUANTITATIVELY WEAK. A real, earned negative.
**Honest interpretation**	MOST DEFENSIBLE STAGE. This is the only stage where both classes were tested and the label means what it says.
**Design**	1110 molecules with >=1 direct quantitative measurement. Both classes measured on the same assay footing.
**Grouping**	primary_document (StratifiedGroupKFold)
**Class counts**	178 positive; 932 negative

Why the application declined to answer

1. Conformal set at alpha=0.10 is {both classes} - UNINFORMATIVE under the frozen_curation convention - the predictor cannot separate the classes for this molecule at the requested confidence.

2. OUTSIDE the applicability domain under the bundle_featurization_note convention: nearest training molecule is only Tanimoto 0.3673, below the preregistered 0.40 floor.

3. The two standardization conventions disagree about this molecule (frozen_curation -> CN(C)c1ccc(/C=N/Nc2nc(O)cs2)cc1; bundle_featurization_note -> CN(C)c1ccc(/C=N/N=C2\NC(=O)CS2)cc1), and the disagreement changes in_applicability_domain, conformal_set_size. A verdict that flips with tautomer convention is not a verdict.

Diagnostics under both standardization conventions

Convention	P(positive)	Nearest-neighbour Tanimoto	Mahalanobis	In domain	Conformal set	Training hit	Usable
frozen_curation	0.2300	0.4528 (floor 0.40)	3.9907 (cutoff 6.1362)	Yes	{both classes} - UNINFORMATIVE	No	No
bundle_featurization_note	0.0933	0.3673 (floor 0.40)	4.1132 (cutoff 6.1362)	No	{negative}	No	No

The reported probability is not the verdict when a stage is refused. It is shown only to document the complete application output.

Nearest training molecules under the frozen convention

Rank	Tanimoto	Training label	Training record	InChIKey and SMILES
1	0.4528	1	UIREF-0671; pAct median 6.9586; IC50; Urease, unspecified origin; 10.1016/j.ejmech.2022.114273	NWRVBUQIDLTUHI-UHFFFAOYSA-N Cc1ccc(C=NNc2nc(-c3ccc(Br)cc3)cs2)cc1
2	0.4151	0	UIREF-0861; pAct median 4.6912; IC50; Canavalia ensiformis; nan	SHNJCBATXVDZCQ-XDHOZWIPSA-N CN(C)c1ccc(/C=N/c2nc(-c3ccccc3)cs2)cc1
3	0.3478	0	UIREF-0679; pAct median 5.8268; IC50; Canavalia ensiformis; 10.1016/j.bmcl.2017.05.019	NYZBLRXVQVPGGS-BHGWPJFGSA-N CN(C)c1ccc(/C=N/NC(=O)Cn2c(C3CC3)nc3cc(Cl)c(Cl)cc32)cc1

Validated performance stored in the released bundle

Cross-validation

Validation split	AUC (95% CI)	MCC (95% CI)	Balanced accuracy
document	0.782 (0.683-0.867)	0.504 (0.364-0.637)	0.686
random	0.900 (0.878-0.918)	0.605 (0.531-0.679)	0.802
scaffold	0.736 (0.633-0.814)	0.238 (0.071-0.367)	0.582

Held-out test and calibration

n	AUC	Brier raw	Brier Platt	Brier isotonic	ECE raw	ECE Platt
222	0.637	0.142	0.144	0.151	0.033	0.037

Conformal validation

alpha	Empirical coverage	Both classes	Empty set	Singleton accuracy
0.05	0.946	0.662	0.000	0.840
0.10	0.932	0.541	0.000	0.853
0.20	0.892	0.324	0.000	0.840

Stage 3: exact structural-alert rule

Exact rule result
Three PAINS/BRENK/NIH alerts were detected. This stage is computed directly from SMARTS rules and is not treated as a predictive model.

Label meaning

**Positive class**	>=1 PAINS/BRENK/NIH structural alert (DETERMINISTIC SMARTS rule)
**Negative class**	0 structural alerts -- NOT "non-toxic"; no toxicity was measured
**What the negative means**	NO PUBLISHED SMARTS PATTERN MATCHED. This is NOT "non-toxic" -- no toxicity assay of any kind enters this label.
**Honest interpretation**	MUST BE DROPPED AS A PREDICTIVE STAGE. Circular on ECFP4 (the fingerprint contains the very substructures the SMARTS match). Replace with the exact rule evaluation and report the alert count. Calling it "toxicity" is unsupported.
**Design**	6000-molecule random sample (SEED=42) of the pooled 29343 molecules across UI-Ref/CLUE/COCONUT/DSSTox. The label is a DETERMINISTIC function of the SMILES: any model of it is re-deriving a rule that RDKit already computes exactly and for free.
**Grouping**	Murcko scaffold (no documents exist for library collections)
**Class counts**	3931 positive; 2069 negative

Why the application declined to answer

1. This stage is NOT a model. Its label is an exact SMARTS function of the structure; the alert list below IS the answer. The fitted model exists only for audit reproducibility and is never consulted.

Diagnostics under both standardization conventions

Convention	P(positive)	Nearest-neighbour Tanimoto	Mahalanobis	In domain	Conformal set	Training hit	Usable
frozen_curation	0.9880	1.0000 (floor 0.40)	3.9547 (cutoff 6.1362)	Yes	{positive}	Yes	Yes
bundle_featurization_note	0.9815	0.3673 (floor 0.40)	3.7807 (cutoff 6.1362)	No	{positive}	No	No

The reported probability is not the verdict when a stage is refused. It is shown only to document the complete application output.

Nearest training molecules under the frozen convention

Rank	Tanimoto	Training label	Training record	InChIKey and SMILES
1	1.0000	1	UIREF-1160; pAct median 5.1367; IC50; Sporosarcina pasteurii; 10.1016/j.bioorg.2015.10.005	YXXKRHZFVGZYRJ-NTUHNPAUSA-N CN(C)c1ccc(/C=N/Nc2nc(O)cs2)cc1
2	0.6170	1	UIREF-1186; pAct median 4.5701; IC50; Sporosarcina pasteurii; 10.1016/j.bioorg.2015.10.005	ZMEAZVJWHIWUFB-VZUCSPMQSA-N O=[N+]([O-])c1ccc(/C=N/Nc2nc(O)cs2)cc1
3	0.4717	1	UIREF-0922; pAct median 4.3231; IC50; Sporosarcina pasteurii; 10.1016/j.bioorg.2015.10.005	UIGFUFWFALPWKF-VZUCSPMQSA-N O=[N+]([O-])c1ccccc1/C=N/Nc1nc(O)cs1

Validated performance stored in the released bundle

Cross-validation

Validation split	AUC (95% CI)	MCC (95% CI)	Balanced accuracy
random	0.930 (0.926-0.934)	0.702 (0.697-0.708)	0.859
scaffold	0.916 (0.909-0.923)	0.665 (0.650-0.686)	0.841

Held-out test and calibration

n	AUC	Brier raw	Brier Platt	Brier isotonic	ECE raw	ECE Platt
1200	0.910	0.126	0.117	0.119	0.089	0.018

Conformal validation

alpha	Empirical coverage	Both classes	Empty set	Singleton accuracy
0.05	0.947	0.330	0.000	0.920
0.10	0.910	0.198	0.000	0.888
0.20	0.810	0.000	0.043	0.847

Stage 4: resemblance to a launched CLUE compound

Application verdict: REFUSE

No class verdict is reported. Numeric outputs remain available below only as suppressed diagnostics, together with the reasons for refusal.

Label meaning

**Positive class**	CLUE Phase == Launched
**Negative class**	CLUE Phase in {Phase clinical, Withdrawn} -- LIBRARY MEMBERSHIP, not approval prediction
**What the negative means**	PRESENT IN THE CLUE LIBRARY WITH A NON-LAUNCHED ANNOTATION. Every molecule here already reached human trials; this is not "unapprovable".
**Honest interpretation**	LIBRARY-MEMBERSHIP TASK, NOT APPROVAL PREDICTION. Applied to a urease hit it answers "does this resemble a launched CLUE drug", which no regulator or trial outcome underwrites. Report only as a similarity score, never as P(approval).
**Design**	4431 CLUE molecules. Conditioning on CLUE membership removes all molecules that never entered development, so the base rate 0.539 is a library artifact.
**Grouping**	Murcko scaffold
**Class counts**	2387 positive; 2044 negative

Why the application declined to answer

1. OUTSIDE the applicability domain under the frozen_curation convention: nearest training molecule is only Tanimoto 0.2281, below the preregistered 0.40 floor.

2. OUTSIDE the applicability domain under the bundle_featurization_note convention: nearest training molecule is only Tanimoto 0.2222, below the preregistered 0.40 floor.

Diagnostics under both standardization conventions

Convention	P(positive)	Nearest-neighbour Tanimoto	Mahalanobis	In domain	Conformal set	Training hit	Usable
frozen_curation	0.5385	0.2281 (floor 0.40)	3.6509 (cutoff 6.1362)	No	{both classes} - UNINFORMATIVE	No	No
bundle_featurization_note	0.5458	0.2222 (floor 0.40)	3.8223 (cutoff 6.1362)	No	{both classes} - UNINFORMATIVE	No	No

The reported probability is not the verdict when a stage is refused. It is shown only to document the complete application output.

Nearest training molecules under the frozen convention

Rank	Tanimoto	Training label	Training record	InChIKey and SMILES
1	0.2281	0	No UI-Ref activity record attached to this stage reference	MHUUDVZSPFRUSK-RQZCQDPDSA-N NC(=O)N/N=C/c1ccc(Oc2ccc(F)cc2)cc1
2	0.2222	0	No UI-Ref activity record attached to this stage reference	ALCVRKZFLFWUAZ-UHFFFAOYSA-N CC(=CC(C)C=CC(O)=NO)C(=O)c1ccc(N(C)C)cc1
3	0.2167	1	No UI-Ref activity record attached to this stage reference	RBTBFTRPCNLSDE-UHFFFAOYSA-N CN(C)c1ccc2nc3ccc(=[N+](C)C)cc-3sc2c1

Validated performance stored in the released bundle

Cross-validation

Validation split	AUC (95% CI)	MCC (95% CI)	Balanced accuracy
random	0.765 (0.752-0.777)	0.397 (0.382-0.409)	0.697
scaffold	0.738 (0.715-0.764)	0.357 (0.329-0.388)	0.677

Held-out test and calibration

n	AUC	Brier raw	Brier Platt	Brier isotonic	ECE raw	ECE Platt
886	0.771	0.197	0.195	0.199	0.062	0.043

Conformal validation

alpha	Empirical coverage	Both classes	Empty set	Singleton accuracy
0.05	0.958	0.713	0.000	0.854
0.10	0.914	0.571	0.000	0.800
0.20	0.840	0.328	0.000	0.761

Application limitations and audit metadata

Refusal policy

If a molecule is OUT of domain, the app MUST refuse to report a probability. If the conformal set is {both classes} or {}, the app MUST report "cannot determine".

Applicability-domain rule

IN DOMAIN requires ECFP4 Tanimoto to nearest training molecule >= 0.40 AND Mahalanobis distance in 25-descriptor space <= sqrt(chi2_0.95(df=25))

Fraction outside the applicability domain

Collection	Outside domain
UI-Ref (self, leave-one-out)	13.6%
CLUE	94.6%
COCONUT	97.0%
DSSTox	92.8%
matched decoys (Stage 1B negatives)	90.4%

Interpretive limitations

Library-membership warning: S4_clinical

LIBRARY MEMBERSHIP, not approval prediction. Conditioned on CLUE membership. Never present as P(approval).

Library-membership warning: S1B_decoy

PRESUMED-negative design. The probability is P(resembles a molecule someone published a urease assay for), NOT P(inhibits urease).

Meaning of UI-Ref membership

UI-Ref is a REPORTED-ACTIVITY set: median pAct 4.72, only 178/1218 reach pAct >= 6. Membership means "measured and reported", never "potent".

Genuine negatives

Only 98 UI-Ref molecules carry a measured-Inactive record; 59 are Inactive-only. There is NO untested-negative class for this target.

Conformal caveat

Coverage was validated on document-/scaffold-disjoint splits. Stage 1A UNDERSHOOTS nominal coverage (0.899 observed vs 0.950 nominal at alpha=0.05): exchangeability fails across publications. Do not claim the guarantee for Stage 1A.

Stage 3

Stage 3 is retained in the bundle ONLY as a deterministic rule evaluator, not a model. Its label is an exact function of the SMILES (PAINS+BRENK+NIH SMARTS match), so the app MUST call the RDKit FilterCatalog directly and report the alert count and names. The fitted model is included for audit reproducibility only and MUST NOT be used for prediction.

Reproducibility metadata

**scikit-learn**	1.9.0
**RDKit**	2026.03.4
**NumPy**	2.4.6
**XGBoost**	3.3.0
**LightGBM**	4.7.0
**Bundle file**	cascade_bundle.joblib

End of report. The interactive application and command-line predictor call the same frozen prediction function used to generate this document.
