# Supplementary material for: Provenance-Associated Confounding in Cross-Collection Comparisons of Urease Inhibitors: A Quantified Reference Scale and a Domain-Aware Alternative
Source: Int J Mol Sci. 2026 Oct 15;27(20):9159. doi: 10.3390/ijms27209159 (PMC13705496; doi:10.3390/ijms27209159)
Supplement: Supplementary file 1 [file ijms-27-09159-s001.zip › Supplementary_Information_revised_tracked_all_daniel_bustos_no_affiliation_edits_final_audited_readable_tables_ChangesAccepted.pdf]

Article

Provenance-Associated Confounding in Cross-Collection Comparisons of Urease Inhibitors: A Quantified Reference Scale and a Domain-Aware Alternative

Javier Farías-Abarca [1,†], Gabriela Urra [1,2], Constanza Henríquez [1], Osvaldo Yáñez [3], Manuel I. Osorio [4,5], Jonathan Palma [6], Luis Morales-Quintana [7], Reynier Suardíaz [8,*], and Daniel Bustos [1,9,*]

[1] Laboratorio de Bioinformática y Química Computacional, Departamento de Medicina Traslacional, Facultad de Medicina, Universidad Católica del Maule, Talca 3480094, Chile. javier.farias@alu.ucm.cl; henriquezvera.95@gmail.com; dbustos@ucm.cl
[2] Doctoral Program in Sciences with a Specialization in Modeling of Chemical and Biological Systems, Faculty of Engineering, Universidad de Talca, Talca 3460000, Chile; gabriela.urra@utalca.cl
[3] Centro de Modelación Ambiental y Dinámica de Sistemas (CEMADIS), Facultad de Ingeniería y Negocios, Universidad de Las Américas, Santiago 7500975, Chile. oyanez@udla.cl
[4] Facultad de Odontología, Universidad Andrés Bello, Santiago Chile, Echaurren 237, Santiago 8370133, Chile
[5] Centro de Investigación Biomédica, Facultad de Medicina, Universidad Diego Portales, Ejército 141, Santiago 8370007, Chile. manuel.osorio@unab.cl
[6] Facultad de Ingeniería, Universidad de Talca, Maule, Curicó 3349001, Chile; jonathan.palma@utalca.cl
[7] Multidisciplinary Agroindustry Research Laboratory, Instituto de Ciencias Biomédicas, Facultad de Ciencias de La Salud, Universidad Autónoma de Chile, Cinco Poniente #1670, Región del Maule, Talca 7500912, Chile; luis.morales@uautonoma.cl
[8] Departamento de Química Física, Facultad de Ciencias Químicas, Universidad Complutense de Madrid, 28040 Madrid, Spain. reysuard@ucm.es
[9] Laboratorio de Genómica, Bioinformática y Biosensores Aplicados, Facultad de Medicina, Universidad Católica del Maule, Talca 3480094, Chile
* Correspondence: dbustos@ucm.cl (D.B.) and reysuard@ucm.es (R.S.)

This document contains Figures S1-S10 and Tables S1-S43. Every item is regenerated from the deposited data by a numbered script in the release repository; the mapping from each figure to its script and input tables is given in the repository documentation. Figures S8-S10 and Tables S33-S43 accompany the analyses of Sections 2.8 to 2.11 of the main text.

Supplementary Figures

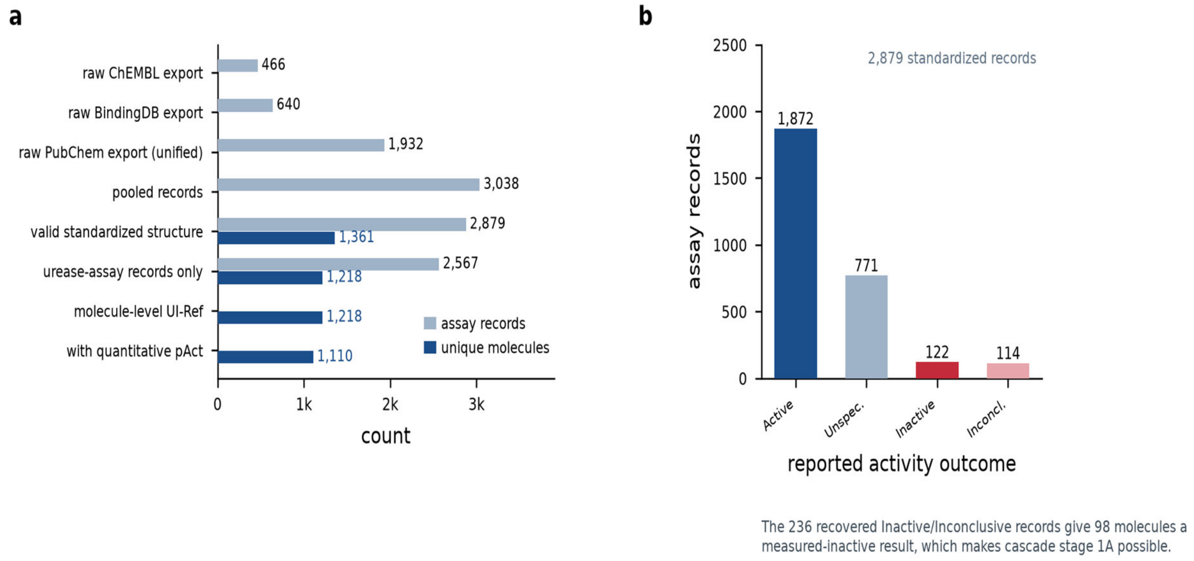

Figure S1. Curation detail: record- and molecule-level counts at every standardization step, and the activity outcomes discarded by the uncorrected pipeline. (a) Records and unique molecules surviving each step, from the three primary exports to the 1,110 molecules with a quantitative pAct. (b) Reported activity outcome for the 2,879 standardized records. Excluding records reported as inactive or inconclusive discards the only measured negatives available; recovering the 236 such records yields 98 molecules with a measured-inactive record and makes stage 1A of the cascade possible. The standardization defect affecting nine molecules is described in the text rather than plotted: for those molecules the depositor SMILES carried a formally cationic phosphorus, [P+](=O)O, which the frozen standardizer rewrote to [PH+](=O)=O, a distinct species with a different InChIKey. RDKit parses both without error, so the nine molecules changed identity and were dropped from every model without an exception being raised. Repairing them to the neutral pentavalent phosphinic acid raises descriptor completeness from 0.9926 to 1.000. All nine are phosphinic-acid inhibitors from a single document, with pAct between 3.66 and 4.79 across 21 records.

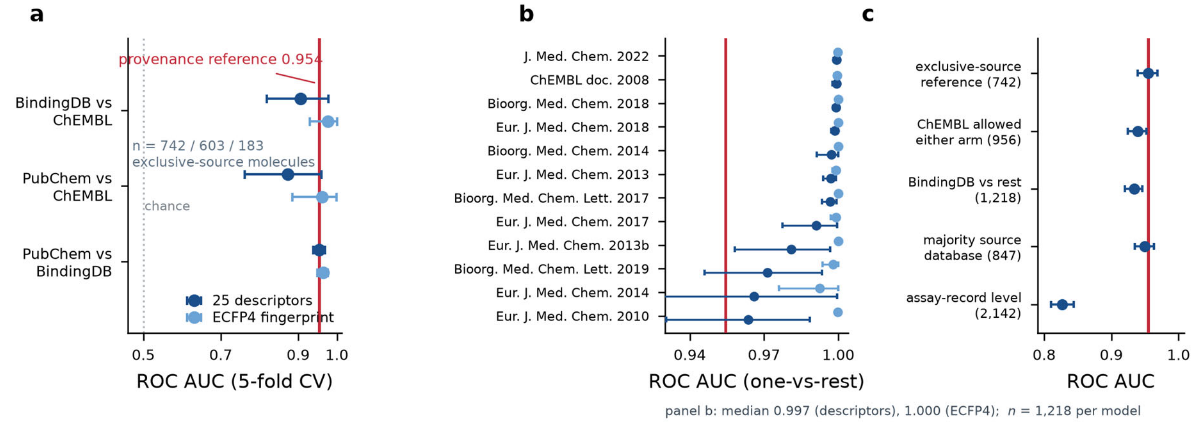

Figure S2. Per-model benchmark detail for the five cascade stages, showing every combination of model and feature block with bootstrap confidence intervals. (a-e) ROC AUC with 2,000-resample bootstrap intervals for a random forest, XGBoost, LightGBM, logistic regression, a nearest-neighbor Tanimoto baseline, the majority-class rule and a y-scrambled random forest, each evaluated on the descriptor block, the ECFP4 block and their union, under the grouping named in the panel title. The

three marks denote the feature blocks: 25 RDKit descriptors, a 2,048-bit Morgan fingerprint of radius 2 (ECFP4), and their union. Stages 1A, 1B and 2 use StratifiedGroupKFold on source document; stages 3 and 4 group on Murcko scaffold, because no source document exists for the CLUE library or for the alert-labeled sample. Intervals are 2,000-resample bootstraps on pooled out-of-fold predictions. The y-scrambled forest and the majority-class rule provide the two reference baselines. Tree ensembles on the union block give the highest AUC at every stage, but the margin over the nearest-neighbor Tanimoto baseline is small at stages 1B and 2, which indicates that much of the available signal is similarity to training compounds.

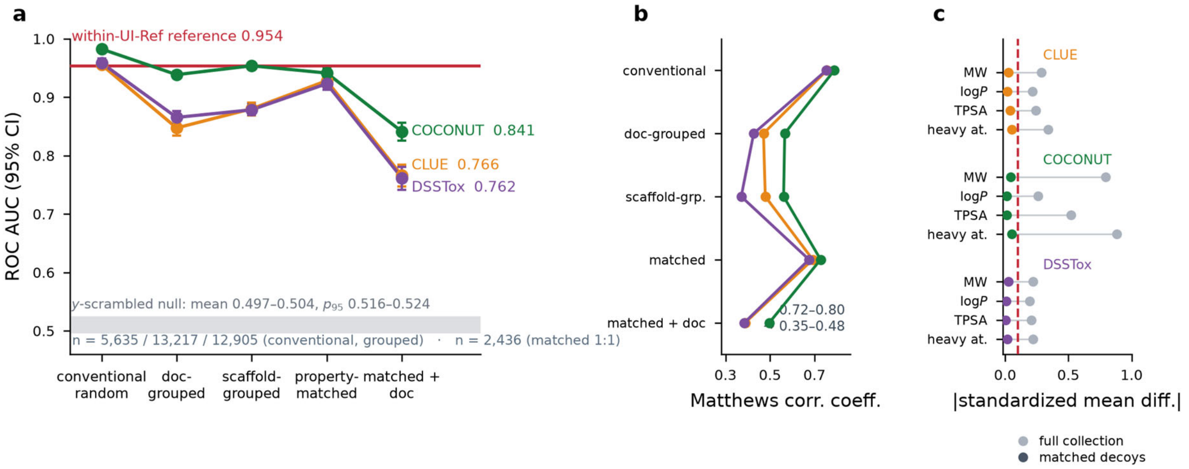

Figure S3. Label-permutation controls for every design. (a) For each of the 17 designs, the permutation null (mean, 95th percentile and maximum over 100 label permutations) against the observed AUC. Every observed value exceeds the 95th percentile of its own null, and the permutation p-value is 0.0099 throughout (1 of 101, including the observed value). (b) Null mean against null 95th percentile across designs: means span 0.497-0.504 and 95th percentiles span 0.516-0.603, the widest nulls belonging to the small-sample provenance designs. The two provenance rows are the same models shown as the reference scale in Figure 2.

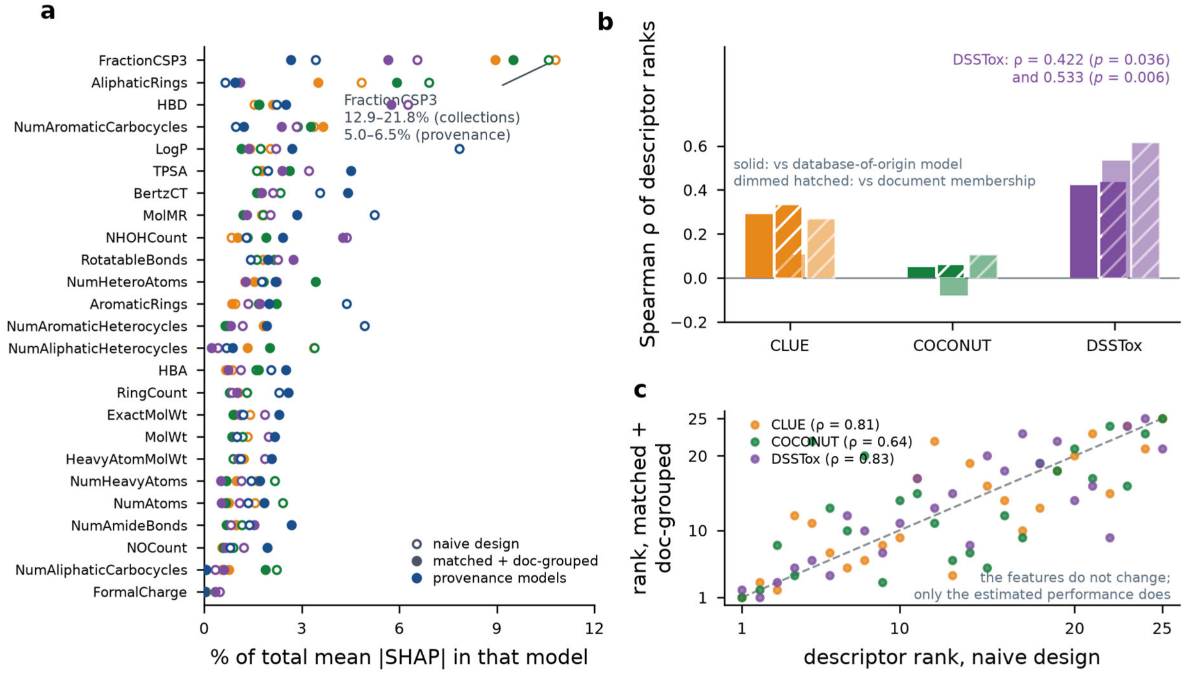

Figure S4. SHAP attributions for the collection and provenance classifiers. (a) Share of the total mean absolute SHAP value carried by each of the 25 descriptors in eight models: the three collection

https://doi.org/10.3390/xxxxx

comparisons under the conventional design and under the matched, document-grouped design, plus the two provenance models. FractionCSP3 ranks first or second and carries 12.9-21.8% of the total attribution in the six collection models, but ranks sixth at 5.0-6.5% in the provenance models. Rotatable-bond count, emphasized in the uncorrected analysis, is never leading (rank 4-16, 2.7-6.6%). (b) Spearman rank correlation of the descriptor importances of each collection model against the two provenance models. The conventional DSSTox model correlates with the database-of-origin classifier at rho = 0.422 (p = 0.036) and with document membership at rho = 0.533 (p = 0.006). (c) Descriptor rank under the conventional design against rank under the matched, document-grouped design (rho = 0.81, 0.64 and 0.83), showing that correcting the design changes the performance estimate substantially more than it changes the feature ranking.

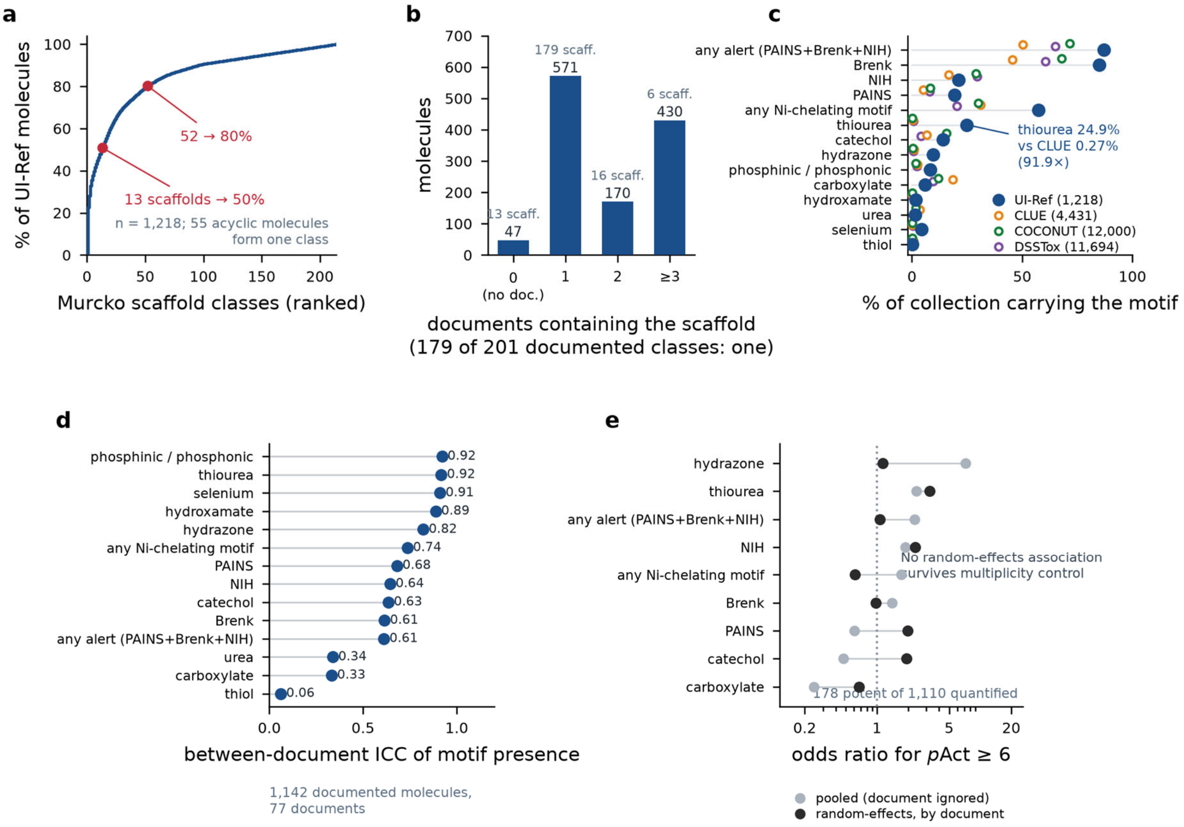

Figure S5. Near-duplicate leakage across split boundaries. (a) Percentage of test molecules with a training-set neighbor at ECFP4 Tanimoto >= 0.7, by split type and by which side of the comparison the test molecules come from. Under random splitting, 64% of UI-Ref molecules have such a neighbor; document-grouped splitting reduces this to 18% and scaffold-grouped splitting to 15%. The external-collection sides are far less affected, so random splitting inflates performance asymmetrically, and it does so on the UI-Ref class. (b) Median maximum Tanimoto similarity to the training set against the fraction of pairs at Tanimoto >= 0.9. Circles denote the pooled test sets of the three pairwise comparisons and squares denote UI-Ref alone; every grouped split lies in the low-leakage region.

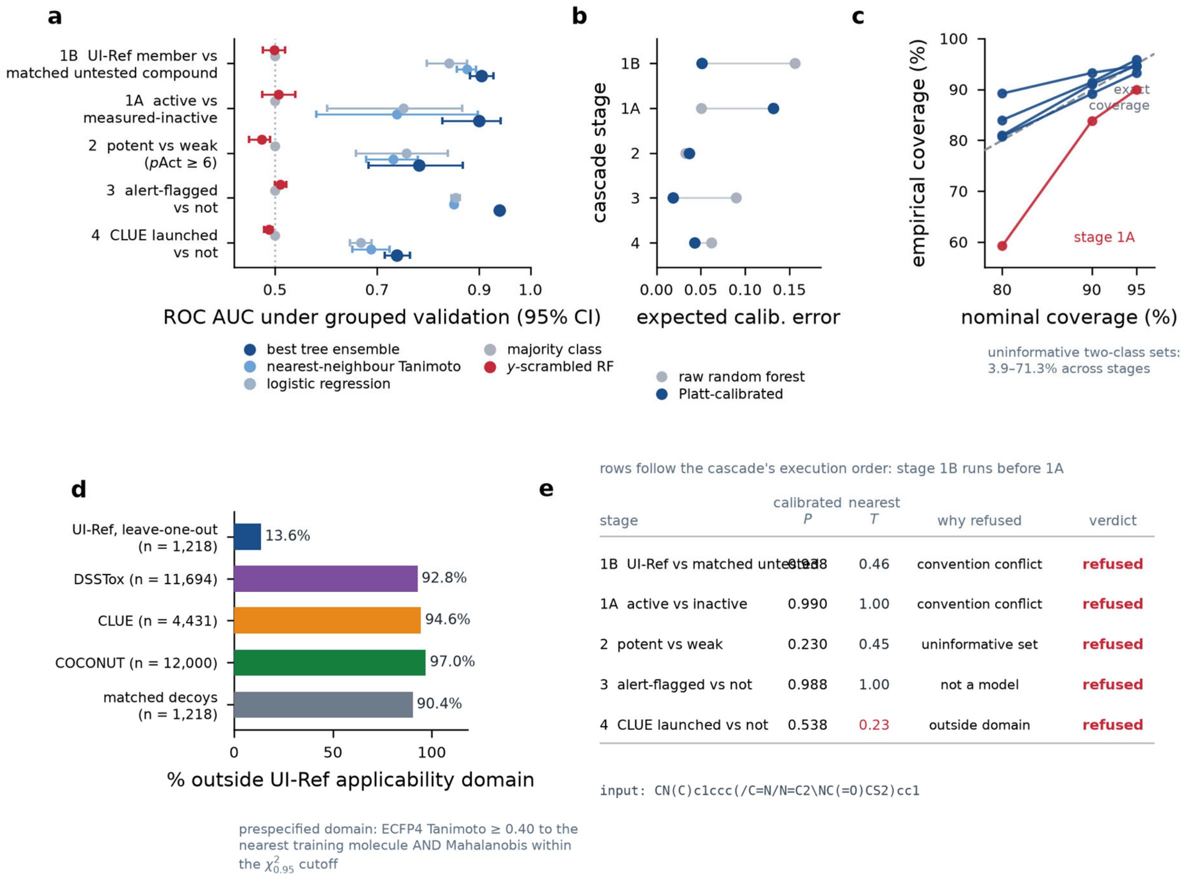

Figure S6. Distribution of structural alerts within source documents. (a) Prevalence of each alert class and metal-chelating motif in each of the 48 documents contributing at least 10 molecules (1,020 of the 1,142 documented molecules). Of the 672 document-by-motif cells, 84% lie below 5% or above 95%, which is the reason motif-potency associations pooled across documents are confounded by publication. (b) Molecule count per document, ranked; document identifiers are resolved in Table S31. The between-document intraclass correlation spans 0.06 (thiol) to 0.92 (phosphinic/phosphonic and thiourea).

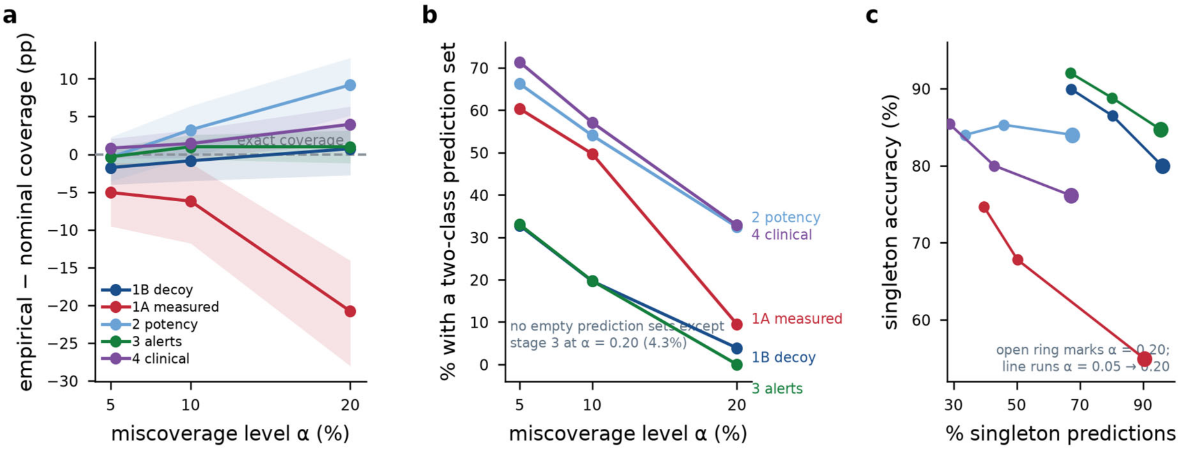

Figure S7. Conformal coverage per stage and miscoverage level. (a) Empirical minus nominal coverage at alpha = 0.05, 0.10 and 0.20 with bootstrap bands. Four stages are within error of nominal coverage on this split, whereas stage 1A falls short by 5 to 21 percentage points, because only 59 measured-inactive molecules are available for calibration and testing. Figure S10 and Table S40 show that this shortfall belongs to the split rather than to the stage: stage 1A coverage is not stable across publications. Exchangeability between calibration and test document groups is also rejected

for every model-based stage, so the coverage reported here is an empirical property of this corpus and not a guarantee. (b) Fraction of test molecules receiving a two-class, and therefore uninformative, prediction set. At alpha = 0.05 this reaches 33-71% depending on the stage; no empty sets occur except at stage 3 with alpha = 0.20 (4.3%). (c) Accuracy on singleton predictions against the fraction of predictions that are singletons, tracing alpha from 0.05 to 0.20 for each stage. Accepting more decisions reduces their accuracy, which is the trade-off any predictive system of this kind must state explicitly.

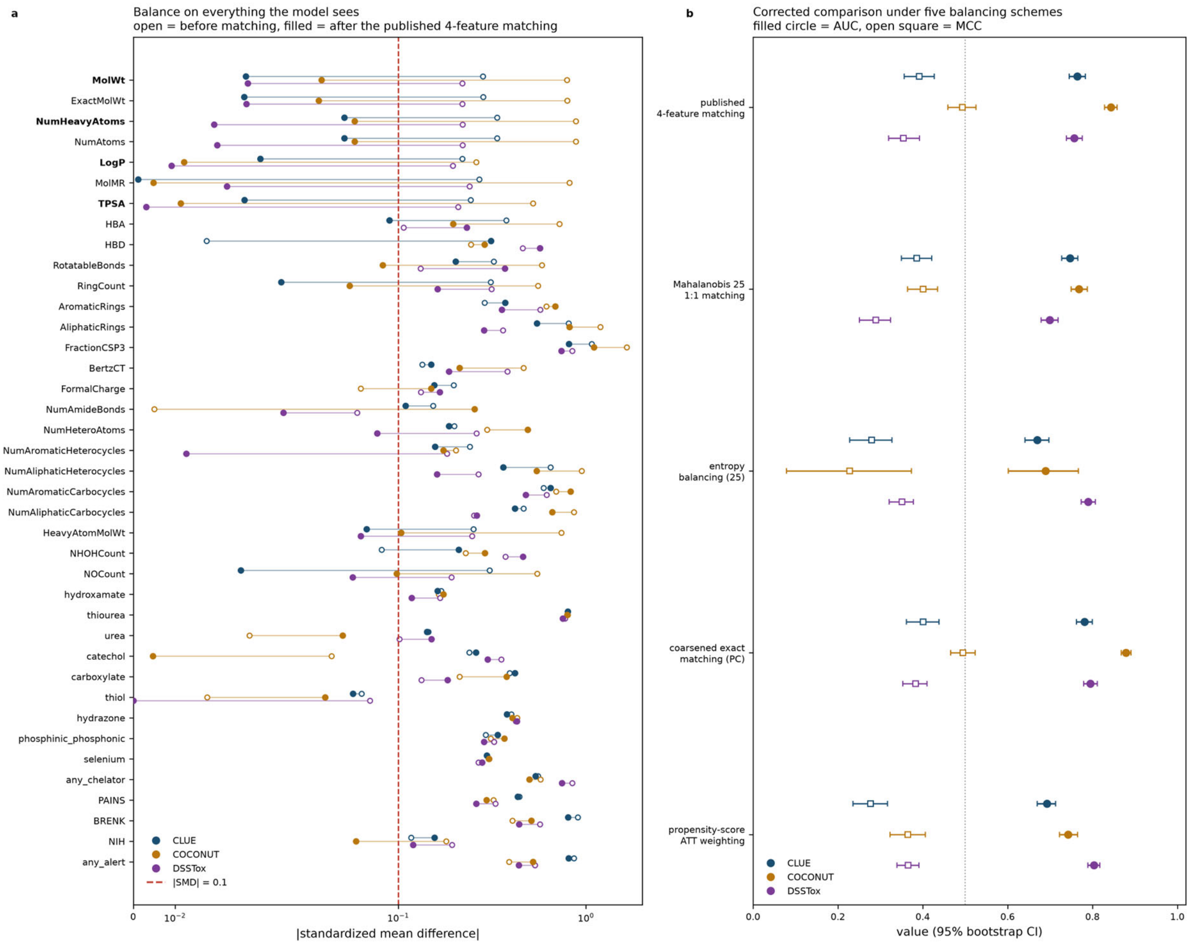

Figure S8. Covariate balance and its sensitivity to the balancing scheme. Left panel (a): absolute standardized mean difference for all 25 physicochemical descriptors and the 14 substructure indicators, before and after the published 1:1 nearest-neighbor matching on molecular weight, cLogP, TPSA and heavy-atom count, for each external collection. The dashed line marks the 0.1 balance criterion; the four matching covariates reach a maximum absolute value of 0.059, whereas 25 to 27 of the 39 covariates the models can use remain above 0.1, including thiourea (0.75-0.80) and the presence of any chelating motif (0.50-0.75). Right panel (b): corrected AUC and MCC with bootstrap confidence intervals under five balancing schemes applied to the full 25-descriptor block, all with document-grouped cross-validation. n = 1,218 UI-Ref molecules against 1,218 matched comparison molecules in the two 1:1 matching schemes; the weighting and coarsened-exact schemes retain 1,192 to 1,218 UI-Ref molecules against 2,804 to 12,000 weighted comparison molecules (Table S35).

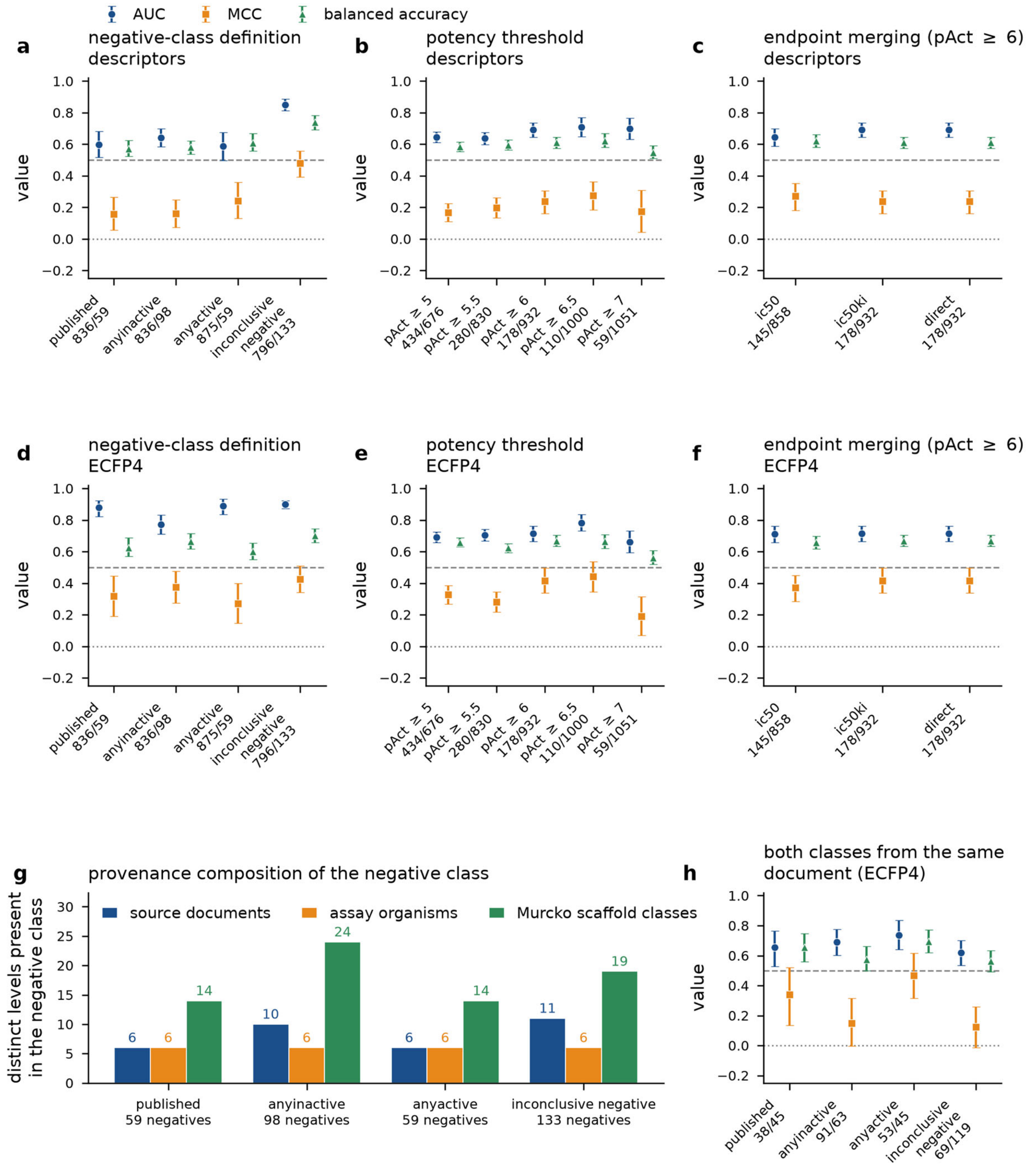

Figure S9. Dependence of the measured-activity stage on its label definition. Performance under document-grouped cross-validation across the four negative-class definitions, the five potency thresholds and the three endpoint-merging strategies, on descriptors and on ECFP4, with bootstrap confidence intervals, together with the provenance composition of the negative class in each setting. The AUC moves from 0.586 to 0.852 on descriptors and from 0.772 to 0.899 on fingerprints as a function of the negative-class definition alone. Restricting the comparison to the six documents that contain both classes leaves 83 molecules and gives a descriptor-model AUC of 0.393 (95% CI 0.270-0.520), below chance.

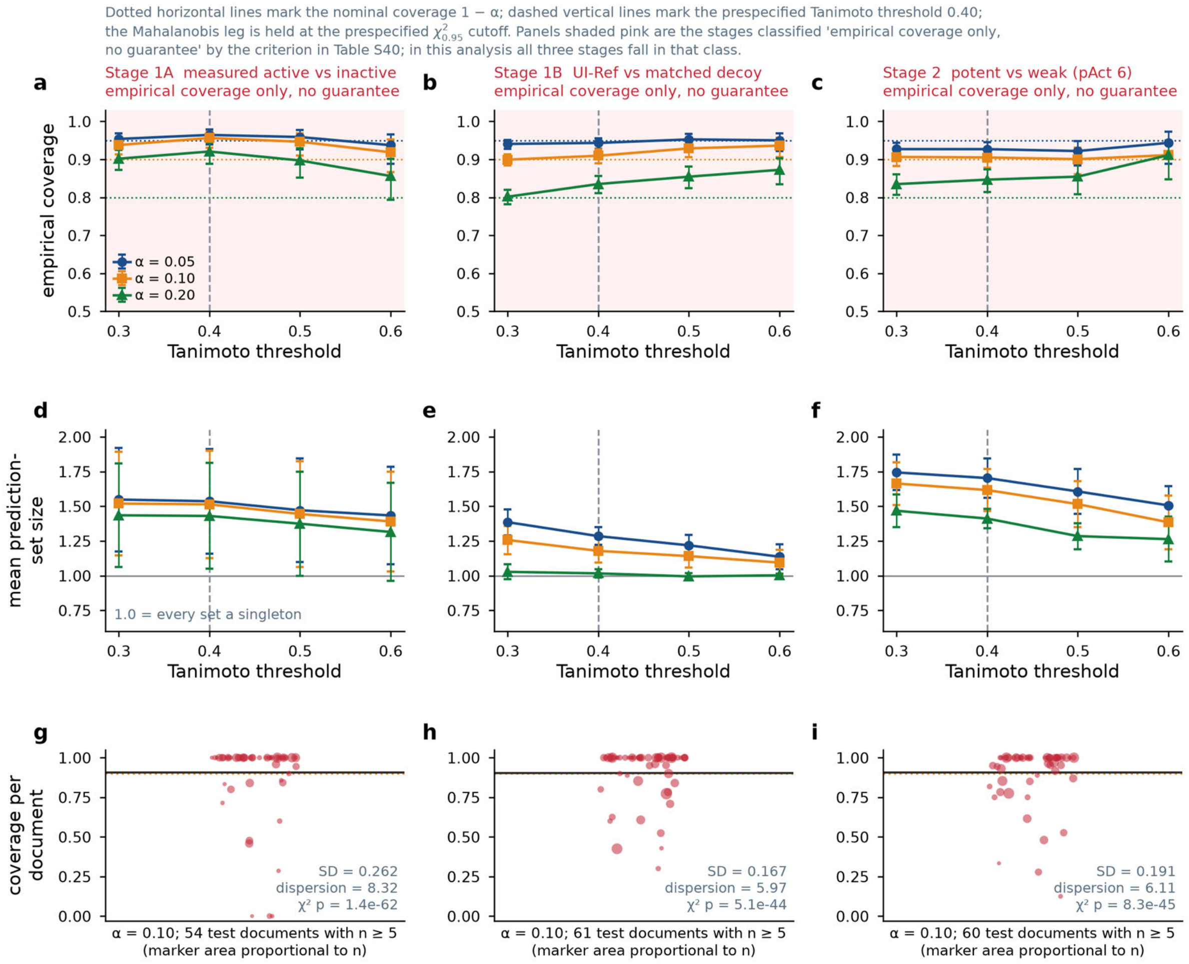

Figure S10. Conformal coverage and prediction-set size across the applicability-domain grid. Empirical coverage and mean prediction-set size at alpha = 0.05, 0.10 and 0.20 as a function of the domain threshold, for stages 1A, 1B and 2, with the nominal level marked and stages lacking exchangeability distinguished. Dispersion is the standard deviation over five rotating document-grouped splits. Mean set size decreases monotonically as the threshold tightens, from 1.257 to 1.094 for stage 1B and from 1.664 to 1.384 for stage 2 at alpha = 0.10, and the singleton fraction rises correspondingly from 74.3% to 90.6% and from 33.6% to 61.6%.

Supplementary Tables

Table S1. Curation and standardization flow. Assay records and unique molecules surviving each step from the raw PubChem, BindingDB and ChEMBL exports to the 1,110 UI-Ref molecules with a quantitative pAct. *(8 rows × 4 columns; tableS1_curation_flow.csv)*

Table S2. As-published value versus corrected value for every quantity re-examined in this audit, with the reason the original value was wrong. *(6 rows × 3 columns; tableS2_aspublished_vs_corrected.csv)*

Table S3. Audit findings. Each methodological defect identified in the original work, its severity, the evidence, and the remedy adopted here. *(10 rows × 6 columns; tableS3_audit_findings.csv)*

Table S4. Corrections registry. 15 numerical claims made in this project's own analysis prose that were found to be inconsistent with the underlying tables, with the corrected value and how it was verified. *(8 rows × 5 columns; tableS4_corrections_registry.csv)*

Table S5. Provenance reference scale. Random-forest classifiers trained inside UI-Ref alone to predict database of origin (three pairs, exclusive-source molecules) and source document (one-vs-rest over the twelve largest documents), on 25 descriptors and on ECFP4.

Database-origin comparisons use 5-fold stratified cross-validation; document one-versus-rest comparisons use 3-fold stratified cross-validation. Confidence intervals use 2,000 bootstrap resamples. *(34 rows × 13 columns; tableS5_provenance_floor.csv)*

Table S6. All pairwise UI-Ref versus external-collection designs. For each of the three comparisons and six designs: n, positives, ROC AUC with bootstrap interval, per-fold mean and standard deviation, MCC and F1. *(18 rows × 11 columns; tableS6_pairwise_designs.csv)*

Table S7. Standardized mean differences for the twelve matching covariates before and after nearest-neighbor property matching on molecular weight, LogP, TPSA and heavy-atom count. *(12 rows × 7 columns; tableS7_matching_smd.csv)*

Table S8. Y-scrambling permutation nulls. For each of the 17 designs, 100 label permutations: null mean, standard deviation, 95th percentile, maximum, the real AUC and the permutation p value. *(17 rows × 10 columns; tableS8_yscrambling.csv)*

Table S9. Train/test near-duplicate leakage. Median maximum ECFP4 Tanimoto to the training set and the fraction of test molecules at Tanimoto ≥ 0.7 and ≥ 0.9, by split type and comparison side. *(30 rows × 8 columns; tableS9_leakage.csv)*

Table S10. Mean absolute SHAP value and within-model rank for all 25 descriptors in eight models: three collection comparisons under conventional and matched + document-grouped designs, and two provenance models. *(25 rows × 17 columns; tableS10_shap_comparison.csv)*

Table S11. Spearman rank correlation of each collection model's descriptor importances against the database-of-origin and document-membership provenance models, with p values. *(16 rows × 5 columns; tableS11_shap_rank_correlations.csv)*

Table S12. k-nearest-neighbor composition and enrichment for k = 1, 5 and 25 in four spaces (full ECFP4, PCA, UMAP, t-SNE) under native unequal-n and equal-n sampling, with bootstrap intervals over 20 draws. *(96 rows × 12 columns; tableS12_neighbourhood_enrichment.csv)*

Table S13. Nearest-neighbor ECFP4 Tanimoto from each UI-Ref molecule to each collection: mean, quartiles, median and the fraction at Tanimoto ≥ 0.4 and ≥ 0.7. *(4 rows × 11 columns; tableS13_nn_tanimoto.csv)*

Table S14. Adjusted mutual information between Butina clusters and collection, UI-Ref source document, or assay organism, at three clustering cut-offs. *(9 rows × 10 columns; tableS14_cluster_ami.csv)*

Table S15. Silhouette coefficients for collection labels and for UI-Ref source-document labels, in the full ECFP4 space and in the UMAP embedding. *(15 rows × 6 columns; tableS15_silhouette.csv)*

Table S16. All 214 Bemis–Murcko scaffold classes of UI-Ref with molecule count, number of distinct source documents, median pAct, acyclicity flag and cumulative coverage. *(214 rows × 8 columns; tableS16_scaffolds.csv)*

Table S17. SMARTS validation. Positive and negative control molecules for each of the 11 SMARTS patterns used, with the expected and observed match. *(11 rows × 7 columns; tableS17_smarts_validation.csv)*

Table S18. Structural-alert and metal-chelator prevalence in UI-Ref and in each external collection. *(4 rows × 16 columns; tableS18_alerts_by_collection.csv)*

Table S19. Structural-alert prevalence per source document for the documents contributing at least 10 molecules, with the identifiers used in Figure S6. *(61 rows × 17 columns; tableS19_alerts_by_document.csv)*

Table S20. Between-document intraclass correlation for each motif, with the number of documents contributing and the within- and between-document variance components. *(14 rows × 6 columns; tableS20_motif_variance_by_document.csv)*

Table S21. Motif–potency odds ratios: pooled estimate with confidence interval, Mantel–Haenszel estimate stratified by source document, number of informative strata and whether the direction reverses. *(14 rows × 15 columns; tableS21_motif_potency_or.csv)*

Table S22. Chemical-space summary statistics per collection: size, descriptor ranges and the diversity measures used in Figure 4. *(1 rows × 13 columns; tableS22_chemspace_summary.csv)*

Table S23. Cascade label semantics. For each of the five stages: the question asked, what the positive and negative classes mean, class sizes, what the negatives are drawn from, the grouping variable and the scope-qualified verdict. *(5 rows × 11 columns; tableS23_cascade_label_semantics.csv)*

Table S24. Full cascade benchmark. Every stage × split scheme × model × feature block with AUC and bootstrap interval, MCC, balanced accuracy and Brier score. *(195 rows × 17 columns; tableS24_cascade_benchmark.csv)*

Table S25. Probability calibration per stage: raw, Platt-scaled and isotonic, against a train-prevalence baseline, with Brier score and expected calibration error and their bootstrap intervals. *(20 rows × 15 columns; tableS25_calibration.csv)*

Table S26. Applicability domain. Coverage of each collection under the prespecified ECFP4 Tanimoto ≥ 0.40 criterion and a Mahalanobis criterion, plus inside-versus-outside performance per stage. *(35 rows × 20 columns; tableS26_applicability_domain.csv)*

Table S27. Conformal prediction per stage and α: nominal and empirical coverage with interval, singleton fraction and accuracy, two-class fraction and empty-set fraction. *(15 rows × 15 columns; tableS27_conformal_coverage.csv)*

Table S28. Error propagation through the sequential cascade: surviving fraction and cumulative reliability at each stage. *(6 rows × 17 columns; tableS28_cascade_propagation.csv)*

Table S29. Worked trace of the prototype compound through every cascade stage: nearest-neighbor Tanimoto, domain verdict, and the prediction that would have been returned had the domain check been omitted. *(5 rows × 22 columns; tableS29_example_compound_trace.csv)*

Table S30. Descriptor dictionary. The 25 RDKit descriptors with definition, unit, generating function, caveats and their UI-Ref mean, standard deviation, minimum, median and maximum. *(25 rows × 11 columns; tableS30_descriptor_dictionary.csv)*

Table S31. Source-document key. All 77 named documents with DOI or ChEMBL identifier, journal, year, molecule count, number with a quantitative pAct, median pAct, potent count, assay organism, and the identifier used in Figure S6. *(77 rows × 11 columns; tableS31_document_key.csv)*

Table S32. Verified references. All 43 references with DOI, verification status and ACS-format citation string; the independent per-reference verdicts are given in Table S43. *(43 rows × 17 columns; tableS32_references_verified.csv)*

Table S33. Motif SMARTS validation. The frozen SMARTS pattern for each of the nine functional motifs, the outcome of all 11 positive and negative controls of Table S17, and the prevalence of every motif and alert set on the 1,110 molecules with a quantitative pAct beside the count reported in sheet S21. All 11 controls pass on every run. The two counts differ by -11 to +1 molecules across the nine motifs, the largest difference being catechol, and by -20 for any chelator, -14 for PAINS, -12 for the NIH set, -5 for any alert and -1 for BRENK; the frozen patterns released with the analysis package are the ones used throughout this work. *(25 rows × 12 columns; tableS33_motif_smarts_validation.csv)*

Table S34. Task-matched null models. For each collection comparison, the observed corrected model and each of the four null models fitted inside the same design, with AUC and bootstrap interval, the paired bootstrap difference against the observed model, and the DeLong statistic and p-value where both models score the same molecules; also the within-

library provenance-label estimates for CLUE and DSSTox and the provenance-stratified conditional AUC. *(27 rows × 31 columns; tableS34_task_matched_nulls.csv)*

Table S35. Covariate balance and balancing sensitivity. Standardized mean differences before and after matching for all 25 descriptors and all 14 substructure indicators in each collection, with covariates above 0.1 flagged; corrected AUC and MCC with bootstrap intervals under five balancing schemes with their balance, overlap and effective-sample-size diagnostics; and the comparison of retained against discarded external molecules across the substructure indicators with Benjamini-Hochberg correction. *(252 rows × 58 columns; tableS35_balance_sensitivity.csv)*

Table S36. Multilevel validation. Normalized conditional entropies of organism, scaffold and endpoint given source document; grouped cross-validation under publication-preserving and factor-specific groupings; leave-one-document-out evaluation; fold-integrity diagnostics showing why composite document-organism and document-organism-scaffold AUCs were withdrawn; counts of within-document comparisons; and mixed-effects variance components with standard errors for document and organism, fitted separately, crossed and nested. *(tableS36_multilevel_validation.csv)*

Table S37. Hierarchical motif-potency associations. For each of the 14 motifs: the number of informative document strata, documents, organisms and scaffold classes; pooled, Mantel-Haenszel, exact conditional, DerSimonian-Laird random-effects and Bayesian hierarchical estimates with intervals; tau-squared, Cochran's Q, I-squared and the prediction interval; the leave-one-document-out range and the most influential publication; and Benjamini-Hochberg-adjusted p-values. This sheet supersedes Table S21 for all inferential purposes. *(14 rows × 59 columns; tableS37_hierarchical_motifs.csv)*

Table S38. Stage 1A label sensitivity. Performance under document-grouped cross-validation across four negative-class definitions, five potency thresholds, three endpoint-merging strategies and two representations, with the provenance composition of the negative class, the within-document restriction, and the positive-unlabeled analysis including the homogeneity test of the selected-completely-at-random assumption. *(40 rows × 27 columns; tableS38_stage1a_labels.csv)*

Table S39. Applicability-domain sensitivity. Out-of-domain fraction with Wilson intervals for UI-Ref, the three external collections and the matched decoys, across the full cross of Tanimoto threshold and chi-square quantile and each criterion alone; the same under six fingerprints and eight covariance or descriptor-space variants; and the conditioning, variance-inflation and normality diagnostics of the 25-descriptor covariance matrix. *(399 rows × 29 columns; tableS39_domain_sensitivity.csv)*

Table S40. Conformal sensitivity and exchangeability. In-domain sample size, stage AUC and MCC, empirical coverage, mean prediction-set size and the singleton, two-class and empty-set fractions for every domain definition and miscoverage level, over five rotating document-grouped splits; the Kolmogorov-Smirnov and Mann-Whitney exchangeability tests; the per-document coverage breakdown with its homogeneity test and dispersion ratio; and the four-condition classification of each stage reported as Table 3. *(387 rows × 65 columns; tableS40_conformal_sensitivity.csv)*

Table S41. Source-resource scope and curation. For each of the six resources: the version used, primary scope and purpose, content type, approximate size at that version, how compounds enter, the structure curation the resource performs, whether and how activity data are normalized, the descriptors or computed properties the resource provides including the complete enumeration of the 49 PubChem computed properties, the identifiers used, the licence, the contribution to this study, and the source of each fact with its retrieval date. This sheet is the full form of Table 1. *(6 rows × 14 columns; tableS41_database_scope.csv)*
